# Altered translation elongation contributes to key hallmarks of aging in the killifish brain

**DOI:** 10.1126/science.adk3079

**Published:** 2025-07-31

**Authors:** Domenico Di Fraia, Antonio Marino, Jae Ho Lee, Erika Kelmer Sacramento, Mario Baumgart, Sara Bagnoli, Till Balla, Felix Schalk, Stephan Kamrad, Rui Guan, Cinzia Caterino, Chiara Giannuzzi, Pedro Tomaz da Silva, Amit Kumar Sahu, Hanna Gut, Giacomo Siano, Max Tiessen, Eva Terzibasi-Tozzini, Eugenio F. Fornasiero, Julien Gagneur, Christoph Englert, Kiran R. Patil, Clara Correia-Melo, Danny D. Nedialkova, Judith Frydman, Alessandro Cellerino, Alessandro Ori

**Affiliations:** 1 Leibniz Institute on Aging–Fritz Lipmann Institute (FLI), Jena, Germany.; 2 Department of Biology, Stanford University, Stanford, CA, USA.; 3 BIO@SNS, Scuola Normale Superiore, Pisa, Italy.; 4 Mechanisms of Protein Biogenesis, Max Planck Institute of Biochemistry, Martinsried, Germany.; 5 MRC Toxicology Unit, University of Cambridge, Cambridge, UK.; 6 School of Computation, Information and Technology, Technical University of Munich, Garching, Germany.; 7 Munich Center for Machine Learning, Munich, Germany.; 8 Stazione Zoologica Anton Dohrn, Naples, Italy.; 9 Department of Neuro- and Sensory Physiology, University Medical Center Göttingen, Göttingen, Germany.; 10 Department of Life Sciences, University of Trieste, Trieste, Italy.; 11 Computational Health Center, Helmholtz Center Munich, Neuherberg, Germany.; 12 Institute of Human Genetics, School of Medicine, Technical University of Munich, Munich, Germany.; 13 Institute of Biochemistry and Biophysics, Friedrich Schiller University Jena, Jena, Germany.; 14 Department of Bioscience, TUM School of Natural Sciences, Technical University of Munich, Garching, Germany.; 15 Glenn Laboratories for the Biology of Aging, Stanford University, Stanford, CA, USA.

## Abstract

Aging is a major risk factor for neurodegeneration and is characterized by diverse cellular and molecular hallmarks. To understand the origin of these hallmarks, we studied the effects of aging on the transcriptome, translatome, and proteome in the brain of short-lived killifish. We identified a cascade of events in which aberrant translation pausing led to altered abundance of proteins independently of transcriptional regulation. In particular, aging caused increased ribosome stalling and widespread depletion of proteins enriched in basic amino acids. These findings uncover a potential vulnerable point in the aging brain’s biology—the biogenesis of basic DNA and RNA binding proteins. This vulnerability may represent a unifying principle that connects various aging hallmarks, encompassing genome integrity, proteostasis, and the biosynthesis of macromolecules.

## Abstract (summary)

**INTRODUCTION:**

Aging brains are characterized by a series of molecular and cellular changes known as aging hallmarks. Among these, a decline in protein homeostasis (proteostasis) marked by reduced clearance and increased protein damage and aggregation has received particular attention as a plausible link between brain aging and those neurodegenerative diseases also characterized by protein aggregation. A notable phenomenon in brain aging is a loss of concordance between mRNA and protein levels, whereby age-linked changes in mRNA do not necessarily lead to proportional changes in protein levels. In this study, we set out to investigate the causes of this “protein-transcript decoupling” and how impaired protein synthesis might contribute to other hallmarks of brain aging.

**RATIONALE:**

We used the short-lived African turquoise killifish, which exhibits a naturally compressed life span and accelerated brain aging, to undertake a comprehensive investigation of age-related decline in brain proteostasis. We compared young, adult, and old killifish brains at the levels of amino acid concentrations, tRNAs, mRNAs (transcriptome), actively translated mRNAs (translatome), proteins (proteome), protein modifications [phosphorylation (Ph), ubiquitylation (Ub), and acetylation (Ac)], and protein solubility and subcellular localization. We also tested whether reduced protein degradation caused by proteasome impairment contributes to protein-transcript decoupling and other aging hallmarks in the killifish brain. Our comprehensive design allowed us to pinpoint aging-vulnerable steps in protein biogenesis and reveal mechanisms connecting proteostasis decline to other aging hallmarks.

**RESULTS:**

We observed alterations in all molecular signatures investigated, ranging from amino acid concentrations to protein solubility and localization. A clear pattern of proteostasis dysfunction emerged: Although the synthesis of some proteins was enhanced, there was a widespread reduction of proteins enriched in positively charged (basic) amino acids. Notably, many DNA and RNA binding proteins exhibited reduced abundance in old brains, decreasing at the protein but not the transcript levels. Ribosome profiling (Ribo-seq) revealed that brain aging increased ribosome stalling. Accordingly, ribosome collisions were more frequent in old brains. Crucially, stalling events occurred disproportionately on stretches enriched in lysine and arginine codons, thus affecting translation of mRNAs encoding proteins enriched in these basic amino acids, leading to a decline in their protein levels in old brains. Aging-affected proteins included ribosomal subunits and proteins involved in DNA repair, transcription, chromatin maintenance, and RNA splicing and export, which all mediate processes influenced by aging. Ribosome stalling was also associated with increased protein insolubility, likely owing to nascent polypeptide misfolding. Partial proteasome inhibition affected aging hallmarks distinct from those linked to translation dysfunction and primarily influenced lysosomes and mitochondria.

**CONCLUSION:**

This work identifies altered translation elongation and impaired protein biogenesis as hallmarks of brain aging in a short-lived vertebrate. Increased ribosome pausing is proposed as a key mechanism contributing to the mismatch between mRNA and protein changes observed in aged brains, leading to proteome aging by altering the production of proteins essential for genome integrity, mRNA transcription, splicing, protein synthesis, and mitochondrial function. This mechanism thereby links translation and proteostasis decline to other hallmarks of aging and may also be implicated in neurodegenerative diseases where similar ribosome dysfunction and protein misfolding occur.

## Graphical Abstract

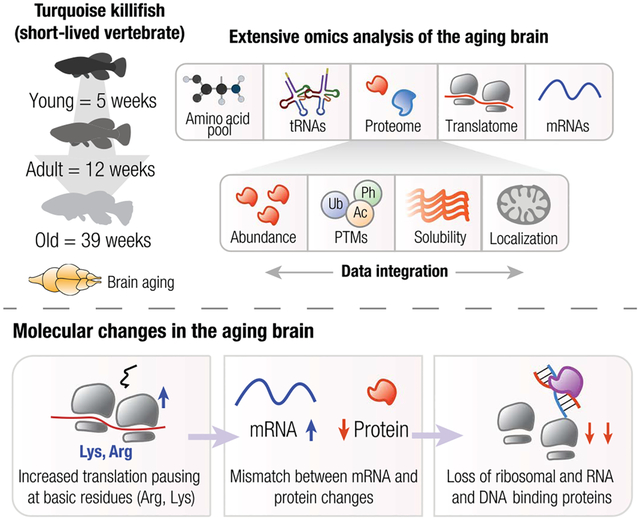

**Altered protein synthesis remodels the aging brain proteome.** Age-related mRNA and protein changes are decoupled. Ribosome profiling reveals increased translation elongation pausing, occurring preferentially at codons encoding for basic residues. Ribosome pausing contributes to protein-transcript decoupling and is associated with a depletion in RNA and DNA binding proteins. These changes also contribute to protein misfolding and insolubility, linking protein synthesis defects to other aging hallmarks. PTMs, posttranslational modifications.

Both aging and neurodegeneration disrupt protein homeostasis, also known as proteostasis, leading to the progressive accumulation of protein aggregates (1, 2). Proteostasis involves mechanisms that regulate the coordination of protein synthesis, degradation, and localization and is essential to ensure an adequate supply of functional proteins. It also prevents the accumulation of misfolded proteins and proteins not assembled in protein complexes that are more susceptible to aggregation.

Age-dependent decline in proteostasis coincides with the emergence of impairment in other aging hallmarks; however, an integrative analysis is needed to establish causality between proteostasis impairment and specific aging hallmarks. This knowledge gap persists, at least partially, because different proteostasis mechanisms have been analyzed separately and in different model systems. Therefore, we comprehensively analyzed proteostasis in the aging brain of the short-lived killifish, *Nothobranchius furzeri*. We focused on the brain because it comprises rarely dividing, largely postmitotic cells such as neurons, which are more susceptible to proteostasis impairment, a key pathophysiological mechanism in age-related human neurodegenerative disorders. We used killifish as a model organism because of its short life span and conserved aging brain hallmarks, such as gliosis, neuroinflammation, senescence, and protein aggregation as well as spontaneous emergence of neurodegenerative phenotypes (3–8).

We measured the effects of aging on mRNA and protein abundance as well as on protein posttranslational modifications, solubility, and subcellular localization, enabling quantification of their relation. We established a protocol for long-term partial inhibition of proteasome activity to investigate whether any age-related brain phenotype is induced by this specific dysfunction in vivo. Finally, we performed sequencing of ribosome-protected mRNA fragments (Ribo-seq) (9) to assess the contribution of mRNA translation to protein abundance changes. The biosynthesis of specific proteins rich in basic amino acids becomes impaired with age because of aberrant translation elongation and pausing at mRNA stretches encoding lysine and arginine. Such alterations lead to the depletion of subunits of protein complexes involved in DNA repair, transcription, splicing, and protein synthesis as well as changes in the proteome of mitochondria, all of which correspond to aging hallmarks. Altered translation dynamics provide a mechanistic explanation for the broadly conserved loss of correlation between transcriptome and proteome changes during aging (6, 10–15), which has also been linked to neurodegeneration in humans (16). Thus, our work reveals how altered biogenesis of a specific subset of proteins with age might further enhance vulnerabilities of the proteostasis network in the aging brain, contributing to the exacerbation of other aging hallmarks.

## Protein-transcript decoupling in the aging brain

**Fig. 1.**
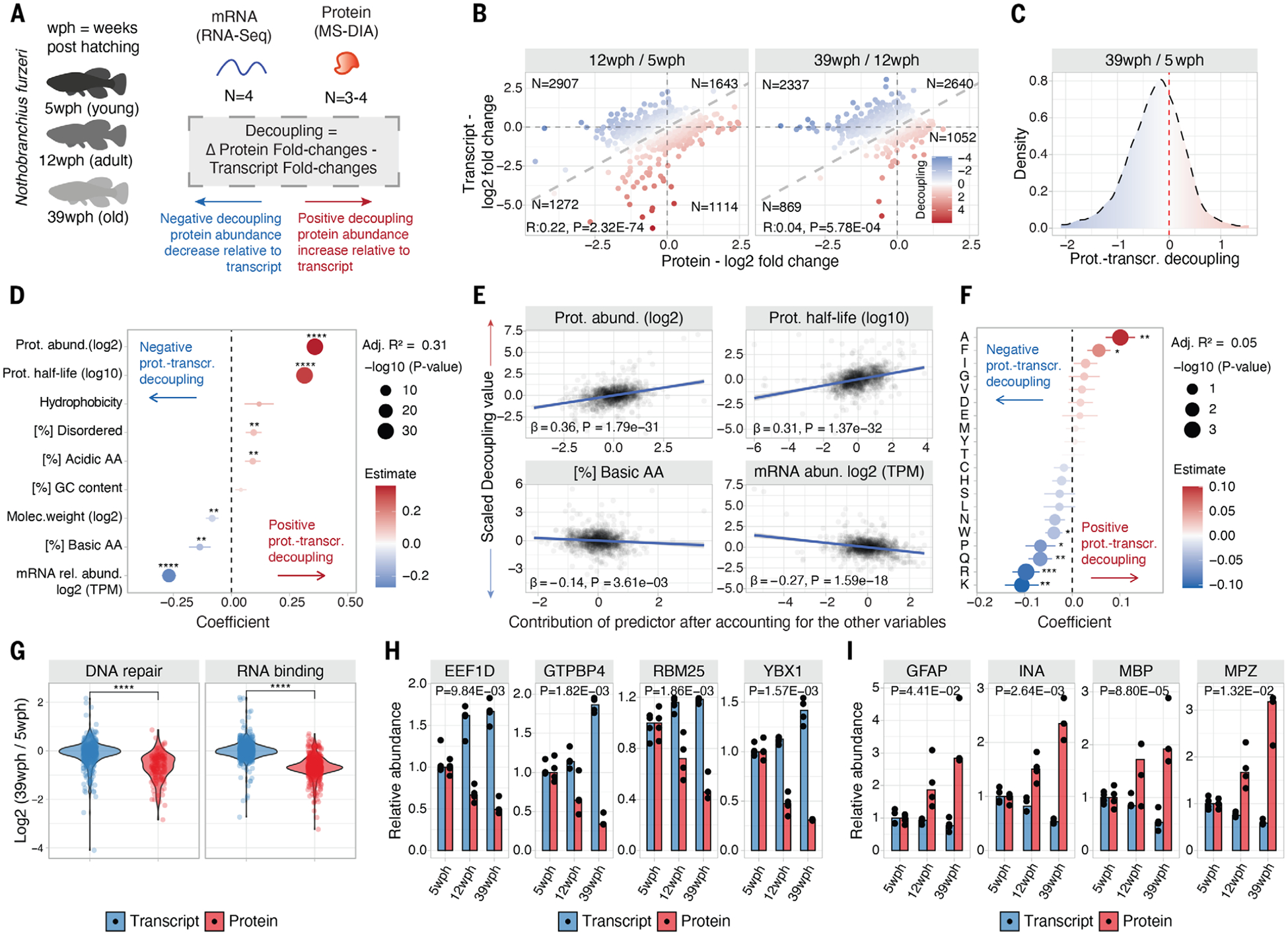
Changes in protein and mRNA abundance are decoupled in the aging brain. (**A**) Characterization of protein-transcript decoupling in the aging brain of killifish, age is reported as weeks post-hatching (wph). Positive decoupling values indicate increased protein abundance relative to transcripts, whereas negative decoupling indicates decreased protein abundance compared with transcripts. (**B**) Scatterplot comparing protein and transcript fold changes in the aging brain. Color represents decoupling score: red (blue) indicates increased (decreased) protein abundance relative to the transcript. Gray dashed lines indicate equal changes. Pearson’s correlation coefficient *r* was calculated between log_2_ transformed protein and transcript changes. *N* values indicate the number of transcript protein pairs in each of the quadrants. (**C**) Density distribution of decoupling scores for young versus old comparison. Red, positive decoupling; blue, negative decoupling. (**D**) Multiple linear regression analysis of decoupling scores based on transcript and protein features. F-test. (**E**) Added variable plot between features and decoupling scores. Protein half-life data were obtained from a mouse brain dataset (16), as no data of comparable depth are available for killifish. (**F**) Multiple linear regression analysis of decoupling scores based on protein amino acid composition. F-test. Single-letter abbreviations for the amino acid residues are as follows: A, Ala; C, Cys; D, Asp; E, Glu; F, Phe; G, Gly; H, His; I, Ile; K, Lys; L, Leu; M, Met; N, Asn; P, Pro; Q, Gln; R, Arg; S, Ser; T, Thr; V, Val; W, Trp; and Y, Tyr. (**G**) Transcript and protein fold changes for RNA binding and DNA repair proteins. Two-sample Wilcoxon test. (**H** and **I**) Examples of proteins with negative (H) and positive (I) decoupling (*N* = 3 or 4 biological replicates, MANOVA test). *P ≤ 0.05, **P ≤ 0.01, ***P ≤ 0.001, ****P ≤ 0.0001. Related to figs. S1 and S2 and table S1

To investigate the disruption of protein homeostasis in aging, we focused on the loss of correlation between changes in gene transcripts (mRNA) and corresponding protein, an evolutionarily conserved phenomenon (6, 10–15) here referred to as “decoupling,” for which a biological explanation is still lacking. To address the origin of decoupling, we combined RNA sequencing (RNA-seq) and mass spectrometry (MS)–based proteomics to quantify age-related changes in corresponding transcripts and protein abundance (Fig. 1, A and B, and fig. S1, A to H). We define “decoupling score” as the difference between protein and transcript changes between young and old samples (17). This decoupling score describes discrepancies between transcript and protein changes by identifying subsets of proteins displaying “positive protein-transcript decoupling,” meaning protein abundance greater than expected from changes of its corresponding transcript, or “negative protein-transcript decoupling,” meaning protein amounts lower than expected from changes of its corresponding transcript (Fig. 1B and table S1). The distribution of decoupling scores displayed a median shift toward negative values (Fig. 1C) driven by an overall skew toward decreased abundance of a subset of proteins (fig. S1D). To assess the reproducibility of this metric, we compared decoupling scores from this study with those obtained by analyzing an independent brain aging dataset (6). We detected a significant correlation of decoupling scores between the two datasets (fig. S1I), despite technical differences in the quantitative proteomics workflows: tandem mass tag (TMT)–based quantification (6) versus label-free data-independent acquisition (DIA, this study).

We applied multiple linear regression to investigate the association between decoupling scores (response variable, *N* = 1188 complete observations) and distinct biophysical properties of transcripts and proteins (*N* = 9 features). This model explained 31% of the decoupling variance (adjusted coefficient of determination *R*^2^ = 0.31; Fig. 1D). We detected estimated protein absolute abundance (see methods; β = 0.36, *P* < 2.20 × 10^−16^) as the parameter with the highest positive correlation with decoupling (Fig. 1E). To include protein half-life as a feature, we used data from the mouse brain (18), as such data are not available for killifish. Half-life provided the second highest positive partial correlation (β = 0.31, *P* < 2.20 × 10^−16^; Fig. 1E). On the other hand, the parameters with the highest negative correlation with decoupling were relative transcript abundance [expressed as log_2_ transcripts per million (TPM), β = −0.26, *P* < 2.20 × 10^−16^] and proportion of basic amino acids (β = −0.13, *P* = 4.30 × 10^−3^; Fig. 1, D and E). To dissect the contribution of individual amino acids to decoupling, we calculated a second regression model with protein amino acid composition as the sole predictor variable. There was a significant negative correlation between decoupling and the content of lysine, proline, glutamine, and arginine, whereas the neutral amino acids alanine and phenylalanine, which are enriched in abundant and hydrophobic proteins, positively correlated with decoupling (Fig. 1F).

We investigated whether decoupling affects proteins involved in DNA repair and RNA binding, given their higher content of basic amino acids relative to the rest of the proteome (fig. S1J). Both these groups of proteins showed an age-dependent decrease in protein but no change in transcript abundance (Fig. 1, G and H). On the other hand, myelin components, for example, myelin basic protein (MBP) and myelin protein P0 (MPZ), and intermediate filament proteins, such as glial fibrillary acidic protein (GFAP) and alpha-internecine (INA), characterized by long half-lives and low turnover rates [*Mus musculus* half-life: MBP, ~82 days; GFAP, ~13 days; INA, ~35 days (18, 19)] showed decreased transcript amounts but increased protein abundance with aging (Fig. 1I). We validated a subset of these age-related protein abundance changes using targeted mass spectrometry in an independent set of brain samples (fig. S1, K and L).

Because the brain of killifish undergoes changes in tissue weight and RNA content (fig. S2, A to C) and loss of neurons in specific brain regions with age (5), we repeated our analyses focusing on the optic tectum (fig. S2D and table S2), a brain region that does not display decreased neuron density with aging (4). To account for sex-dependent effects, we used tecta from male and female brains. The optic tectum analyses also revealed age-dependent protein-transcript decoupling of the same protein categories as in the whole brain (figs. S1, M and N, and S2E), independent of the animal’s sex (fig. S2E). Because previous studies identified organ-specific aging signatures (20, 21), we also investigated age-dependent decoupling in four other killifish organs: liver, muscle, heart, and fin (fig. S2F and table S3). Down-regulation and negative decoupling of proteins rich in basic amino acids were observed in muscle and fin and to a lesser extent in the heart but not in the liver (fig. S2, G and H).

Together, our data show that aging leads to sex-independent discrepancies between protein and transcript amounts for a subset of proteins in the aging vertebrate brain. Aging similarly affects the transcript-protein balance in other organs, with some interorgan variation.

## Convergence of proteome alterations on ribosomes and respiratory chain complexes

**Fig. 2.**
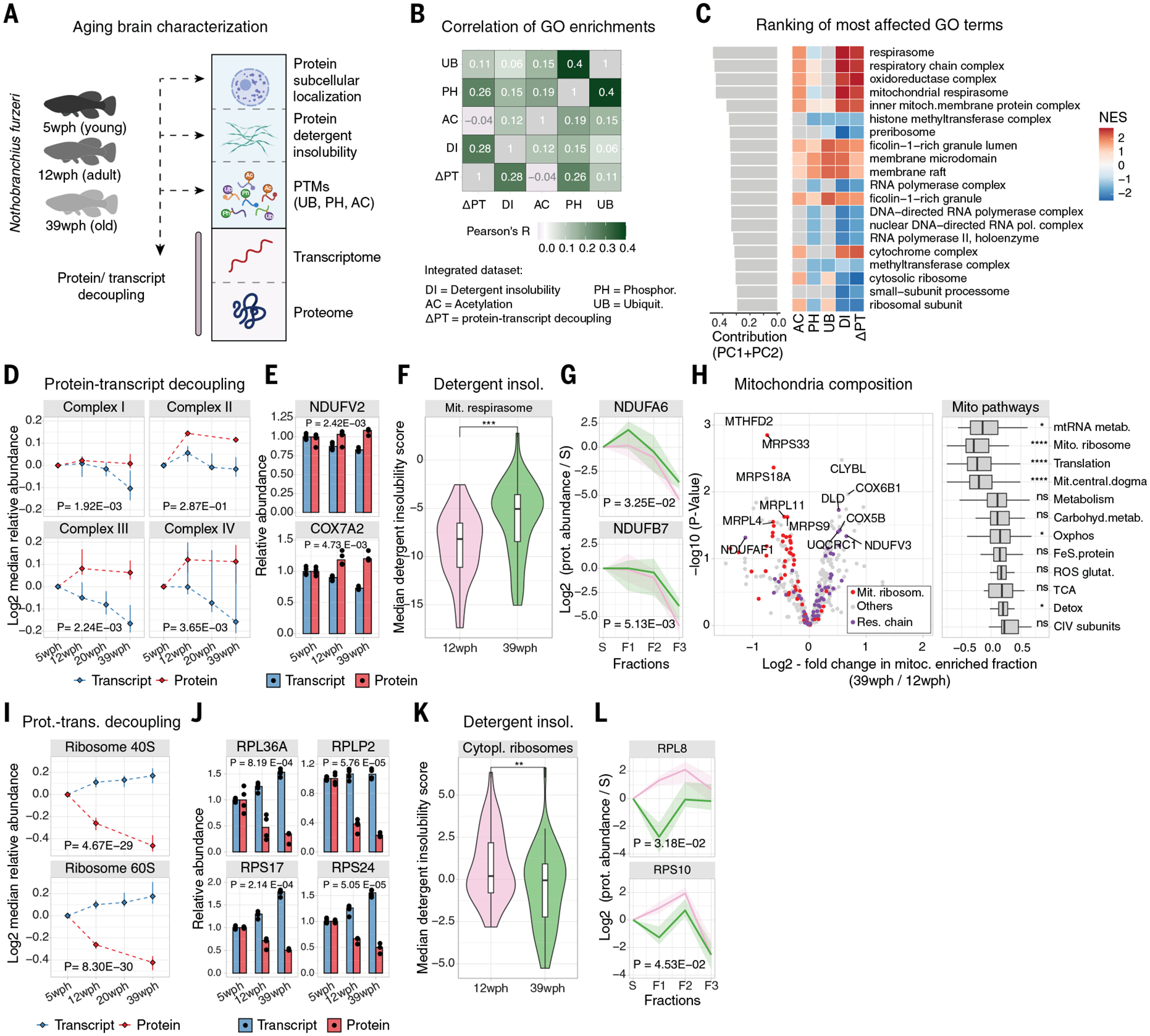
Age-related proteome changes converge on ribosomes and respiratory chain complexes. (**A**) Overview of the datasets generated in this study. (**B**) Heatmap showing correlations of NES across datasets (DI, detergent insolubility; ΔPT, protein-transcript decoupling; AC, acetylation; PH, phosphorylation; UB, ubiquitylation). (**C**) Top-ranking GO terms with strong contributions to PCA analysis. (**D**) Line plots for respiratory chain proteins’ transcript (blue) and protein (red) median abundance across age groups. Each point represents the median of the log_2_ ratio of the quantities relative to the young (5 wph) age group. Vertical lines indicate 50% of the distribution (*N* = 3 or 4 biological replicates, MANOVA test). (**E**) Examples of respiratory chain proteins with positive protein-transcript decoupling (*N* = 3 or 4 biological replicates, MANOVA test). (**F**) Violin plot showing detergent insolubility scores of mitochondrial respirasome proteins (*N* = 4 biological replicates, two-sample Wilcoxon test). (**G**) Examples of detergent insolubility profiles for respiratory chain proteins with increased detergent insolubility during aging (*N* = 4 biological replicates, MANOVA test). (**H**) Volcano plot showing changes in mitochondrial proteome due to aging. The box plot shows the effect of aging on different groups of mitochondrial pathways (*N* = 4 biological replicates, two-sample Wilcoxon test). (**I**) Ribosomal proteins’ transcript and protein abundance across age groups. Each point represents the median of the log_2_ ratio of the quantities relative to the young (5 wph) age group. Vertical lines indicate 50% of the distribution (*N* = 3 or 4 biological replicates, MANOVA test). (**J**) Examples of ribosomal proteins displaying negative protein-transcript decoupling (*N* = 3 or 4 biological replicates, MANOVA test). (**K**) Violin plot showing detergent insolubility scores of cytoplasmic ribosomal subunits (*N* = 4 biological replicates, two-sample Wilcoxon test). (**L**) Examples of detergent insolubility profiles for ribosomal proteins with decreased detergent insolubility during aging (*N* = 4 biological replicates, MANOVA test). *P ≤ 0.05, **P ≤ 0.01, ***P ≤ 0.001, ****P ≤ 0.0001. Related to figs. S3 to S8 and tables S1, S4, and S5.

We explored whether decoupling is linked to specific proteome alterations. To do so, we comprehensively characterized age-related changes in protein solubility by implementing a differential detergent extraction protocol (Fig. 2A, fig. S3, and table S4) (22) and organelle composition by subcellular fractionation in combination with mass spectrometry (23) (fig. S4 and table S4). Additionally, we quantified changes in phosphorylation, ubiquitylation, and acetylation to detect how these posttranslational modifications were affected in the aging brain (fig. S5 and table S5). We focused on these three classes of posttranslational modification because of their roles in neuronal physiology and plasticity (24–26) and because of the availability of protocols for enrichment of such modified peptides. The combined data provide an atlas of changes in organelle proteome composition, protein solubility, and posttranslational modifications in the aging brain (fig. S6) and highlight the impact of aging on a subset of proteins that have been genetically linked to neurodegeneration in humans (table S6 and fig. S7).

To explore the relation among different types of proteome alteration in the aging brain, we conducted a gene set enrichment analysis (GSEA) on the age-related proteome changes for each of the generated datasets (17). By calculating Pearson’s correlation coefficient (*r*) between enrichment scores across datasets, we found positive correlations between protein-transcript decoupling and increased detergent insolubility, a hallmark of protein aggregation (Pearson’s *r* = 0.28, *P* < 2.20 × 10^−16^), as well as protein phosphorylation (Pearson’s *r* = 0.26, *P* = 6.67 × 10^−8^), whereas other alterations, for instance, changes in protein ubiquitylation, showed smaller correlation values (Pearson’s *r* = 0.11, *P* = 1.23 × 10^−2^; Fig. 2B).

To unbiasedly identify the most prominently affected cellular components in our analysis, we used principal components analysis (PCA) to summarize the normalized enrichment scores (NES; fig. S8A). We ranked gene ontology (GO) terms by calculating the values of their projections on the first two principal component (PC) axes. The highest-ranking terms were related to mitochondrial respiratory chain components, ribosomes, and RNA polymerases (Fig. 2C). These sets of protein complexes were often affected by aging in opposite ways (Fig. 2C). Components of the respiratory chain showed a progressive decrease in their transcripts’ abundance and little or no increase in abundance of the corresponding proteins (Fig. 2, D and E, and fig. S8B). We validated this small increase using targeted mass spectrometry in an independent set of samples (fig. S8C). Respiratory chain proteins also showed an overall increase in detergent insolubility with aging, indicative of aggregation (Fig. 2, F and G). Notably, these alterations primarily affected respiratory chain components but not mitochondrial proteins in general (fig. S8D). To corroborate these findings, we examined our subcellular fractionation data (fig. S4). This analysis allowed us to identify two key aspects: (i) changes in the protein composition of aged mitochondria, particularly a significant decrease in the relative abundance of mitochondrial ribosomal proteins and an increase in the relative amount of oxidative phosphorylation (Fig. 2H and fig. S8D); and (ii) altered subcellular distribution of specific mitochondrial proteins (fig. S8, E and F). These analyses support a global remodeling of the mitochondrial proteome during aging.

In contrast to respiratory chain proteins, the abundance of both cytosolic and mitochondrial ribosomal proteins progressively decreased during aging (reaching, on average, an ~25% decrease in old brains), whereas the abundance of their corresponding transcripts increased (Fig. 2, I and J, and fig. S8, G and I). The depletion of selected ribosomal proteins was validated by targeted mass spectrometry and immunoblot (fig. S8, H to J, and fig. S11C). Depletion of ribosomal proteins and negative decoupling was observed during aging in all the organs we analyzed (fig. S8K). The same pattern was not observed for other genes modulated by the mechanistic target of rapamycin (mTOR) kinase signaling, such as genes carrying a 5′ terminal oligopyrimidine (TOP) motif (fig. S8L), suggesting that their decline is independent of major changes in mTOR activity. In the brain, the decreased abundance of ribosomal proteins was accompanied by decreased detergent insolubility (Fig. 2, K and L, and fig. S8, H and I). This alteration might be related to the loss of ribosome stoichiometry and partial assembly or disassembly (6). RNA polymerase II components showed similar protein abundance patterns and solubility changes (fig. S8M).

Together, these analyses show that aging preferentially affected proteins in the mitochondrial respiratory chain, ribosomes, and other DNA and RNA binding complexes in different ways. Most of these alterations occurred independently of mRNA changes, thus they may result from posttranscriptional mechanisms.

## Impact of proteasome inhibition on the brain proteome

**Fig. 3.**
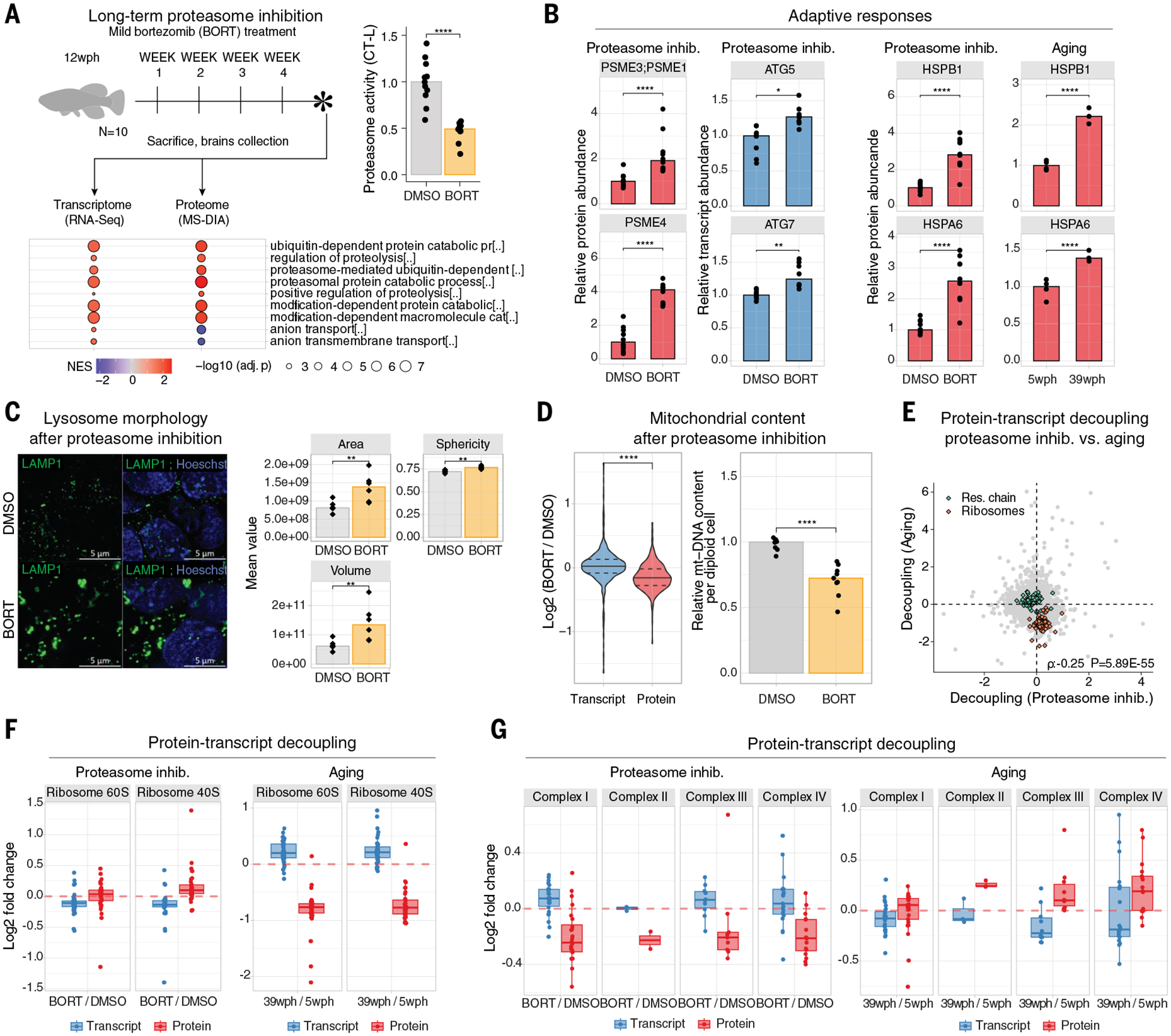
Effects of 4 weeks of in vivo proteasome inhibition on the adult killifish brain. (**A**) Adult killifish (12 wph) received weekly intraperitoneal injections of the proteasome inhibitor bortezomib or DMSO as vehicle control. (Bottom) GSEA color coded by NES. (Top right) Chymotrypsin-like proteasome activity quantification (*N* = 10 biological replicates, two-sample Wilcoxon test). (**B**) Barplot showing protein (red) and transcript (blue) abundance changes of selected proteins involved in proteostasis during aging and after proteasome inhibition. Asterisks indicate *Q* values from Spectronaut for proteins and adjusted *P* values from DESeq2 for transcripts (*N* = 10 biological replicates). (**C**) Effect of proteasome inhibition on lysosome morphology on the basis of lysosome-associated membrane protein 1 (LAMP1) immunofluorescence (left; scale bars = 5 μm) and quantification (right, *N* = 6 biological replicates, two-sample *t* test). (**D**) Effect of proteasome inhibition on mitochondrial transcripts and proteins (left, two-sample Wilcoxon test) and mtDNA (right, *N* = 10 biological replicates, two-sample Wilcoxon test). mtDNA copy number was calculated using real-time quantitative PCR with primers for 16*S* rRNA mitochondrial gene and cyclin-dependent kinase inhibitor 2a/b (Cdkn2a/b) nuclear gene for normalization. (**E**) Comparison of decoupling scores between aging and proteasome inhibition. Respiratory chain and ribosomal proteins are highlighted in green and orange, respectively. Spearman correlation was chosen because of the presence of outliers in the distribution. (**F** and **G**) Boxplots showing decoupling scores for ribosomal (F) and respiratory chain (G) proteins during aging and after proteasome inhibition. *P ≤ 0.05, **P ≤ 0.01, ***P ≤ 0.001, ****P ≤ 0.0001. Related to fig. S9 and table S7.

Protein degradation by the ubiquitin-proteasome system regulates protein abundance in organelles and complexes, including ribosomes and mitochondria. Aging is correlated with a decline in proteasome activity (2, 6, 27). To study the impact of proteasome activity on decoupling, we chronically inhibited its activity to ~50% in the brain of adult killifish over the course of 4 weeks and measured transcriptome and proteome (Fig. 3A and table S7). GO enrichment analysis revealed adaptive changes to proteasome inhibition such as overrepresentation of proteasome-related terms (Fig. 3A) and alterations of the proteostasis network (fig. S9A), including increased abundance of proteasome activators such as the proteasome activator complex subunit 1 (PSME1), also known as PA28ɑ, and key autophagy genes, for example, *ATG7* (Fig. 3B). Some protein abundance changes induced by proteasome inhibition, such as increased amounts of heat shock protein B1 (HSPB1), also occurred in aged killifish brains (Fig. 3B) and were independently confirmed by targeted mass spectrometry (fig. S9B). Proteasome inhibition led to morphological changes of lysosomes (Fig. 3C), which can also be observed in old brains (fig. S9C) and are linked to lysosomal storage disorders (28) and neurodegenerative diseases (29). As in aging, proteasome inhibition reduced mitochondrial content [estimated from the ratio of mitochondrial DNA (mtDNA) to nuclear DNA; Fig. 3D and fig. S9D] and globally decreased mitochondrial protein abundance independent of transcription (Fig. 3D) without altering abundance of master regulators of mitochondrial gene expression (fig. S9E).

Proteasome inhibition led to an increased abundance of shorter-lived proteins (fig. S9F) and to decoupling between transcript and protein changes (fig. S9G and table S7), consistent with its role in regulating protein turnover posttranscriptionally (30). However, when we applied the same linear regression models used for aging, we found that proteasome inhibition affected different sets of proteins with distinct biophysical properties than those exhibiting decoupling due to aging (fig. S9G). Specifically, proteasome inhibition caused an accumulation of proteins enriched in basic amino acids independently of transcription (fig. S9G). Indeed, the decoupling scores induced by proteasome inhibition and those observed in aging were negatively correlated (Spearman ρ = −0.25, *P* < 2.20 × 10^−16^; Fig. 3E). Proteins showing opposite decoupling between aging and proteasome inhibition included ribosomal proteins (Fig. 3F) and respiratory chain complexes (Fig. 3G). Comparable results were obtained when the same protocol of proteasome inhibition was administered to older fish (29 weeks after hatching) (fig. S9, H and I, and table S8). Thus, partial proteasome inhibition led to specific alterations in the adult killifish brain, some of which recapitulated aging brain phenotypes. However, decreased proteasome activity does not account for the loss of proteins enriched in basic amino acids observed in the old brains.

## Aberrant translation pausing correlates with decreased amounts of proteins enriched in basic amino acids

**Fig. 4.**
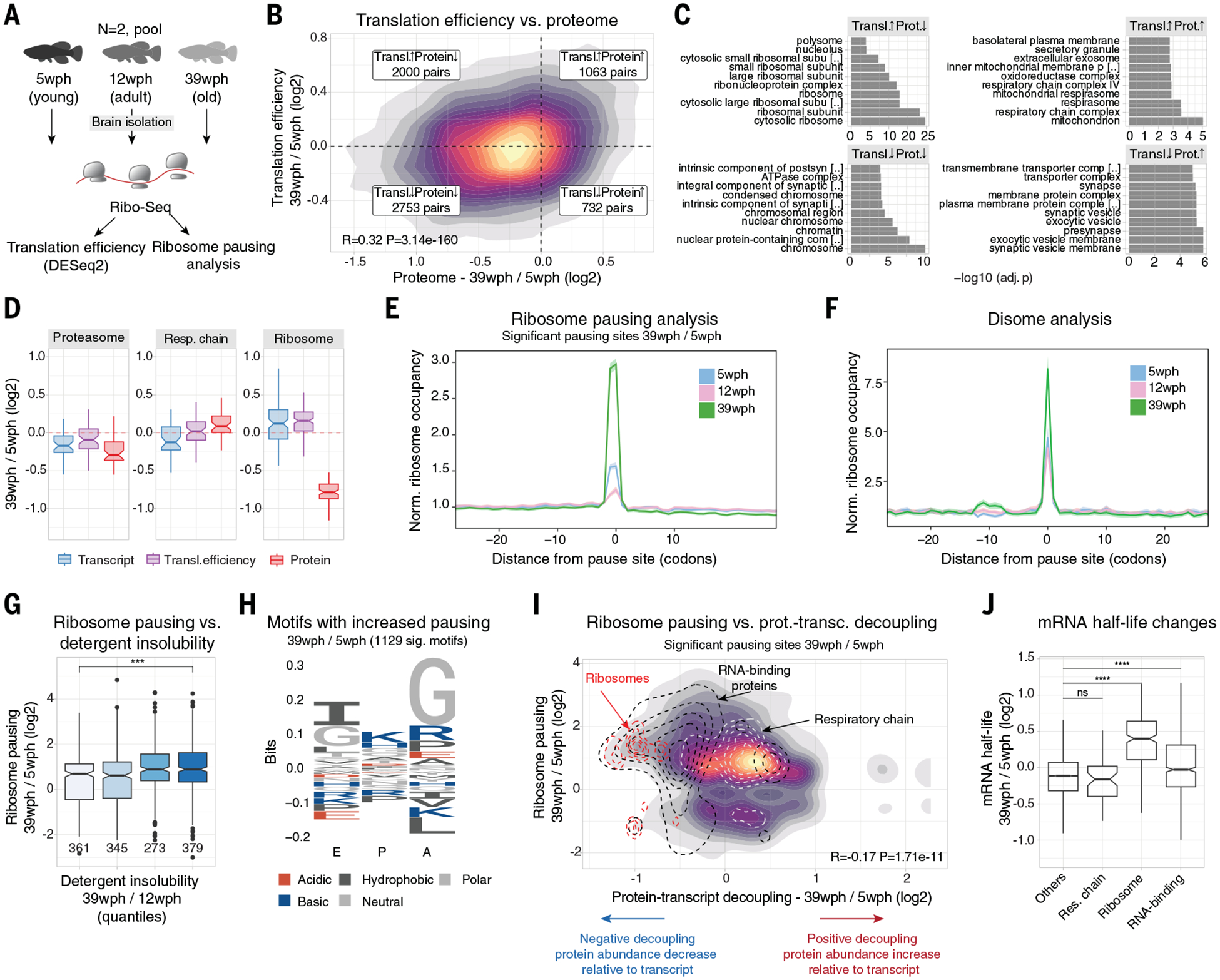
Increased translation pausing in the aging brain. (**A**) Ribosome profiling was conducted on brains of killifish of different ages. Each age group had two replicates consisting of independent pooled samples from 10 to 15 animals. (**B**) Two-dimensional (2D) density plot comparing age-related changes in protein abundance (*x* axis) and alterations in translation efficiency (*y* axis). Different quadrants highlight distinct modalities of translation regulation. (**C**) GO enrichment overrepresentation analysis for each quadrant of (B) (Fisher test with Holm correction). (**D**) Boxplots comparing age-related changes of mRNA abundance (blue), translation efficiency (purple), and protein abundance (red) for 26*S* proteasome, respiratory chain complexes, and cytoplasmic ribosomes. (**E**) Line plot showing the normalized ribosome distribution at pausing sites across different age groups. (**F**) Line plot depicting normalized disome ribosome distribution at disome pausing sites for various age groups. (**G**) Boxplot comparing ribosome pausing and age-related changes of protein solubility. Proteins encoded by transcripts displaying significant ribosome pausing in the old versus young comparison (adjusted *P* < 0.05) are grouped according to their age-related changes in detergent insolubility. The number of proteins in each quantile is indicated (two-sample Wilcoxon test). (**H**) Logo plot of amino acids being decoded at age-dependent increased pausing sites (pause score at 39 wph > pause score at 5 and 12 wph, and pause score at 39 wph > 6). E, tRNA exit site; P, peptidyl-tRNA binding site; A, aminoacyl-tRNA binding site. (**I**) 2D density plot showing the relation between significant pausing changes (adjusted *P* < 0.05) and decoupling. Contours: cytoplasmic ribosomes (red), RNA binding proteins (black), and oxidative phosphorylation (white). (**J**) Boxplot showing estimated mRNA half-life changes with age for selected gene sets (two-sample Wilcoxon test with Holm correction). *P ≤ 0.05, **P ≤ 0.01, ***P ≤ 0.001, ****P ≤ 0.0001. Related to figs. S10 and 11 and table S9.

Our findings show that imbalances in proteostasis during aging do not arise solely from proteasome dysfunction. Other factors, such as differential mRNA translation in old age, could cause the observed discrepancies between transcript and protein abundance. Therefore, we conducted a Ribo-seq experiment in aging killifish brains (Fig. 4A and table S9). Trinucleotide periodicity and consistent replicates (fig. S10, A and B) demonstrated the overall quality of the data, whereas comparison between mRNA abundance and ribosome occupancy showed expected correlations (*r* = 0.25, *P* < 2.20 × 10^−16^; fig. S10C). Translation efficiency (TE) analysis (17) revealed that TE exhibits a higher correlation with protein changes (*r* = 0.32, *P* < 2.20 × 10^−16^; Fig. 4B) than with transcript changes (*r* = 0.23, *P* < 2.20 × 10^−16^), consistent with observations in mammals (31). For instance, shifts in TE led to consistent changes in protein abundance for certain protein complexes, such as complex IV of the respiratory chain and the 26*S* proteasome (Fig. 4, C and D, and fig. S10D). However, TE did not explain the decreased proteins of ribosomes, RNA polymerase II, and other nucleic acid–binding proteins linked to DNA repair during aging (Fig. 4, C and D, and fig. S10D). This analysis excludes TE as the cause of the decreased abundance of these proteins in aging brains.

We drew inspiration from studies on aged nematodes and yeasts, which exhibit age-related impaired translation elongation and increased ribosome pausing (32). We searched our Ribo-seq data for signatures of translation pausing (17), revealing an overall increase in site-specific pausing in the aging brain (Fig. 4E and table S9). Disome analysis confirmed an increase in ribosome collisions in the aged brain (Fig. 4F). Age-linked increased pausing was also observed in the liver, albeit to a lower extent than in the brain (fig. S11A).

Elongation stalling leads to ubiquitylation of specific subunits of the 40*S* ribosome by the ribosome quality control (RQC) machinery (33–35). Indeed, anisomycin-induced ribosome stalling in killifish cells induced a characteristic higher molecular weight ubiquitylated band in immunoblots of 40*S* subunit RPS3 (fig. S11B), commonly associated with ribosome stalling (33–35). Analysis of aged brains showed similarly increased ubiquitylation in RPS3 (fig. S11C), even though ubiquitylation of most ribosomal proteins decreased with age (fig. S11D). Additionally, we noted an aging-dependent increase of ATF3 mRNA (fig. S11E), a transcription factor whose abundance increases upon sensing of ribosome collisions as part of the ribosome stress response (36, 37). As observed in aged yeast and nematodes, we also observed a decrease in the levels of a subset of proteins involved in RQC (fig. S11F). This RQC reduction may exacerbate ribosome collisions and stalling as age progresses, potentially slowing stalled mRNA degradation and causing their accumulation in aging cells.

We investigated the impact of translation pausing on cellular proteostasis. We observed a positive association between transcripts subject to increased translation pausing with age and increased age-dependent detergent insolubility of their encoded polypeptide, indicative of aggregation (Fig. 4G). As these alterations affected key proteostasis network components such as the proteasome (fig. S11G), the increase in elongation pausing and associated aggregation may create a vicious cycle of proteostasis collapse. Stretches enriched in codons for basic residues (arginine and lysine) as well as glycine were enriched at sites of aging-linked elongation pausing (Fig. 4H) and disome forming sites (fig. S11H). Our decoupling model linked enrichment in these same residues (arginine and lysine) to reduced protein abundance with age (Fig. 1F). Furthermore, we found a small but statistically significant correlation between increased elongation pausing and protein-transcript decoupling (Fig. 4I; *r* = −0.17, *P* < 2.20 × 10^−16^), which explained changes in ribosomal and RNA binding proteins, where protein decline did not follow transcript changes. In contrast, pausing in transcripts encoding components of the respiratory chain did not deviate from the overall pausing distribution (Fig. 4I).

Changes in translation and pausing affect mRNA half-life (38–40). Therefore, we investigated the association between translation pausing and computationally estimated mRNA half-life in our RNA-seq data (17, 41). Transcripts encoding ribosomal proteins and RNA binding proteins show an increased half-life in old brains compared with the rest of the transcriptome (Fig. 4J). Thus, alterations in translation might influence other aspects of the aging brain transcriptome, such as the half-life of specific mRNAs. These analyses show that increased ribosome occupancy does not necessarily result in enhanced protein synthesis in the aging brain. Our comprehensive analysis of transcription, translation, protein abundance, and protein solubility in the aging brain indicates that translation dysfunction, and specifically aberrant elongation pausing, may underlie the decreased abundance of ribosomal proteins and other nucleic acid–binding proteins in the aging brain.

## Effect of aging on the abundance of amino acids and tRNA charging

**Fig. 5.**
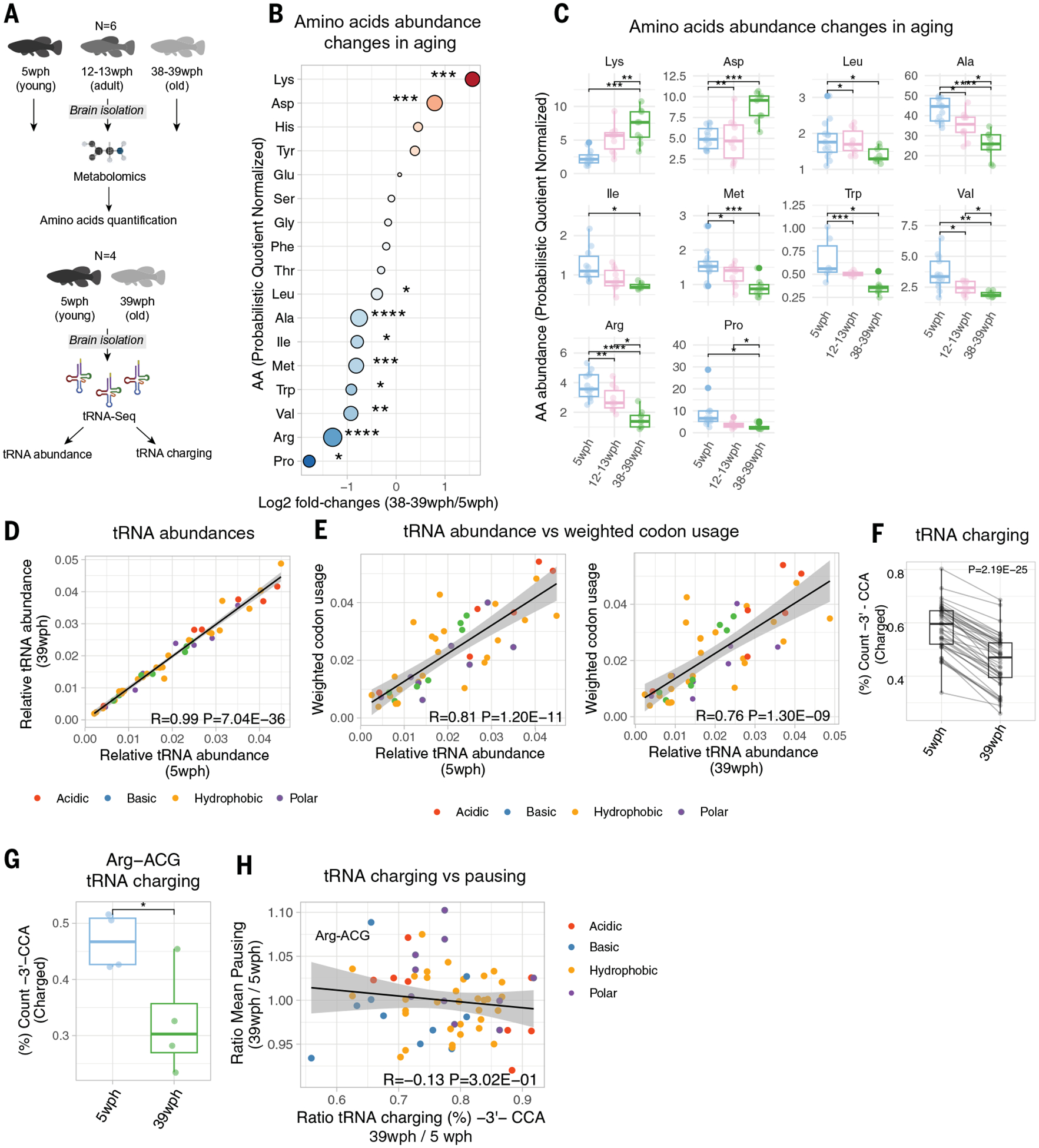
Changes in amino acid abundance and tRNA charging in the aging brain. (**A**) Metabolomics and tRNA-seq were conducted on brains of killifish of different ages. (**B**) Dot plot showing changes in amino acid abundance in the aging killifish brain (*N* = 6 biological replicates, two-sample *t* test with Welch’s correction). (**C**) Boxplot displaying the quantification of amino acids that show significant (*P* ≤ 0.05) age-related changes (*N* = 6 biological replicates, two-sample *t* test with Welch’s correction). Amino acid abundances were obtained using tandem mass spectrometry (MS/MS), and samples were normalized through probabilistic quotient normalization. (**D**) Scatterplots showing the difference in relative abundance levels of tRNAs in young and old killifish. (**E**) Scatterplot comparing relative tRNA abundances and weighted codon usage (codon frequency normalized by transcript abundance) in young (left panel) and old (right panel) fish. (**F**) Boxplot showing the percentage of 3′-CCA counts for each tRNA, indicating the percentage of charged tRNAs in each sample (*N* = 4 biological replicates, paired *t* test). (**G**) Boxplot showing changes in charging for the tRNA Arg-ACG (*N* = 4 biological replicates, two-sample *t* test with Welch’s correction). (**H**) Scatterplot showing the correlation between age-dependent changes in average pausing at each codon and changes in tRNA charging for the corresponding anticodon. Dots are colored according to the different amino acid types that the tRNAs encode. *P ≤ 0.05, **P ≤ 0.01, ***P ≤ 0.001, ****P ≤ 0.0001. Related to fig. S12 and tables S10 and S11.

To understand how age-related translation pausing occurs, we examined how metabolic changes affect abundance of certain amino acids and whether aging influences the amounts and charging of tRNAs, as these factors could cause codon-specific alterations in translation supply for the ribosomes. We measured the abundance of amino acids, total and charged tRNA pools, and the protein levels of tRNA synthetases (Fig. 5A and tables S10 and S11). Amounts of multiple amino acids were altered in the aged brains, with arginine decreased by one-half (Fig. 5, B and C, and fig. S12A). tRNA amounts correlated with weighted codon usage in both young and old fish brains (Fig. 5D), and their total abundance was not affected by aging (Fig. 5E). Of note, the tRNA charging state was globally decreased with aging across all the tRNAs irrespective of the amino acid they carry (Fig. 5F). Abundance of multiple tRNA synthetases, including arginine tRNA synthetase (RARS), was decreased in the old brains (fig. S12B). Given that the changed amounts of charged tRNAs with aging do not mirror the changes in amino acid pools, decreased abundance of tRNA synthetases might explain decreased tRNA charging in old brains. We investigated the relation between changes in tRNA charging and the age-related increase in translation pausing. This analysis revealed that changes in tRNA charging were not correlated with increased translation pausing (*r* = −0.13, *P* = 0.3; Fig. 5H), even though some codons clearly showed both increased pausing and decreased charging of their corresponding tRNA, for example, Arg-ACG (Fig. 5, G and H). Together, these data show that aging affects the abundance of amino acids and tRNA charging in the brain. Decreased tRNA charge may contribute to, but does not fully explain, the increased ribosome pausing observed in old brains.

## A possible model for protein biosynthesis in the aging brain

We observed alterations in protein synthesis in old brains, leading to decreased abundance of ribosomal proteins, among other RNA binding proteins. The reduced abundance of ribosomal proteins may reflect lower numbers of ribosomes in old brains. To confirm that aging decreases the amounts of fully assembled and translating ribosomes, we isolated polysome-containing fractions from the brains of young and old fish on sucrose gradients and quantified ribosomal proteins by mass spectrometry. We confirmed a reduced abundance of ribosomal proteins in polysome fractions relative to that of monosomes (80*S*) in old brains (fig. S13).

**Fig. 6.**
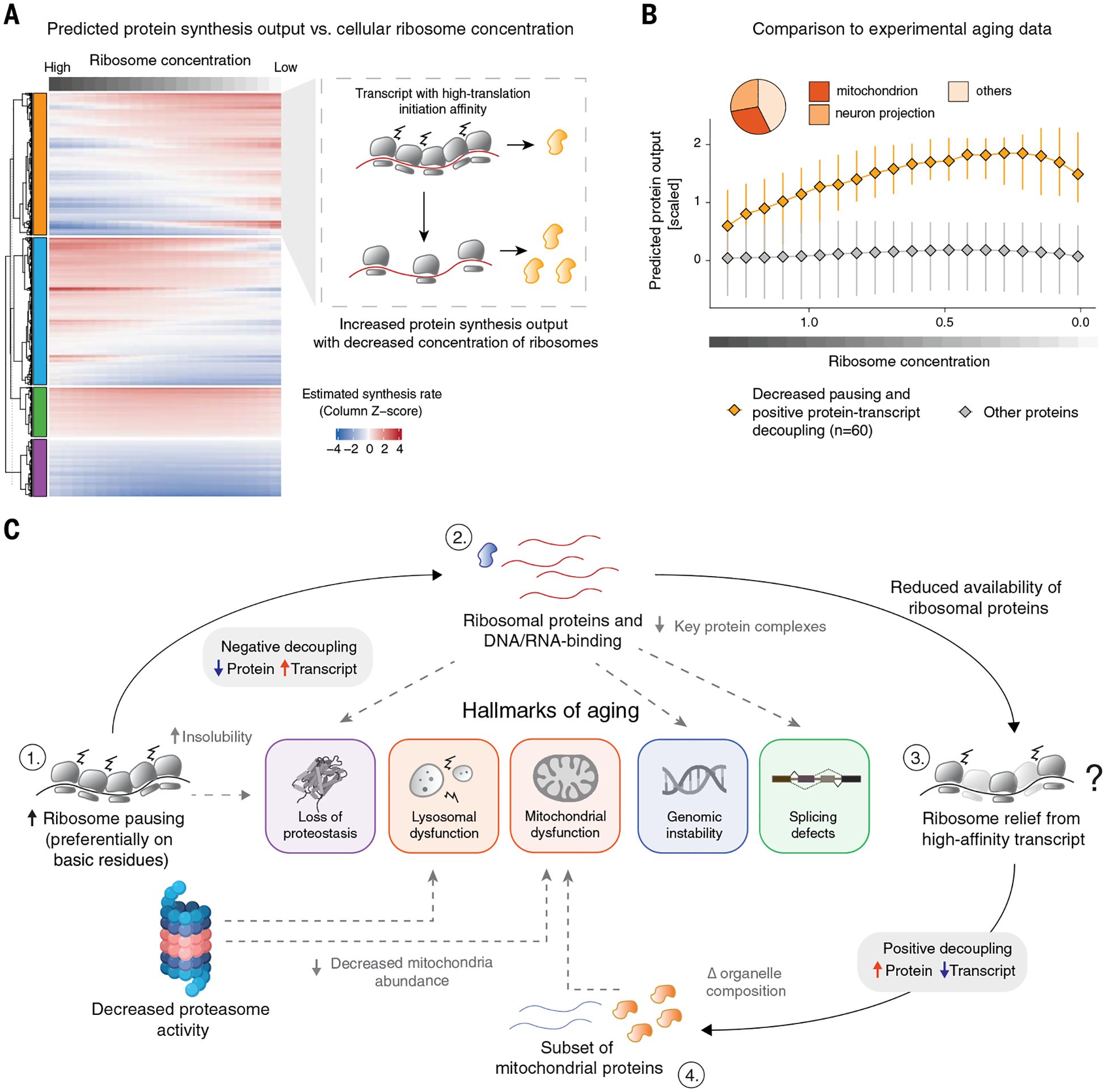
Decreased abundance of ribosomal proteins might lead to translation changes in the aging brain. (**A**) Heatmap showing the estimated output of protein synthesis in relation to changes of ribosome concentration, modeled as described by Mills and Green (42) and Khajuria (43). Columns indicate the estimated protein output for a specific ribosome concentration. Transcripts (rows) are clustered with a hierarchical clustering using the “ward D2” algorithm on the dissimilarity (1–Pearson’s correlation) measure. For display purposes, the heatmap shows 5000 randomly sampled transcripts. The right panel shows an illustrative example of a cluster displaying increased estimated protein output as a function of reduced ribosome abundance levels. For these transcripts, an overall decrease of ribosome concentration is predicted to reduce ribosome collisions and pausing on the mRNA, leading to overall increased protein synthesis output. (**B**) Line plot showing the estimated protein synthesis output for transcript displaying decreased ribosome pausing in the killifish brain Ribo-seq data (median per transcript log_2_ pausing 39 wph versus 5 wph < 0 and adjusted *P* ≤ 0.15) and positive decoupling with aging (increased protein abundance levels relative to the transcript, orange). The *x* axis represents the simulated decreased ribosomal concentration, while the *y* axis indicates the estimated protein output, as shown also in (A). (**C**) Schematic representation of the contribution of altered translation elongation and proteasome inhibition to other hallmarks of aging. An increase in translation elongation pausing occurs during brain aging in killifish correlating with a reduced synthesis of a subset of proteins enriched in basic amino acids, including ribosomal proteins. The decreased availability of ribosomal proteins and the ensuing reduction of translating ribosomes might favor the translation of mRNAs with high translation initiation affinity, for example, a subset of respiratory chain components, leading to increased abundance of the corresponding proteins in the aging brain. Proteasome inhibition affected aging hallmarks distinct from those linked to translation dysfunction and primarily affected lysosome morphology and mitochondrial abundance. Related to fig. S13 and table S12.

Altered ribosome concentration can directly affect the translation of specific mRNAs, as observed in a group of inherited diseases collectively referred to as ribosomopathies (42, 43). Protein output of specific mRNAs has been predicted to be influenced by ribosome availability depending on transcript-specific translation initiation rate *k*_*i*_ (where *k*_*i*_ refers to the affinity of specific mRNA sequences to bind ribosomes) (17, 42, 43). Under these assumptions, a decrease in ribosome concentration might, for example, increase protein synthesis from transcripts with a high translation initiation rate by lowering the total number of ribosomes on them, relieving trafficking and pausing events (Fig. 6A). To test this hypothesis in the context of an aging brain, we estimated *k*_*i*_ from killifish 5′ untranslated region (5′UTR) sequences on the basis of experimental data (44) and modeled the estimated synthesis rate as described in (17, 42, 43) (Fig. 6A and table S12). A subset of killifish transcripts had increased predicted synthesis rates as a function of decreased ribosome concentration (Fig. 6A, orange cluster, and table S12). In our experimental data, we selected a specific set of proteins showing decreased translation pausing and increased protein abundance in our decoupling model (60 proteins; Fig. 4I, bottom right quadrant). We estimated their predicted synthesis rates as a function of ribosome concentration. Consistent with the experimental data, the relative synthesis of this subset of proteins was predicted to increase after a reduction of ribosome concentration (Fig. 6B). Approximately one-third of these proteins were mitochondrial (including seven components of the respiratory chain), and another prominent fraction belonged to proteins related to neuron projections (Fig. 6B). The absence of ribosomal proteins in this subset, despite their high *k*_*i*_ value, indicates distinct translation dynamics for these proteins, possibly resulting from their increased elongation pausing during aging. We propose that the decreased abundance of ribosomal proteins in the aged brain, possibly triggered by aberrant pausing events, might reduce the availability of translating ribosomes and, thereby, alter abundance of a subset of the proteome independently of transcript abundance (Fig. 6C).

## Discussion

Our study offers insight into how distinct proteostasis mechanisms reshape the vertebrate brain proteome during aging. We show proteome changes in aging brains, encompassing protein synthesis, solubility, posttranslational modifications, subcellular localization, and organelle composition. Among these, we show that abundance of ribosomal subunits and DNA or RNA binding proteins decrease independently of mRNA abundance. We propose that translation alterations, including elongation pausing at sequences enriched in basic residues, are central drivers of these changes, leading to discrepancies between mRNA concentrations, ribosome occupancy, and protein synthesis. We identified changes in protein ubiquitylation in ribosomal proteins that have been associated with ribosome collision induced by different types of translation or proteotoxic stress (34, 35), supporting an age-dependent increase in elongation pausing. Consistent with these findings, we also observed an age-related increase in the mRNA abundance of the transcription factor ATF3, indicative of activation of the ribosomal stress response pathway (36, 37).

At least two key implications emerge from our findings. First, aging leads to decreased abundance of protein complexes containing polybasic stretches, including ribosomes, spliceosomes, RNA and DNA polymerases, and those functioning in DNA repair. This phenomenon, which correlates with the content of basic amino acids in their protein sequences and consequently with their likelihood of being affected by ribosome stalling (45) (Fig. 4H and figs. S1J and S11H), may affect multiple steps of the gene expression process and could mechanistically place proteostasis impairment upstream of other canonical aging hallmarks, such as DNA damage, epigenetic alterations (46), aberrant splicing (47), and reduced RNA polymerase activity (48) (Fig. 6C).

A second implication is that aging leads to altered mitochondrial composition. These changes encompass a decrease in abundance of mitochondrial ribosomal proteins and possibly mitochondrial translation, whereas respiratory chain components remain stable or increase in abundance. This is consistent with broader observations of aging-induced mitochondrial changes (49, 50). These findings based on bulk tissue measurements were corroborated by more direct analysis of the composition of mitochondria from subcellular fractions and by other age-dependent alterations of mitochondrial proteins, for example, changes in detergent insolubility. Decreased mtDNA content appears to be induced by decreased proteasome activity, showcasing the convergence of different proteostasis mechanisms affecting critical cellular structures during aging.

The mechanisms leading to increased translation pausing remain unclear. Our analysis indicates enhanced age-dependent pausing at polybasic enriched sequences, which are known to slow down translation (45, 51–53). These sequences might represent the first ones to reveal impaired elongation when the availability of key factors required for protein synthesis become more scarce in aged cells. In old brains, we found alterations in amino acid abundances and tRNA charging as well as levels of key RQC factors that might contribute, at least in part, to the observed increase in elongation pausing. Additional mechanisms contributing to increased translational pausing could result from the decreased abundance of adenosine triphosphate (ATP) observed in old tissues (54–57). Decreased energy might alter the decoding kinetics for specific nonoptimal codons, such as those encoding basic amino acids (58, 59), leading to a decreased synthesis rate for these proteins. It remains unclear whether these signatures are a cause or consequence of increased ribosome pausing in the aging brain.

Decoupling in aging manifests as a decrease in protein abundance of ribosomal proteins together with a progressive increase in transcript amounts, not only in the brain but also in the fin, heart, liver, and muscle. These findings are consistent with observations from various species. An age-dependent increase of transcripts encoding for ribosomal proteins was observed by single-cell RNA-seq in multiple cell types of the murine brain (60). Accordingly, increased transcripts encoding for ribosomal proteins were among the most consistent transcriptional signatures of longevity shared across multiple tissues and mammalian species (61). Our results indicate that this increase might result not from increased transcription but rather from increased mRNA stability. Decreased abundance of ribosomal proteins with age has been described in multiple organs in mice (62) as well as in nematodes (63), and the protein half-life of ribosomes is affected by aging in the mouse brain (64). These data identify reduced content of ribosomal proteins as a hallmark of aging in vertebrates. We found decreased abundance of DNA and RNA binding proteins occurring independently of mRNA abundance—decoupling—in other killifish organs including fin and muscle but not in the liver. Aged livers had less translation pausing and decrease of basic protein concentration than did the brain. This points to tissue-specific differences in protein synthesis regulation during aging, consistent with observations in rodents (31). In mice, different organs age at distinct rates (20, 65). Relevant to our findings, the brain is especially sensitive to aging, with an overwhelming number of late-onset neurodegenerative diseases affecting the brain.

The proteome composition in the aging brain may be influenced by additional processes. For instance, age-dependent impairment of protein degradation by the autophagy-lysosome system can lead to the accumulation of specific proteins (66), as has been shown for MBP in mouse microglia (67). Consistently, we observed accumulation of MBP and other myelin proteins independent of mRNA changes. A similar impairment of protein clearance might also occur in the aging killifish brain. Stalling of RNA polymerase II occurs with aging, thereby skewing the transcription output in a gene length–dependent manner (48), consistent with a systemic loss of long transcripts observed in multiple aging tissues and species (68). A reduction in the abundance of specific transcripts could increase transcriptional noise, leading to an imbalance in the stoichiometry of protein complexes, overwhelming proteostasis and altering the relationship between mRNA and protein abundance, especially for long-lived proteins. Aging may also affect posttranscriptional RNA modification (epitranscriptomics), which influences RNA stability and translation in different contexts, including aging (69, 70). It is conceivable that mRNA modifications might contribute to protein-transcript decoupling. Finally, we have identified multiple alterations of protein posttranslational modification, most notably phosphorylation. Some of these have been linked to protein aggregation in human neurodegenerative disorders, for example, hyperphosphorylation of microtubule-associated protein tau (17).

Our work contributes to advancing our understanding of the relationship between aging and the risk of neurodegenerative diseases. We provide a resource (accessible at https://genome.leibniz-fli.de/shiny/orilab/notho-brain-atlas/) of proteome alterations in the aging killifish brain and show that multiple proteins and signaling pathways associated with neurodegeneration in humans become perturbed in various ways during physiological aging in killifish (17) (figs. S6 and S7). Such alterations might underlie convergent mechanisms between aging and mutations that increase the risk of neurodegeneration in aged individuals. Translation pausing might represent one of such converging pathophysiological mechanisms, given that ribosome stalling is correlated with proteostasis perturbation in various neurodegenerative diseases (57–60).

## Materials and methods

### Animal management practices

All experiments were performed using the killifish strain MZM-0410 (unless otherwise stated) in accordance with relevant guidelines and regulations. Fish were bred and kept in FLI’s fish facility according to §11 of the German Animal Welfare Act under license number J-003798. The animal experiment protocols were approved by the local authority in the State of Thuringia (Veterinaer-und Lebensmittelueberwachungsamt; proteasome inhibition: reference number 22-2684-04-FLI-19-010). Sacrifice and organ harvesting of nonexperimental animals were performed according to §4(3) of the German Animal Welfare Act.

Fish were kept in recirculating fish racks (Aqua Schwarz, Göttingen, Germany) with central filtration individually or in groups (maximum one fish per 1.7 liters) with 12:12-hour light:dark cycle. Water temperature (27° ± 1°C) conductivity (~2.5 mS) and pH were monitored and recorded continuously. Group-housed fish received certified contaminant-screened blue polycarbonate igloos, retreats, huts, and tents (Bio-Serv, Flemington) as enrichment. Breeding groups, which consisted of one male and four females, were provided with a sand bowl for oviposition. Embryos were collected on coconut fiber substrate (Dragon Coco-Ground, Zoo Zajac, Duisburg, Germany) and incubated at 23°C. Freshly hatched larvae were fed twice daily with live first instar brine shrimp nauplii (*Artemia* sp.) and then weaned at the age of 4 to 6 weeks onto live bloodworms (Chironomidae) once a day. Trained caretakers assessed fish condition daily on the basis of FLI killifish health score sheet (71), and fish were euthanized by rapid chilling if one of the endpoints of the severity assessment was reached. Health monitoring followed the protocol described in (71).

### In vivo proteasome inhibition

Adult [12 to 14 weeks post-hatching (wph)] and old (29 wph) killifish were subjected to pharmacological intervention via intraperitoneal injections during 4 weeks of treatment. Male and female fish with a sex ratio 1:1 were used. Trained caretakers assessed fish condition daily on the basis of FLI killifish health score sheet (71), and fish were euthanized by rapid chilling if one of the endpoints of the severity assessment was reached. One fish was excluded from the study. Every 6 days starting on day 0 and ending with day 24 (*t* = 0, 6, 12, 18, and 24), fish were anesthetized with 200 mg/liter buffered MS-222 (PharmaQ) and gently manipulated to deliver intraperitoneal injection of bortezomib at 500 μM or vehicle [1% dimethyl sulfoxide (DMSO) in a physiological salt solution] at a dosage of 10 μl/g body weight. Animals from the same hatch were randomly allocated to the experimental groups. Both male and female fish were included in each experimental group. Individual brains from the fish were collected on the last day of treatment and snap-frozen in liquid nitrogen.

### Proteasome activity assay

CT-L (chymotrypsin-like) proteasome activity was assayed with the hydrolysis of a specific fluorogenic substrate, Suc-LLVY-AMC (UBPBio, Catalog Number G1100). On the day of the experiment, brains were lysed in buffer [50 mM HEPES, pH 7.5 (Sigma Aldrich, H3375); 5 mM EDTA (Carl Roth, 8043.2); 150 mM NaCl (Carl Roth, 3957.1); 1% (v/v) Triton X-100 (Carl Roth, 3051.3); 2 mM ATP (Sigma Aldrich, A2383) prepared with Milli-Q water] to a final estimated protein concentration of ~4 mg/ml and homogenized by sonication (Bioruptor Plus) for 10 cycles (30 s ON/60 s OFF) at high setting, at 4°C. Lysates corresponding to 10 μg protein were incubated in 50 mM Tris-HCl, pH 7.4, 5 mM MgCl_2_, 1 mM ATP, 1 mM dithiothreitol (DTT), 10% glycerol, and 10 μM proteasome substrate for 1 hour at 37°C. Specific proteasome activity was determined as the difference between the total activity of protein extracts and the remaining activity in the presence of 20 μM MG132 (Enzo Life Sciences, BML-PI102-0005). Fluorescence was measured by multiple reads for 60 min at 37°C by TECAN Kinetic Analysis (excitation 380 nm, emission 460 nm, read interval 5 min) on a Safire II microplate reader (TECAN).

### Sample preparation for total proteome and analysis of posttranslational modifications (PTMs)

Snap-frozen brains from pools of male and female killifish with a sex ratio of 1:1 were thawed and transferred into Precellys lysing kit tubes [Keramik-kit 1.4/2.8 mm, 2 ml (CKM)] containing 150 μl of phosphate-buffered saline (PBS) supplemented with cOmplete, Mini, EDTA-free protease inhibitor (Roche,11836170001) and with PhosSTOP Phosphatase Inhibitor (Roche, 4906837001). On the basis of estimated protein content (5% of fresh tissue weight), three to six brains were pooled to obtain ~1.5 mg of protein extract as starting material for each biological replicate, maintaining a sex ratio between males and females of 1:1. Tissues were homogenized twice at 6000 rpm for 30 s using Precellys 24 Dual (Bertin Instruments, Montigny-le-Bretonneux, France). The homogenates were transferred to new 2 ml Eppendorf tubes. Proteins were quantified using Pierce BCA Protein Assay Kit (Thermo Scientific, 23225), and 1.25 mg was processed for further analysis. Volumes were adjusted using PBS, and one-fourth of the volume equivalent of the 4× lysis (8% SDS, 100 mM HEPES, pH 8) buffer was added. Samples were sonicated twice in a Bioruptor Plus for 10 cycles with 1 min ON and 30 s OFF with high intensity at 20°C. The lysates were centrifuged at 18,407*g* for 1 min and transferred to new 1.5 ml Eppendorf tubes. Subsequently, samples were reduced using 10 mM DTT (Carl Roth, 6908) for 15 min at 45°C and alkylated using freshly made 200 mM iodoacetamide (IAA) (Sigma-Aldrich, I1149) for 30 min at room temperature in the dark. An aliquot of each lysate was used to estimate the precise protein quantity using BCA (Thermo Scientific, 23225). Subsequently, proteins were precipitated using cold acetone, as described in (72), and resuspended in 500 μl of digestion buffer (3 M urea, 100 mM HEPES pH 8.0). Aliquots corresponding to 20, 200, and 1000 μg protein were taken for proteome, phosphopeptides, and ubiquitylated/acetylated peptides enrichment, respectively, and digested using LysC 1:100 enzyme: proteins ratio for 4 hours (Wako sequencing grade, 125-05061) and trypsin 1:100 enzyme: proteins ratio for 16 hours (Promega sequencing grade, V5111). The digested proteins were then acidified with 10% (v/v) trifluoroacetic acid (TFA) and desalted using Waters Oasis HLB μElution Plate 30 μm (2, 10, and 30 mg, depending on the amount of starting material) following manufacturer’s instructions. The eluates were dried down using a vacuum concentrator and reconstituted in MS buffer A (5% (v/v) acetonitrile, 0.1% (v/v) formic acid). For PTM enrichment, peptides were further processed as described below. For DIA-based analysis of total proteome, samples were transferred to MS vials, diluted to a concentration of 1 μg/μl, and spiked with iRT kit peptides (Biognosys, K_i_-3002-2) before analysis by liquid chromatography–tandem mass spectrometry (LC-MS/MS).

### Sequential enrichment of ubiquitylated and acetylated peptides

Ubiquitylated and acetylated peptides were sequentially enriched, starting from ~1000 μg of dried peptides per replicate. For the enrichment of ubiquitylated peptides, the PTMScan HS Ubiquitin/SUMO Remnant Motif (K-ε-GG) kit (Cell Signaling Technology, 59322) was used following manufacturer’s instructions. The K-ε-GG modified enriched fraction was desalted and concentrated as described above, dissolved in MS buffer A, and spiked with iRT kit peptides before LC-MS/MS analysis.

The flowthrough fractions from the K-ε-GG enrichment were acidified with 10% (v/v) trifluoroacetic acid and desalted using Oasis HLB μElution Plate 30 μm (30 mg) following manufacturer’s instructions. Acetylated peptides were enriched as described by (73). Briefly, dried peptides were dissolved in 1000 μl of intraperitoneal buffer (50 mM MOPS pH 7.3, 10 mM KPO_4_ pH 7.5, 50 mM NaCl, 2.5 mM octyl β-d-glucopyranoside) to reach a peptide concentration of 1 μg/μl, followed by sonication in a Bioruptor Plus (five cycles with 1 min ON and 30 s OFF with high intensity at 20°C). Agarose beads coupled to an antibody against acetyl-lysine (ImmuneChem Pharmaceuticals Inc., ICP0388–5MG) were washed three times with washing buffer (20 mM MOPS pH 7.4, 10 mM KPO_4_ pH 7.5, 50 mM NaCl) before incubation with each peptide sample for 1.5 hour on a rotating well at 750 rpm (STARLAB Tube roller Mixer RM Multi-1). Samples were transferred into Clearspin filter microtubes (0.22 μm) (Dominique Dutscher SAS, Brumath, 007857ACL) and centrifuged at 4°C for 1 min at 2000*g*. Beads were washed first with intraperitoneal buffer (three times), then with washing buffer (three times), and finally with 5 mM ammonium bicarbonate (three times). Thereupon, the enriched peptides were eluted first in basic condition using 50 mM aqueous NH_3_, then using 0.1% (v/v) trifluoroacetic acid in 10% (v/v) 2-propanol and finally with 0.1% (v/v) trifluoroacetic acid. Elutions were dried down and reconstituted in MS buffer A (5% (v/v) acetonitrile, 0.1% (v/v) formic acid), acidified with 10% (v/v) trifluoroacetic acid, and then desalted with Oasis HLB μElution Plate 30 μm. Desalted peptides were finally dissolved in MS buffer A, spiked with iRT kit peptides, and analyzed by LC-MS/MS.

### Enrichment of phosphorylated peptides

Lysates (corresponding to ~200 μg of protein extract) were acetone precipitated, digested into peptides, and desalted, as described in “Sample preparation for total proteome and analysis of posttranslational modifications.” The last desalting step was performed using 50 μl of 80% acetonitrile and 0.1% TFA buffer solution. Before phosphopeptide enrichment, samples were filled up to 210 μl using 80% acetonitrile and 0.1% TFA buffer solution. Phosphorylated peptides were enriched using Fe(III)-NTA cartridges (Agilent Technologies, G5496-60085) in an automated fashion using the standard protocol from the AssayMAP Bravo Platform (Agilent Technologies). In short, Fe(III)-NTA cartridges were first primed with 100 μl of priming buffer (100% acetonitrile, 0.1% TFA) and equilibrated with 50 μl of buffer solution (80% acetonitrile, 0.1% TFA). After loading the samples into the cartridge, the cartridges were washed with an OASIS elution buffer, while the syringes were washed with a priming buffer (100% acetonitrile, 0.1% TFA). The phosphopeptides were eluted with 25 μl of 1% ammonia directly into 25 μl of 10% formic acid. Samples were dried down with a speed vacuum centrifuge and stored at −20°C until LC-MS/MS analysis.

### Subcellular fraction of killifish brain by LOPIT-DC

All the following steps were performed at 4°C, keeping samples on ice unless stated otherwise. Fresh brains from adult (12 wph) and old (39 wph) killifish were pooled to reach ~150 mg of wet tissue weight per biological replicate (roughly 20 animals per biological replicate). Male and female fish with a sex ratio 1:1 were used. Fresh brain tissue was subsequently transferred to a 15 ml Potter homogenizer (Fisher Scientific, 15351321) together with 7.5 ml of lysis buffer (LB) (250 mM sucrose, 10 mM HEPES pH 8.0, 2 mM MgAc, 2 mM EDTA) supplemented with protease inhibitor (Roche,11836170001) and homogenized with ~60 gentle strokes. The brain homogenate was then transferred in a 15 ml Falcon tube and treated with Benzonase (Merk, 70664) for 20 min at room temperature. An aliquot of 2.5 ml homogenate was collected for each sample and stored at −80°C to be later processed for differential detergent extraction (see below). The remaining 5 ml were transferred to a 5 ml Eppendorf tube and centrifuged at 500*g* for 5 min at 4°C to remove cell debris and unlysed cells. Subsequently, the clarified homogenate was centrifuged at 1000*g* for 13 min at 4°C, and the resulting pellet was collected as the first subcellular fraction (01). Following one additional centrifugation at 1000*g* for 7 min, the supernatant was then divided into 4 × 1.5 ml Ultracentrifuge Tubes (Beckman) and processed for differential ultracentrifugation step with an Optima TLX-BenchTop Ultracentrifuge (Beckman, 8043-30-1197), using a TLA55 rotor (Beckman, 366725), using the ultracentrifugation settings shown in Table 1.

Pellets from each centrifugation step were resuspended in 50 μl of PBS, and proteins were solubilized by adding 50 μl of 2x lysis buffer [200 mM HEPES pH 8.0, 100 mM DTT, 4% (w/v) SDS]. For fraction 10 (cytosol enriched), 300 μl was taken and supplemented with 300 μl of 2x lysis buffer. All the samples were then sonicated using a Bioruptor Plus (Diagenode) for five cycles with 60 s ON and 30 s OFF with max intensity, boiled for 10 min at 95°C, and a second sonication cycle was performed. The solubilized proteins were reduced with 200 mM DTT for 15 min at 45°C and alkylated using freshly made 200 mM IAA for 30 min at room temperature in the dark. Subsequently, proteins were precipitated using cold acetone, dissolved in 1 M guanidine HCl in 100 mM HEPES pH 8.0, and digested using LysC and trypsin, as described in (72). The digested proteins were then acidified with 10% (v/v) trifluoroacetic acid and desalted using Oasis HLB μElution Plate 30 μm following manufacturer’s instructions. The eluates were dried down using a vacuum concentrator and reconstituted in 5% (v/v) acetonitrile, 0.1% (v/v) formic acid. Samples were transferred directly to MS vials, diluted to a concentration of ~1 μg/μl, and spiked with iRT kit peptides before analysis by LC-MS/MS.

### Differential detergent extraction

All the following steps were performed at 4°C, keeping samples on ice unless stated otherwise. For each replicate, 2.5 ml of brain homogenate (see LOPIT-DC protocol above) was thawed on ice. After thawing, the homogenate was centrifuged at 500*g* for 5 min at 4°C to remove debris. The supernatant was collected, and 64 μl of 20% (v/v) IGEPAL Nonidet P-40 (Sigma) was added to reach an initial concentration of 0.5% (v/v). The homogenate was then divided into 4x 1.5 ml ultracentrifuge tubes and sonicated in a Bioruptor Plus for 10 cycles with 30 min ON and 30 s OFF with max intensity at 24°C. The homogenates were then loaded into a TLA55 rotor and ultracentrifuged with an Optima TLX-BenchTop Ultracentrifuge at 100,000*g* for 5 min at 24°C. After ultracentrifugation, the supernatants were collected and stored as a “soluble” (S) fraction. The remaining pellets were resuspended in 1 ml of buffer A (10 mM HEPES pH 8.0, 2 mM MgAc, 2 mM EDTA, 0.5% NP-40), samples were mixed by vortexing, and sonicated in a Bioruptor Plus for 10 cycles with 30 s ON and 30 s OFF with max intensity at 24°C. Samples were then ultracentrifuged again at 100,000*g* for 5 min at 24°C. The supernatants (“F1”) were collected, and the remaining pellets were resuspended in 1 ml of buffer B (10 mM HEPES pH 8.0, 2 mM MgAc, 2mM EDTA, 0.5% NP-40, 0.25% SDS, 0.5% deoxycholic acid), mixed, sonicated, and centrifuged as above. The supernatants (“F2”) were collected, and the remaining pellets were resuspended in 1 ml of buffer C (10 mM HEPES pH 8.0, 2 mM MgAc, 2mM EDTA, 0.5% NP-40, 2% SDS, 0.5% deoxycholic acid), mixed, sonicated, and centrifuged as above. The supernatants (“F3”) and the remaining pellets were collected. All the collected samples were stored at −80°C until further analysis.

### Data-independent acquisition for proteome quantification

Peptides were separated in trap/elute mode using the nanoAcquity MClass Ultra-High Performance Liquid Chromatography system (Waters, Waters Corporation, Milford, MA, USA) equipped with trapping (nanoAcquity Symmetry C18, 5 μm, 180 μm × 20 mm) and an analytical column (nanoAcquity BEH C18, 1.7 μm, 75 μm × 250 mm). Solvent A was water and 0.1% formic acid, and solvent B was acetonitrile and 0.1% formic acid. One microliter of the samples (~1 μg on column) was loaded with a constant flow of solvent A at 5 μl/min onto the trapping column. Trapping time was 6 min. Peptides were eluted via the analytical column with a constant flow of 0.3 μl/min. During the elution, the percentage of solvent B increased nonlinearly from 0 to 40% in 120 min. The total run time was 145 min, including equilibration and conditioning. The LC was coupled to an Orbitrap Exploris 480 (Thermo Fisher Scientific, Bremen, Germany) using the Proxeon nanospray source. The peptides were introduced into the mass spectrometer via a Pico-Tip Emitter 360-μm outer diameter × 20-μm inner diameter, 10-μm tip (New Objective) heated at 300°C, and a spray voltage of 2.2 kV was applied. The capillary temperature was set at 300°C. The radio frequency ion funnel was set to 30%. For DIA data acquisition, full scan mass spectrometry (MS) spectra with a mass range 350–1650 mass/charge ratio (*m*/*z*) were acquired in profile mode in the Orbitrap with a resolution of 120,000 FWHM (full width at half maximum). The default charge state was set to 3+. The filling time was set at a maximum of 60 ms with a limitation of 3 × 10^6^ ions. DIA scans were acquired with 40 mass window segments of differing widths across the MS1 mass range. Higher collisional dissociation fragmentation (stepped normalized collision energy; 25, 27.5, and 30%) was applied, and MS/MS spectra were acquired with a resolution of 30,000 FWHM with a fixed first mass of 200 *m*/*z* after accumulation of 3 × 10^6^ ions or after filling time of 35 ms (whichever occurred first). Data were acquired in profile mode. For data acquisition and processing of the raw data, Xcalibur 4.3 (Thermo) and Tune version 2.0 were used.

### Data processing for MS-DIA samples

Spectral libraries were created by searching the DIA or/and DDA runs using Spectronaut Pulsar (14.9.2 and 15.3.2, Biognosys, Zurich, Switzerland). The data were searched against species-specific protein databases (Nfu_20150522, annotation nfurzeri_genebuild_v1.150922) with a list of common contaminants appended. The data were searched with the following modifications: carbamidomethyl (C) as fixed modification, and oxidation (M), acetyl (protein N-term), lysine di-glycine (K-ε-GG), phosphorylated tyrosine (T) and serine (S) and acetyl-lysine (K-Ac) as variable modifications for the respective enrichments. A maximum of three missed cleavages were allowed for K-Ac and K-ε-GG modifications, two missed cleavages were allowed for phospho enrichment. The library search was set to 1% false discovery rate (FDR) at both protein and peptide levels. DIA data were then uploaded and searched against this spectral library using Spectronaut Professional (v14.9.2 and 15.3.2) and default settings. Relative quantification was performed in Spectronaut for each pairwise comparison using the replicate samples from each condition using default settings, except the ones displayed in Table 2.

Tests for differential abundance were performed using an unpaired *t* test between replicates. *P* values were corrected for multiple testing using the method described by Storey (74) to obtain FDR adjusted *P* values (*Q* values). Candidates and report tables were exported from Spectronaut and used for downstream analysis.

### TMT labeling

Tandem mass tags (TMT)–based protein quantification was used for killifish heart proteome data. The solution containing the resuspended peptides was brought to a pH of 8.5 and a final concentration of 100 mM HEPES (Sigma H3375) before labeling. Twenty micrograms of peptides was used for each label reaction. TMT-10plex reagents (Thermo Fisher #90111) were reconstituted in 41 μl of acetonitrile (Biosolve #0001204102BS). TMT labeling was performed in two steps by addition of 2x of the TMT reagent per milligram of peptide (e.g., 40 μg of TMT reagent for 20 μg of peptides). TMT reagents were added to samples at room temperature, followed by incubation in a thermomixer (Eppendorf) under constant shaking at 600 rpm for 30 min. After incubation, a second portion of TMT reagent was added and followed by incubation for another 30 min. After checking the labeling efficiency by MS, equal amounts of samples were pooled (200 μg total), desalted using two wells of a Waters Oasis HLB mElution Plate 30 mm (Waters #186001828BA), and subjected to high pH fractionation before MS analysis.

### High pH peptide fractionation

Offline high pH reverse phase fractionation was performed using an Agilent 1260 Infinity HPLC System equipped with a binary pump, degasser, variable wavelength UV detector (set to 220 and 254 nm), Peltier-cooled autosampler (set at 10°C) and a fraction collector. The column used was a Waters XBridge C18 column (3.5 mm, 100 3 1.0 mm, Waters) with a Gemini C18, 4 3 2.0 mm SecurityGuard (Phenomenex) cartridge as a guard column. The solvent system consisted of 20 mM ammonium formate [20 mM formic acid (Biosolve #00069141A8BS), 20 mM (Fluka #9857) pH 10.0] as mobile phase (A) and 100% acetonitrile (Biosolve #0001204102BS) as mobile phase (B). The separation was performed at a mobile phase flow rate of 0.1 ml/min using a nonlinear gradient from 95% A to 40% B for 91 min. Forty-eight fractions were collected along with the LC separation and subsequently dried in a speed vacuum centrifuge and then stored at −80°C until MS analysis.

### Data acquisition for TMT-labeled samples

For TMT experiments, fractions were resuspended in 20 μl reconstitution buffer [5% (v/v) acetonitrile (Biosolve #0001204102BS), 0.1% (v/v) TFA in water] and 5 μl were injected into the mass spectrometer. Peptides were separated using the nanoAcquity UPLC system (Waters) fitted with a trapping (nanoAcquity Symmetry C18, 5 μm, 180 μm × 20 mm) and an analytical column (nanoAcquity BEH C18, 2.5 μm, 75 μm × 250 mm). The analytical column outlet was coupled directly to an Orbitrap Fusion Lumos (Thermo Fisher Scientific) using the Proxeon nanospray source. Solvent A was water with 0.1% (v/v) formic acid and solvent B was acetonitrile, 0.1% (v/v) formic acid. The samples were loaded with a constant flow of solvent A at 5 μl/min, onto the trapping column. Trapping time was 6 min. Peptides were eluted via the analytical column at a constant flow rate of 0.3 μl/min, at 40°C. During the elution step, the percentage of solvent B increased in a linear fashion from 5% to 7% in the first 10 min, then from 7% B to 30% B in the following 105 min and to 45% B by 130 min. The peptides were introduced into the mass spectrometer via a Pico-Tip Emitter 360 μm OD x 20 μm ID; 10 mm tip (New Objective) and a spray voltage of 2.2 kV was applied. The capillary temperature was set at 300°C. Full scan MS spectra with a mass range of 375–1500 *m*/*z* were acquired in profile mode in the Orbitrap with a resolution of 60,000 FWHM using the quad isolation. The RF on the ion funnel was set to 40%. The filling time was set to a maximum of 100 ms with an automatic gain control (AGC) target of 4 × 10^5^ ions and one microscan. The peptide monoisotopic precursor selection was enabled along with relaxed restrictions if too few precursors were found. The most intense ions (instrument operated for a 3 s cycle time) from the full scan MS were selected for MS2, using quadrupole isolation and a window of 1 Da. HCD was performed with a collision energy of 35%. A maximum fill time of 50 ms for each precursor ion was set. MS2 data were acquired with a fixed first mass of 120 *m*/*z* and acquired in the ion trap in Rapid scan mode. The dynamic exclusion list was set with a maximum retention period of 60 s and a relative mass window of 10 parts per million (ppm). For the MS3, the precursor selection window was set to the range 400–2000 *m*/*z*, with an exclusion width of 18 *m*/*z* (high) and 5 *m*/*z* (low). The most intense fragments from the MS2 experiment were co-isolated (using Synchronous Precursor Selection = 8) and fragmented using HCD (65%). MS3 spectra were acquired in the Orbitrap over the mass range of 100–1000 *m*/*z*, and the resolution was set to 30,000 FWHM. The maximum injection time was set to 105 ms, and the instrument was set to inject ions for all available parallelizable times. The Xcalibur v4.0 and Tune v2.1 were used to acquire and process raw data.

### Data processing for TMT labeled samples

TMT-10plex data were processed using ProteomeDiscoverer v2.0 (Thermo Fisher). Data were searched against the relevant species-specific fasta database (Nfu_20150522, annotation nfurzeri_genebuild_v1.150922) using Mascot v2.5.1 (Matrix Science) with the following settings: Enzyme was set to trypsin, with up to one missed cleavage. MS1 mass tolerance was set to 10 ppm and MS2 to 0.5 Da. Carbamidomethyl cysteine was set as a fixed modification, and oxidation of methionine as variable. Other modifications included the TMT-10plex modification from the quantification method used. The quantification method was set for reporter ions quantification with HCD and MS3 (mass tolerance, 10 ppm). The FDR for peptide-spectrum matches (PSMs) was set to 0.01 using Percolator (75). Reporter ion intensity values for the PSMs were exported and processed with procedures written in R, as described in (76). Briefly, PSMs mapping to reverse or contaminant hits, or having a Mascot score below 15, or having reporter ion intensities below 1 × 10^3^ in all the relevant TMT channels were discarded. TMT channels intensities from the retained PSMs were then log_2_ transformed, normalized, and summarized into protein group quantities by taking the median value. At least two unique peptides per protein were required for the identification, and only those peptides with one missing value across all 10 channels were considered for quantification. Protein differential expression was evaluated using the limma package (77). Differences in protein abundances were statistically determined using an unpaired Student’s *t* test moderated by the empirical Bayes method. *P* values were adjusted for multiple testing using the Benjamini-Hochberg method (FDR, denoted as “adj.p”) (78).

### Parallel reaction monitoring (PRM)

For each protein target, at least two peptides were selected among the most confident and consistently identified peptides from the DIA analysis, and their isotopically labeled version [peptide C-terminal heavy arginine (U-13C6; U-15N4) or lysine (U-13C6; U-15N2)] synthesized by JPT Peptide Technologies GmbH (Berlin, Germany). Lyophilized peptides were delivered and reconstituted in 20% (v/v) acetonitrile, 0.1% (v/v) formic acid and further pooled together in equimolar ratios. An aliquot of the pooled peptides, corresponding to ~150 fmol per peptide, was analyzed by both DDA and DIA LC-MS/MS and used for assay generation using Spectrodive (Biognosys AG, Schlieren, Switzerland).

For PRM measurements, digested peptides from brain samples were spiked with synthetic heavy labeled peptides at a concentration of 10 fmol per 2.5 μg of peptides. Male and female fish with a sex ratio 1:1 were used. Peptides were separated using a nanoAcquity UPLC M-Class system (Waters, Milford, MA, USA) with a trapping (nanoAcquity Symmetry C18, 5 μm, 180 μm × 20 mm) and an analytical column (nanoAcquity BEH C18, 1.7 μm, 75 μm × 250 mm). The analytical column outlet was coupled directly to an Orbitrap Fusion Lumos (Thermo Fisher Scientific, Waltham, MA, USA) using the Proxeon nanospray source. Solvent A was water, 0.1% (v/v) formic acid, and solvent B was acetonitrile, 0.1% (v/v) formic acid. Peptides were eluted via the analytical column with a constant flow of 0.3 μl/min. During the elution step, the percentage of solvent B increased in a nonlinear fashion from 0% to 40% in 40 min. The total runtime was 60 min, including cleanup and column re-equilibration. PRM acquisition was performed in a scheduled fashion for the duration of the entire gradient using the “tMSn” mode with the following settings: resolution 120,000 FWHM, AGC target 3 × 10^6^, maximum injection time (IT) 350 ms, isolation window 0.4 *m*/*z*. For each cycle, a “full MS” scan was acquired with the following settings: resolution 120,000 FWHM, AGC target 3 × 10^6^, maximum injection time (IT) 10 ms, scan range 350 to 1650 *m*/*z*. Peak group identification was performed using SpectroDive and manually reviewed. Quantification was performed using a spike-in approach using the ratio between endogenous (light) and reference (heavy) peptides.

### Amino acid quantification

Brain tissue was collected from storage at −80°C and moved to dry ice during the extraction procedure. Male and female fish with a sex ratio 1:1 were used. At first, 75 μl of extraction solvent (80% MeOH, −80°C, 5 μM internal standard caffeine) were added to the frozen tissue. The extraction mix was vortexed thoroughly (1 min), followed by brief sonication (10 s, 0°C), and then vortexed again (1 min). The mixture was briefly spun down (2 s) to ensure all biomaterial was in the extraction solvent at the bottom of the tube and left for further extraction and protein precipitation overnight at −80°C. After 1 hour of storage, samples were vortexed briefly, followed by another short spin down (2 s). The next day, all samples were vortexed briefly, then precipitates were removed via centrifugation (5 min, 15,000*g*, 4°C). To ensure all particles and precipitate were removed, 70 μl of clear supernatant were collected, transferred into a fresh, precooled tube, and stored in the freezer (−80°C, 1 hour) followed by centrifugation (5 min, 15,000*g*, 4°C). Subsequently, 40 μl of clear supernatant was collected and subjected to targeted metabolomic analysis by LC-MS/MS. LC-MS/MS analysis was based on a method modified from (79). Samples were injected without further dilution. Chromatographic separation was carried out using an Agilent 1290 Infinity II UHPLC system equipped with a Waters Acquity BEH Amide column (1.7 μm, 2.1 mm × 100 mm). The column temperature was set to 35°C, and 0.3 μl of sample were injected into a gradient with a flow rate of 0.9 ml/min. Mobile phase A consisted of 1:1 (v:v) acetonitrile:water, 10 mM ammonium formate, 0.176% formic acid, and Mobile phase B consisted of 95:5:5 (v:v:v) acetonitrile:methanol:water, 10 mM ammonium formate, 0.176% formic acid. The chromatographic gradient was as follows: 0 min, 85% B; 0.7 min, 85% B; 2.55 min, 5% B; 2.9 min, 5% B; 2.91 min, 85% B; 3.5 min, stop. MS-based detection and quantification were achieved using an Agilent triple quadrupole (QQQ) 6470 mass spectrometer equipped with a JetStream ion source (AJS-ESI). Data acquisition was performed in dynamic (scheduled) MRM mode to monitor analyte-specific precursor product ion transitions. The cycle time was 320 ms. MS-Source parameters were as follows: gas temperature 325°C, gas flow 10 liters/min, nebulizer gas 40 psi, sheath gas temperature 350°C, sheath gas flow 11 liters/min, capillary (positive) 3500 V, capillary (negative) 3500 V, nozzle voltage (positive) 1000 V, nozzle voltage (negative) 100 V. A pooled QC sample, a dilution series of an analytical standard, and blank samples were injected at regular intervals throughout the run. Each sample was injected three times to acquire technical replicate data. Samples were injected in randomized order. External calibration based on standard curves was used to convert integrated peak areas to concentrations. Probabilistic quotient normalization (PQN) was used to normalize the concentration of samples (80). For each biological replicate, technical replicates were averaged by computing their mean value.

### Immunoblot

Killifish brains and cells treated for 24 hours with anisomycin (Cell Signaling Technology, 2222) were lysed as described in “Sample preparation for total proteome and analysis of posttranslational modifications.” For brain samples, male and female fish with a sex ratio 1:1 were used. Protein concentration was estimated by Qubit assay (Invitrogen, Q33211), and 30 μg of proteins were used. 4× loading buffer [1.5 M Tris pH 6.8, 20% (w/v) SDS, 85% (v/v) glycerin, 5% (v/v) β-mercaptoethanol] was added to each sample and then incubated at 95°C for 5 min. Proteins were separated on 4–20% Mini-Protean TGX Gels (BioRad, 4561096) by sodium dodecyl sulfate–polyacrylamide gel electrophoresis (SDS-PAGE) using a Mini-Protean Tetra Cell system (BioRad, Neuberg, Germany, 1658005EDU). Proteins were transferred to a nitrocellulose membrane (Carl Roth, 200H.1) using a Trans-Blot Turbo Transfer Starter System (BioRad, 1704150). Membranes were stained with Ponceau S (Sigma, P7170-1L) for 5 min on a shaker (Heidolph Duomax 1030), washed with Milli-Q water, imaged on a Molecular Imager ChemiDocTM XRS + Imaging system (BioRad) and destained by two washes with PBS and two washes in TBST (Tris-buffered saline (TBS, 25 mM Tris, 75 mM NaCl), with 0.5% (v/v) Tween-20) for 5 min. After incubation for 5 min in EveryBlot blocking buffer (Biorad, 12010020), membranes were incubated overnight with primary antibodies against RPS3 (Bethyl Laboratories, A303-840A-T), RPS10 (Abcam, ab151550) or α-tubulin (Sigma, T9026) diluted (1:1000) in enzyme dilution buffer [0.2% (w/v) bovine serum albumin (BSA), 0.1% (v/v) Tween20 in PBS] at 4°C on a tube roller (BioCote Stuart SRT6). Membranes were washed three times with TBST for 10 min at room temperature and incubated with horseradish peroxidase coupled secondary antibodies (Dako, P0448/P0447) at room temperature for 1 hour [1:2000 in 0.3% (w/v) BSA in TBST]. After three more washes for 10 min in TBST, chemiluminescent signals were detected using ECL (enhanced chemiluminescence) Pierce detection kit (Thermo Fisher Scientific, Waltham, MA, USA, #32109). Signals were acquired on the Molecular Imager ChemiDocTM XRS + Imaging system and analyzed using the Image Lab 6.1 software (Biorad). Membranes were stripped using a stripping buffer [1% (w/v) SDS, 0.2 M glycine, pH 2.5], washed three times with TBST, blocked, and incubated with the second primary antibody, if necessary.

### RNA isolation for RNA-seq analysis

Individual tissues were collected and snap-frozen in liquid nitrogen. For brain, fin, and heart, only male fish were used. For muscle and optic tectum, male and female fish with a sex ratio 1:1 were used. The protein amount was estimated on the basis of fresh tissue weight (assuming 5% of protein w/w), and ice-cold 1x PBS with protease/phosphatase inhibitors (Roche,11836170001, 4906837001) was added accordingly to a final concentration of 2 μg/μl. Samples were then vortexed (five times) before sonication (Bioruptor Plus) for 10 cycles (60 s ON/30 s OFF) at the high setting, at 4°C. The samples were then centrifuged at 3000*g* for 5 min at 4°C, and the supernatant was transferred to 2 ml Eppendorf tubes. One and a half milliliters of ice-cold Qiazol (Qiagen, 79306) reagent was added to 150 μl of homogenate, vortexed five times, and snap-frozen in liquid nitrogen. On the day of the experiment, samples were thawed on ice, vortexed five times, and incubated at room temperature for 5 min before adding 300 μl of chloroform. Samples were mixed vigorously, incubated for 3 min at room temperature, and centrifuged at 12,000*g* for 20 min at 4°C. The upper aqueous phase (600 μl) was carefully transferred into a fresh tube, and the remaining volume (phenol/chloroform phase) was kept on ice for DNA isolation. The aqueous phase was mixed with 1.1 volume of isopropyl alcohol, 0.16 volumes of sodium acetate (2 M; pH 4.0), and 1 μl of GlycoBlue (Invitrogen, AM9515) to precipitate RNA. After 10 min incubation at room temperature, samples were centrifuged at 12,000*g* for 30 min at 4°C. The supernatant was completely removed, and RNA pellets were washed by adding 80% (v/v) ethanol and centrifuging at 7500*g* for 5 min at 4°C. The washing steps were performed twice. The resulting pellets were air-dried for no more than 5 min and dissolved in 10 μl nuclease-free water. To ensure full dissolution of RNA in water, samples were then incubated at 65°C for 5 min, before storage at −80°C.

### RNA-seq library preparation

Sequencing of RNA samples was done using Illumina’s next-generation sequencing methodology (81). In detail, quality check and quantification of total RNA was done using the Agilent Bioanalyzer 2100 in combination with the RNA 6000 pico kit (Agilent Technologies, 5067–1513). Total RNA library preparation was done by introducing 500 ng total RNA into Illumina’s NEBNext Ultra II directional mRNA (UMI) kit (NEB, E7760S), following the manufacturer’s instructions. The quality and quantity of all libraries were checked using Agilent’s Bioanalyzer 2100 and DNA 7500 kit (Agilent Technologies, 5067–1506).

### RNA-seq sequencing

All libraries were sequenced on a NovaSeq6000 SP 300 cycles v1.5; paired-end 151 base pairs (one pair for each of the projects). Total RNA libraries were pooled and sequenced in three lanes. Small RNA libraries were pooled and sequenced in one lane. Sequence information was extracted in FastQ format using Illumina’s bcl2FastQ v2.20.0.422, against the *N. furzeri* reference genome (Nfu_20150522, annotation nfurzeri_genebuild_v1.150922). Alignment to the reference genome was performed using STAR (82) with the following parameters: --outSAMmultNmax 1 --outFilterMultimapNmax 1 --outFilterMismatchNoverLmax 0.04 --sjdbOverhang 99 --alignIntronMax 1000000 --outSJfilterReads Unique. The deduplication step was performed using the umi_tool v1.1.1 (83), using the following parameters: extract --bcpattern= NNNNNNNNNNN`, `dedup --chimeric-pairs discard --unpaired-reads discard --paired.

### RNA-seq quantification and differential expression

RNA-seq data were then processed as follows: quantification was performed using featurecounts v2.0.3 (84) with the following parameters -s 2 -p -B --countReadPairs. Differential expression analysis was performed using the DESeq2 package, (v1.34.0) Wald Test (85). *P* values were adjusted using the Benjamini-Hochberg method. Raw count data were normalized using the transcript per million strategy.

### Ribo-seq library preparation

Ribosome profiling libraries were prepared following previously published protocol with modifications (32). Ten to fifteen brain samples (primarily male) or 2 to 10 liver samples (equal ratio of male and female) from fish were combined for each biological replicate and lysed frozen using Cryo-Mill (Retsch, MM301) in the presence of 1 ml of lysis buffer (20 mM Tris-HCl pH 7.5, 140 mM KCl, 5 mM MgCl_2_, 1 mM DTT, 100 μg/ml cycloheximide, 1% Triton X-100, and 1 X protease inhibitor). Male and female fish with a sex ratio 1:1 were used. Lysed powder was quickly thawed in a water bath at room temperature and spun at 21,000*g* for 15 min at 4°C to clear lysate. RNAse I (Invitrogen, AM2294) was added to 0.4 U/μg of RNA and incubated at 25°C for 45 min. Digestion was stopped by adding 0.4 U/μg of SUPERaseIn RNAse Inhibitor (Invitrogen, AM2696). RNAse-treated lysate was layered on 900 μl sucrose cushion buffer (20 mM Tris-HCl pH 7.5, 140 mM KCl, 5 mM MgCl_2_, 1 mM DTT, 100 μg/ml cycloheximide, 0.02 U/μl SuperaseIn, 1M Sucrose), and spun at 100,000 rpm for 1 hour at 4°C in TLA100.3 rotor. The resulting ribosome pellet was resuspended in 250 μl of lysis buffer with SuperaseIn, and RNA was extracted using TRIzol reagent (Invitrogen, 15596026) following the manufacturer’s protocol. Twenty-seven to thirty-four nucleotide (nt) fragments were isolated from denaturing gel, ligated to adapter (NEB, S1315S), and ribosomal RNA was removed using RiboCop (Lexogen, 144.24) mixed with custom depletion DNA oligos (Table 3). The remaining fragments were reverse transcribed, circularized, and PCR-amplified following the steps described previously (9). Barcoded samples were pooled and sequenced using Hiseq 4000 (Illumina).

### Polysome profiling

Pooled brains from respective ages were lysed frozen using Cryo-Mill (Retsch, MM301) in the presence of 1 ml of lysis buffer (20 mM Tris-HCl pH 7.5, 140 mM KCl, 5 mM MgCl_2_, 1 mM DTT, 100 μg/ml cycloheximide, 1% Triton X-100, and 1 X protease inhibitor). Male and female fish with a sex ratio 1:1 were used. Lysed powder was quickly thawed in a water bath at room temperature and spun at 21,000*g* for 15 min at 4°C to clear lysate. The resulting supernatant was layered onto 12 ml 10%–50% sucrose gradient, spun at 39,000 rpm for 2.5 hours at 4°C in a SW41 rotor, and fractionated using a density gradient fractionator (ISCO). A260 of resulting fractions were measured on nanodrop, and respective species were pooled for quantitative proteomics by DIA LC-MS/MS.

### RNA isolation for tRNA-seq analysis

The RNA isolation for tRNA-seq analysis was carried out as previously described (86). In brief, individual brains from fish were collected and snap-frozen in liquid nitrogen. Male and female fish with a sex ratio 1:1 were used. One milliliter of ice-cold Qiazol (Qiagen, 79306) reagent was added to the tubes and the samples were homogenized using metallic beads using a QIAGEN tissue Lyser with 20 s ON two times, frequency = 30, and then immediately placed back on ice. Samples were incubated for 5 min at room temperature to allow nucleoprotein complexes to dissociate. After incubation, 0.2 ml of chloroform for every 1 ml of Qiazol reagent used during lysis was added. Samples were briefly vortexed and allowed to sit for 2 min at room temperature. The tubes were centrifuged at 12,000*g* for 15 min at 4°C in a prechilled centrifuge. Tubes were kept on ice, and the aqueous phase containing the RNA was carefully transferred to a new 1.5 ml microfuge tube. Again, 0.5 ml of chloroform was added for every 1 ml of Qiazol reagent, and after a brief vortex, the samples were centrifuged for 5 min at 12,000*g* at 4°C. The tubes were kept on ice, and the aqueous phase containing the RNA was transferred to a fresh 2 ml microfuge tube. RNA was precipitated by adding 25 μg of glycogen, 100 μl of 3 M sodium acetate (pH = 4.5), and 1.25 ml of ice-cold 100% ethanol for every milliliter of Qiazol reagent used. Finally, the samples were vortexed thoroughly and incubated at −20°C for at least 30 min before precipitation. Pellets were washed once in 80% ice-cold ethanol supplemented with 50 mM sodium acetate (pH = 4.5), briefly air-dried, resuspended in 50 mM sodium acetate (pH = 4.5), 1 mM EDTA and stored at −80°C.

### tRNA-seq library construction and data analysis

Total RNA purified under mildly acidic conditions was subjected to oxidation and beta-elimination as previously described (86). Ten nanograms synthetic *E. coli* tRNA-Lys-UUU-CCA and *E. coli* tRNA-Lys-UUU-CC mixed in a 3:1 ratio were added as a spike-in, followed by RNA 3′ end dephosphorylation with T4 PNK (NEB, M0201S). After ethanol precipitation, RNA of 60 to 100 nt was purified by size selection on a 10% polyacrylamide/7M urea/1×TBE gel. Following elution from gel slices, size-selected RNA was ligated to one of eight barcoded 3′-adapters (86) for 3 hours at 25°C in 1×T4 RNA ligase buffer, 25% PEG-8000, 20 U Superase In (Thermo Fisher Scientific, AM2696) and 1 μl T4 RNA Ligase 2, truncated KQ (NEB, M0373S). Ligation products were pooled and separated from unligated 3′-adapters by size selection on a 10% polyacrylamide/7M urea/1×TBE gel. One hundred nanograms of pooled adapter-ligated RNA was annealed with 1 μl of 1.25 μM RT primer (86) at 82°C for 2 min and 25°C for 5 min. Reverse transcription was carried out with 500 nM TGIRT (InGex, TGIRT50) at 42°C for 16 hours in 50 mM Tris-HCl pH = 8.3, 75 mM KCl, 3 mM MgCl_2_, 5 mM DTT, 1.25 mM dNTPs and 20 U Superase In (Thermo Fisher Scientific, AM2696). RNA templates were hydrolyzed by the addition of NaOH to 0.1 M and incubated for 5 min at 90°C. cDNAs > 10 nt longer than RT primer were excised from 10% polyacrylamide/7M urea/1×TBE gels after SYBR Gold staining. Following elution from gel slices, cDNA was circularized with TS2126 Rnl1 (commercially sold as CircLigase) for 3 hours at 60°C in 50 mM MOPS pH 7.5, 10 mM KCl, 5 mM MgCl_2_, 10 mM DTT, 0.05 mM ATP, 2.5 mM MnCl_2_, and 1 M betaine. The enzyme was inactivated at 80°C for 10 min. One-quarter of the circularized cDNA was used for library construction with KAPA HiFi DNA Polymerase (Roche) as previously described (86). The resulting libraries were quantified with the Qubit dsDNA HS kit (Thermo Fisher Scientific, #Q32851) and sequenced for 120 cycles on an Illumina NovaSeq 6000 platform. Sequencing reads were adapter-trimmed and demultiplexed with cutadapt v3.5 (87). The high-confidence set of predicted tRNA genes in the nuclear *N. furzeri* genome were obtained from the Genomic tRNA database [GtRNAdb (88)]. Mitochondrial tRNA annotations were obtained from GenBank accession NC_011814.1. tRNA expression and charging were analyzed with v1.3.8 of the modification-induced misincorporation tRNA sequencing (mim-tRNAseq) computational package (89) using the following command: -t notFur1_tRNAs.fa -m notFur1-mitotRNAs.fa -o notFur1-tRNAs-confidence-set.out --cluster-id 0.95 --threads 40 --control-condition young --max-mismatches 0.1 –remap --remap-mismatches 0.075 --max-multi 8.

### Imaging

#### Cryosections preparation and free-floating immunofluorescence:

To prepare brain cryosections for free-floating immunofluorescence from 5 wph and 39 wph killifish, brains were dissected and fixed ON in a solution of 4% paraformaldehyde PFA in PBS at 4°C. The samples were then equilibrated in a 30% sucrose solution ON at 4° and subsequently embedded in cryoprotectant (Tissue -Tek O.C.T. Compound; Sakura Finetek, USA). Tissue slices of 50 mm thickness were cut at a cryostat (Leica) and stored on glass slides (Thermo Fisher Scientific, USA).

Free-floating immunofluorescence experiments were performed by adapting previous protocols for classical on-slide immunofluorescence (90). Briefly, the sections were washed in PBS to remove the cryoembedding medium and detached from the glass slide. The sections were then placed in 24 wells and underwent two additional washes in PBS for 5 min each. Afterward, an acid antigen retrieval step (10 mM Tri-sodium citrate dihydrate, 0.05% tween, at pH 6) was performed by bringing the solution to boiling point in a microwave and adding 50 ml of it in each well, leaving the solution for 5 min. This step was repeated two times. 500 ml of blocking solution (5% BSA, 0.3% Triton-X in PBS) was then applied for 2 hours. Primary antibodies (Phospho-Tau AT100, NeuN or Lamp1 Table 4) at the proper dilution were added in a solution of 1% BSA, 0.1% triton in PBS, and left overnight at 4°C in slow agitation on a rocker. The next day, the proper secondary antibodies (Table 4) at a 1:500 dilution were used in the same solution. After 2 hours of incubation, slices were washed three times with PBS, counterstained with a solution 1:10,000 of Hoechst 33342 (Invitrogen, USA) for 2 min, and manually mounted under a stereomicroscope on Superfrost Plus glass slides (Thermo Fisher Scientific, USA). Finally, Fluoroshield mounting medium (Sigma, USA) was used, and slices were covered with a coverglass (Thermo Fisher Scientific, USA).

#### Image acquisition:

Imaging of lysosomal staining was performed with a Zeiss scanning confocal microscope (LSM900, Zeiss, Germany) equipped with an Airyscan module. Nine consecutive z planes with a step of 300 nm were acquired with a 63x oil immersion objective (Plan-Apochromat 63x/1.4 Oil DIC M27, Zeiss, Germany) at a resolution of 2186 by 2186 pixels with the use of Airyscan. Images were then deconvoluted in the Zeiss Zen blue 3.7 suite using the Fast Iterative algorithm and exported as tiff for further analysis in Imaris (Bitplane, UK).

Samples processed for Tau stainings were imaged with an Axio Imager Z.2 (Zeiss, Germany) equipped with an Apotome slide using a 63x oil immersion objective (Plan-Apochromat 63x/1.4 Oil DIC M27, Zeiss, Germany). Z-stacks were realized by acquiring five consecutive z-planes at an interval of 1 μm. Images were then processed in ImageJ (Fiji).

### Lysosomes morphological analysis

Analysis of the sections was performed blindly. To analyze the change in morphology of lysosomes in aging, we analyzed nine 5 wph (six females, three males) samples and twelve 39 wph samples (six males, six females). To study morphological changes in case of proteostasis alteration, samples from six bortezomib-treated animals and six controls (DMSO-treated) were analyzed. Male and female fish with a sex ratio 1:1 were used. Tiff images were loaded in Imaris (Bitplane, UK) to recreate a 3D rendering of the samples. A version of the “Surfaces” algorithm was created, optimizing the settings to realize an optimal mask of single lysosomes. Statistics obtained (area, volume, mean intensity, and sphericity) were extracted, and mean values for each animal were calculated. Data significance was tested using a two-tailed *t* test.

### Mean fluorescence intensity analysis

To analyze differences in the amount of tau phosphorylation between young (5 wph) and old (39 wph) killifish brain samples, we performed mean fluorescence intensity (MFI) analysis in the free license software ImageJ (Fiji). Since Tau is a neuronal protein, and the number of neurons between young and old animals varies, we normalized the MFI of Tau staining over the MFI of NeuN, a neuronal-specific marker, to render the Tau MFI proportional to the number of neurons. Images were opened in ImageJ (Fiji), and median filtering (1px radius) was applied. The average intensity projection was realized, and MFI for the green channel (tau) and red channel (NeuN) was measured and reported in an Excel table. Tau MFI for each animal was divided by the corresponding NeuN MFI, and the significance of the results was tested by a two-tailed *t* test.

### Data analysis

#### Protein subcellular localization by LOPIT-DC:

For each age group and replicate, protein distribution profiles were calculated by dividing the scaled protein quantity in each fraction by the total sum of protein quantity across all fractions. Protein markers for the different compartments were taken from the Bioconductor package pRoloc (91), by mapping *N. furzeri* entries onto *Homo sapiens* entries via orthologs mapping. To classify each of the proteins into a stable compartment, a support-vector-machine classifier with a radial kernel (92) was used. Hyper-parameters *C* and *gamma* were selected via a grid-search approach using a fivefold cross-validation iterated 100 times. The best *C* and *gamma* parameters were selected to classify the “unknown” proteome. Only classified proteins with an SVM score > 0.7 were considered stable classification. To detect age-related changes in subcellular fractionation, a two-step approach was implemented. For each normalized protein profile, a PCA was used to summarize the variance from the 10 fractions in each replicate and age group. After summarization, the first two principal component scores were used to perform a Hotelling T^2^ test to detect changes in the multivariate protein profile mean. To estimate effect sizes, the median Euclidean distance between age groups was calculated for each protein profile (fig. S4F).

#### Differential detergent extraction:

A batch correction was applied to remove the effects of different batches of LC-MS/MS analysis using the limma::removeBatchEffect function from the limma package (77). Then, for each protein group, a detergent insolubility profile was generated by dividing the protein quantities from fractions F1:F3 by the quantity in the soluble (S) fraction, and log_2_ transformed. To detect significant changes in detergent insolubility profiles between age groups, a multivariate analysis of variance (MANOVA) test was applied to the detergent insolubility profiles using the standard function in the R programming language, and *P* values were corrected for multiple testing using the FDR strategy. To estimate effect sizes, a detergent insolubility score (DIS) was calculated by summing the log_2_ transformed protein quantities in fractions F1:F3 relative to the S “soluble” fraction. For each age group and protein group, the median DIS between replicates was used to estimate the magnitude of changes in detergent insolubility: ΔDIS = DIS_39wph_ − DIS_12wph_. High values of ΔDIS indicate proteins that become more detergent resistant in the old (39 wph) samples (fig. S3F).

#### Modified peptide abundance correction:

For each enrichment, PTMs report tables were exported from Spectronaut. To correct the quantities of modified peptides for underlying changes in protein abundance across the age groups compared, correction factors were calculated using the aging proteome data. For each condition and protein group, the median protein quantity was calculated and then divided by the median protein quantity in the young (5 wph) age group. Each modified peptide was matched by protein identifier to the correction factor table. If a modified peptide was mapped to two or more proteins, the correction factor was calculated using the sum of the quantity of these proteins. Further, the correction was carried out by dividing peptide quantities by the mapped correction factors, and log_2_ transformed (fig. S5B). Differences in peptide quantities were statistically determined using the *t* test moderated by the empirical Bayes method as implemented in the R package limma (77).

#### Kinase activity prediction from phosphoproteome data:

Kinase activity prediction was calculated using the Kinase library (https://kinase-library.phosphosite.org) (93) using the differential expression-based analysis and default parameter.

#### GO enrichment analysis:

GSEA was performed using the R package clusterProfiler (94), using the function gseGO. Briefly, *N. furzeri* protein entries were mapped to the human gene name orthologs and given in input to the function to perform the enrichment. For GO term overrepresentation analysis (ORA), the topGO R package was used.

#### Identification of conserved PTM sites:

For the *N. furzeri* proteins involved in neurodegenerative diseases (fig. S5I), a local alignment was performed with protein BLAST(v2.12.0+) (95) with default parameters against the RefSeq human proteome (Taxon ID:9606). The top 10 hits from the BLAST search were retrieved, and each modified residue was mapped into the local alignment to identify the corresponding position in the human proteins. Each modified peptide was then considered conserved if at least one of the top 10 hits from the BLAST alignment had a corresponding residue in the modified amino acid position.

#### Calculation of protein-transcript decoupling and multiple linear regression:

For aging brain proteome data and proteasome inhibition samples, protein-transcript decoupling values were calculated as the difference in log_2_ fold changes between proteome and transcriptome. A null distribution was fitted on the decoupling values using the R package fdrtool (96). *Q* value < 0.1 was used as a threshold to reject the null hypothesis. The decoupling values from each protein-transcript pair were used as response variables in a multiple linear regression model. Predictors for the model were retrieved as follows: Protein quantities were calculated as the median log_2_ protein quantity across all replicates from the proteomics DIA data. Protein quantities are estimated using the median peptide abundance as calculated by the Spectronaut software. mRNA abundance values were defined as the median log2_2_(TPM) across all samples from the RNA-seq aging dataset. Biophysical parameters were calculated for each protein with the R package Peptides. Protein half-life values were taken from mouse cortex data from (18). The percentage of gene GC content was obtained from ENSEMBL Biomart (v108) (97), mapping ENSEMBL annotation against the *N. furzeri* reference genome (Nfu_20150522, annotation nfurzeri_genebuild_v1.150922) using bedtools (98). Multiple linear regression models were then performed using the “lm” base R function by keeping only complete and unique observations from the matrix generated. Features were scaled for each dataset, and a multiple linear regression model without intercept was fitted to the data.

### Data integration

Log_2_ fold changes (for PTMs), ΔDIS (for detergent insolubility), or protein-transcript decoupling score values were used as input for a GSEA analysis based on GO cellular component terms using the gseGO function from the clusterProfile (94) R package with the following parameters: minSize = 5 and maxSize = 400. For each GSEA, the NES were taken and arranged in a matrix with different GO terms as rows and different datasets as columns. A PCA was performed on the matrix to visualize the relationship between the datasets. Missing GO terms in a given dataset were imputed as 0 values. The sum of the scores on the first two principal components was used to extract the most strongly affected GO terms from the combined integration of all the datasets.

### Mitochondrial proteome composition

To calculate age-related changes in mitochondrial proteome composition (Fig. 2H), raw DIA files coming from fraction 02 of the LOPIT-DC experiment were reanalyzed in Spectronaut (v16.2), using the same parameters as the other LOPIT-DC experiment. Fraction 02 represents the fraction where mitochondrial proteins are sedimenting in the LOPIT-DC experiment and, therefore, strongly enriched for mitochondrial proteins (fig. S4, C and D). From the protein quantity matrix, mitochondrial proteins [according to Mitocarta3.0 annotation (99)] were extracted, and their quantities log_2_ transformed and normalized by median centering. To detect changes in composition, a linear model on the log_2_ mitochondrial-centered values was implemented between the two age groups with the R package limma (77).

### Ribo-seq data processing and analysis

Data processing and analysis was based on previously published protocol (32). Adapter sequences were removed from demultiplexed sequencing reads using Cutadapt v.1.4.2 (87), followed by the removal of the 5′ nucleotide using FASTX-Trimmer. Reads mapping to ribosomal RNAs were removed using Bowtie v.1.3.1 (100). The remaining reads were aligned to reference libraries that consisted of coding sequences containing 21 nucleotides flanking upstream of the start codon and downstream of the stop codon. To maximize unique mapping, a reference library was constructed using the longest transcripts for every 22,757 genes. Bowtie alignment was performed using the following parameters: -y -a -m 1 -v 2 -norc -best -strata. A-site offset was estimated using riboWaltz (101), and fragment lengths that do not exhibit 3-nt periodicity were removed. Pause scores at each position were calculated by dividing the number of reads at each position by the average number of reads within the internal part of the transcript, excluding the first and last 20 codons. Positions with increased pausing during aging were identified following the previously published method (32). Briefly, for 6749 transcripts with sufficient coverage (>0.5 reads/codon and >64 reads/transcript) in all age groups, we used a two-tailed Fisher’s exact test to compare each position (codon) between age groups to identify positions with statistically significant changes (Benjamini-Hochberg adjusted *P* < 0.05). These positions were further filtered to include positions with odds ratio greater than 1, pause score of the older sample greater than the pause score of the younger sample, reads in the oldest sample greater than the average number of reads across the transcript, and a position in the internal part of the transcript to only select sites with high-confidence age-dependent changes in pausing. To visualize amino acids enriched in age-dependent pausing sites, we used the weighted Kullback-Leibler method (102) using the frequency of each amino acid in coding sequences as background. For metagene analysis around age-dependent pausing sites, reads were first aligned to these sites and normalized by dividing reads at each codon by the average reads per codon within the analysis window to control for differences in expression and coverage. Mean and bootstrapped 95% confidence intervals of these normalized values were plotted. Only positions with sufficient coverage (reads/codon > 0.5) in the analysis window were included. To identify sites with disome formation, we first identified sites with strong pausing in the old sample (pause score > 6). Then, we calculated the average ribosome density of two regions for young and old samples: region 1, analysis window (40 codons up/downstream from strong pause site); and region 2, between 8 and 12 codons upstream from strong pause site (approximate position of trailing ribosome). Sites with higher ribosome density in region 2 were identified as disome sites, and disomes sites unique to old samples were plotted. For comparisons to proteomics datasets, we included all sites with statistically significant changes (Benjamini-Hochberg adjusted *P* < 0.05) and used log_2_ of pause score ratio (old/young).

For translation efficiency analysis, RNA-seq data was realigned to the same reference library used for Ribo-seq to compare transcript abundance. Changes in translation efficiency were calculated using DESeq2 (85), using the following design ~assay + condition + assay:condition, where assay indicates the different counts from RNA-seq and Ribo-seq, respectively, and condition indicated the different age groups.

### Estimates of mRNA half-life variations

mRNA half-life variations were estimated according to (41) under the assumption of steady state for premature and mature RNA. Specifically, variations in mRNA half-life for every gene are estimated using the ratio of the number of exonic reads and intronic reads. The first mostly reflects the total abundance of transcript, precursor + mature, which is determined by the balance between mRNA synthesis and degradation, to the number of intronic reads, reflecting the abundance of the precursor RNA determined by mRNA synthesis. An increase of exons/ intron ratio in steady state conditions therefore indicates an increase in mRNA stability. To compute these values, exonic coordinates of protein-coding genes were extracted from the annotation nfurzeri_genebuild_v1.150922. Exonic and intronic read counts were obtained following the procedure suggested by (41). To this end, exonic coordinates were flanked on both sides by 10 nt and were grouped by gene. Intronic coordinates were obtained by subtracting the exonic coordinates from the gene-wise coordinates. For each gene, exonic and intronic read counts were obtained using the htseq-count function from HTSeq v2.0.2 (103) with the parameter -m set to intersection-strict to consider only reads that strictly fall within an exon or an intron. Additionally, in each sample, genes with <10 reads on both exons and introns were ignored (read counts set as missing values) in order to be robust against noisy estimates based on low read counts. Lastly, the log-transformed exonic-to-intronic read count ratio *r* was computed for each gene and sample as

$$r={\text{log}}_{2}\left(\text{exonic}\;\text{counts}+1\right)-{\text{log}}_{2}\left(\text{intronic}\;\text{counts}+1\right)$$

Gene-specific biases such as exonic and intronic lengths and GC content can affect exonic and intronic read counts. These biases cancel out when ratios between samples are considered, as they are typically multiplicative (41). The ratio between mRNA half-life in sample s_1_ and sample s_2_ is then estimated as

$${\text{log}}_{2}\left({\text{mRNA}\;\text{half-life}}_{{\text{S}}_{1}}/{\text{mRNA}\;\text{half-life}}_{{\text{S}}_{2}}\right)=r_{1}/r_{2}$$

### Estimates of protein synthesis rate

To estimate *k*_*i*_, 5′UTR sequences were retrieved from the *N. furzeri* reference genome (Nfu_20150522, annotation nfurzeri_genebuild_v1.150922). The masked FASTA genome sequences were parsed using bedtools (98). The translation starting codon “ATG” was identified from the “CDS” features from the GFF file. The region around the starting codon was extracted with +6 nucleotide upstream and +4 nucleotide downstream to match the pattern “NNNNNNATGNN.” Only valid sequences (without ambiguous nucleotides) with an ATG starting codon in the correct position were retained. Ninety-one percent of the transcript annotated in the GFF file had a valid translation initiation region as described above. The *k*_*i*_ was then estimated using the dinucleotide position weight matrix from (44). In case a single transcript had multiple starting sites, the *k*_*i*_ values were summarized by taking the median value. This led to the estimate of *k*_*i*_ for 59,129 transcripts. Estimated protein synthesis rates were calculated as in (42, 43). More in detail, the authors described the estimated synthesis rate as

$$Q=mRk_{i}\left[1-\left(L/\left(\left(k_{e}/\left(k_{i}R\right)\right)+\left(L-1\right)\right)\right)\right]$$

Where *Q* refers to the estimated synthesis rate, *m* refers to individual mRNA expression level obtained from the median across sample log_2_(TPM) from RNA-seq data and normalized between 0 and 1, *R* represents the total amount of available ribosomes, *k*_*i*_ indicates an mRNA-specific translation initiation rate as computed above and normalized between 0 and 1, *L* is the number of codons occupied by one ribosome, set to 10 (based on the average length of a ribosome footprint), and *k*_*e*_ is the termination rates arbitrarily set to 1. Estimated synthesis rates were then computed for different values of *R* ranging from 1.3 to 0.

### Figure generation

Some of the icons used in the figures were created with BioRender.com.

### Data and code availability

See Table 5 for dataset availability. A shiny app for data exploration and visualization is accessible at https://genome.leibniz-fli.de/shiny/orilab/notho-brain-atlas/. Custom R codes used for data analysis and visualization of Ribo-seq data are available in GitHub (https://github.com/leejh11/riboseq_analysis) and Zenodo (104). The code and R package for reproducing analysis and figures of this manuscript is available in GitLab (https://gitlab.leibniz-fli.de/ddifraia/multiomics-r-package) and Zenodo (105).

## Supplementary Material

Supplementary Material

science.org/doi/10.1126/science.adk3079

Supplementary Text; Figs. S1 to S13; References (119–130); MDAR Reproducibility Checklist

## Figures and Tables

**Table 1.** Ultracentrifugation settings for LOPIT-DC (localization of organelle proteins by isotope tagging after differential ultracentrifugation) protocol.

*g*	Time (min)	Fraction	Temperature
3,000	10	02	4°C
5,000	10	03	4°C
9,000	15	04	4°C
12,000	15	05	4°C
15,000	15	06	4°C
30,000	20	07	4°C
79,000	43	08	4°C
120,000	45	09	4°C
—	—	10 (final supernatant, cytosol enriched)	—

**Table 2.** Settings used for MS data analysis on Spectronaut software.

Dataset	Software version	Test	Data filtering	Imputation	Normalization
Brain, optic tectum, liver, and muscle proteome	15.3.2/18.6.2	Unpaired *t* test	*Q* value	Global imputing	True, automatic
Fin	14.9.2	Paired *t* test	*Q* value	None	Local, automatic
LOPIT-DC	14.9.2	NA	*Q* value percentile 0.2	Run wise imputing	True, global
Polysome profiling	18.7.2	NA	*Q* value	None	Local, automatic
Detergent insolubility	15.4.2	NA	*Q* value percentile 0.2	Run wise imputing	False
Proteasome inhibition	14.9.2	Unpaired *t* test	*Q* value	Global imputing	True, automatic
Proteasome inhibition, old animals	18.7.2	Unpaired *t* test	*Q* value	Global imputing	True, automatic
PTMs–ubiquitylation	15.4.2	–	*Q* value percentile 0.2	Global imputing	True, automatic
PTMs–phosphorylation	15.4.2	–	*Q* value percentile 0.2	Global imputing	True, automatic
PTMs–acetylation	15.4.2	–	*Q* value percentile 0.2	Global imputing	True, automatic

NA, not applicable.

**Table 3.** List of DNA oligonucleotides used for ribosomal RNA depletion.

Number	Sequence
Oligo #1	GGCCGTTACCGGCCTCACACCGTCCATGGGATGAGC/3BioTEG/
Oligo #2	CGGGCGAGACGGGCCGGTGGTGCGCCCGGGAAC/3BioTEG/
Oligo #3	CGCCTCCCCGCCTCACCGGGTAAGTGAAAAAACGATAAGAG/3BioTEG/
Oligo #4	GCACGCGCCGGGCGCTTGACACCAGAACCGAGAGC/3BioTEG/

**Table 4.** List of antibodies used in this work.

Antibody	Producer	Catalog number	RRID	Type	Working dilution
*Primary antibodies*					
Lamp1	Abcam	Ab24170	AB_775978	Polyclonal rabbit	1:500
NeuN	Abcam	Ab177487	AB_2532109	Monoclonal rabbit	1:500
Phospho-Tau AT100	Thermo Fisher Scientific	MN1060	AB_223652	Monoclonal mouse	1:400
*Secondary antibodies*					
AlexaFluor 488 anti-Rabbit	Invitrogen	A11001	AB_2534069	Goat IgG	1:500
AlexaFluor 568 anti-Rabbit	Invitrogen	A11011	AB_143157	Goat IgG	1:500
AlexaFluor 488 anti-Mouse	Invitrogen	A11004	AB_2534072	Goat IgG	1:500

RRID, research resource identifier; IgG, immunoglobulin G.

**Table 5.** Dataset availability.

Dataset	Repository	Accession ID
Brain aging and proteasome inhibition in adult killifish (proteome)	https://massive.ucsd.edu	MSV000091926 (106)
Proteasome inhibition in old killifish (proteome)	https://massive.ucsd.edu	MSV000095078 (107)
Optic tectum aging (proteome)	https://massive.ucsd.edu	MSV000095063 (108)
Liver aging (proteome)	https://massive.ucsd.edu	MSV000095062 (109)
Muscle aging (proteome)	https://massive.ucsd.edu	MSV000095074 (110)
Heart aging (proteome)	https://massive.ucsd.edu	MSV000095850 (111)
Posttranslational modifications	https://massive.ucsd.edu	MSV000091915 (112)
LOPIT-DC (subcellular fractionation)	https://massive.ucsd.edu	MSV000091916 (113)
Detergent insolubility (proteome)	https://massive.ucsd.edu	MSV000091933 (114)
Polysome fractions proteomic analysis	https://massive.ucsd.edu	MSV000095080 (115)
Parallel reaction monitoring data	https://massive.ucsd.edu	MSV000095079 (116)
Brain aging (RNA-seq) and ribosome profiling	https://www.ncbi.nlm.nih.gov/geo/	GSE232465
Proteasome inhibition in adult killifish (RNA-seq)	https://www.ncbi.nlm.nih.gov/geo/	GSE236685
Targeted metabolomics data	https://data.mendeley.com/	10.17632/4hw52rnjrj.1 (117)
Heart, muscle, fin, optic tectum aging, and proteasome inhibition of old killifish (RNA-seq)	https://www.ncbi.nlm.nih.gov/geo/	GSE277507
Liver aging (RNA-seq)	https://www.ncbi.nlm.nih.gov/geo/	GSE66712 (118)
Liver aging ribosome profiling	https://www.ncbi.nlm.nih.gov/geo/	GSE277506
Brain aging (tRNA-seq)	https://www.ncbi.nlm.nih.gov/geo/	GSE277509

## Data Availability

All data generated in the study have been deposited in public databases. Please refer to Table 5 for accession numbers for different datasets.
