# Supplementary material for: Altered translation elongation contributes to key hallmarks of aging in the killifish brain
Source: Science. Author manuscript; Available in PMC 2026 Jul 10. (PMC13353800; doi:10.1126/science.adk3079)
Supplement: Supplementary Material [file NIHMS2190114-supplement-Supplementary_Material.pdf]

Science
AAAS

Supplementary Materials for

Altered translation elongation contributes to key hallmarks of aging in the killifish brain

Domenico Di Fraia, Antonio Marino, Jae Ho Lee *et al.*

Corresponding authors: Judith Frydman, jfrydman@stanford.edu; Alessandro Cellerino, alessandro.cellerino@leibniz-fli.de; Alessandro Ori, alessandro.ori@leibniz-fli.de

Science **XXX**, XXXX (2025)
DOI: XXX

The PDF file includes:

Supplementary Text
Figs. S1 to S13
References (*119-130*)

Supplementary text:

Aging can influence different aspects of protein homeostasis. To obtain an unbiased characterization of the effect of aging on the brain proteome, we employed a multi-layered approach to interrogate major modes of protein regulation. We generated datasets describing changes in protein and mRNA abundance, protein subcellular localization, detergent insolubility, and post-translational modifications (PTMs) in the aging brain of killifish (Fig. 2A and S4A). First, we captured proteome-wide variation in subcellular localization using an approach based on differential centrifugation coupled with quantitative mass spectrometry (LOPIT-DC) (*23*). We analyzed pools of adult (12 weeks post-hatching = wph) and old (39wph) killifish brains (Fig. S4B and Table S4). We used a list of well-annotated organelle markers (*119*) to evaluate organelle separation by LOPIT-DC (Fig. S6A and S4 C through D) and to confirm the reproducibility of organelles sedimentation between adult and old brains (Fig. S4E). We then employed a tailored statistical approach (see methods, Fig. S4F) to identify age-dependent changes in protein sedimentation profiles (Fig. S6B and Table S4). The most prominent changes affected multiple mitochondrial and lysosomal proteins, among others, including the mitochondrial transporters SLC25A32 and SLC25A18 and the lysosomal and vesicle trafficking proteins RAB14 and CCZ1 (Fig. S6C). We interpret these alterations of sedimentation as an indication of partial reorganization of the mitochondrial and lysosomal proteome during aging that correlates with the well-described dysfunction of these organelles during aging and neurodegenerative diseases.

In parallel, we used the same pools of samples to assess age-dependent changes in protein solubility. We complemented our previous analysis of SDS insoluble aggregates in the killifish aging brain (*6*) with a more fine-grained analysis of protein solubility. Thus, we exposed brain homogenates to a series of detergent combinations of increasing strength, separated soluble and insoluble fractions by ultracentrifugation (as described in (*22*), Fig. S3A and Table S4), and quantified protein abundances across fractions using mass spectrometry. Principal component analysis showed reproducible detergent-based fractionation in adult and old brains (Fig. S3B), and GO enrichment analysis confirmed the expected partitioning of cellular components as a function of detergent strength (Fig. S3 C and D). In agreement with previous findings from other species (*15*, *120*) and the spontaneous age-related accumulation of protein aggregates in the killifish brain (*5–7*), we observed an overall increase of protein detergent-insolubility in old samples (Fig. S3E). By comparing detergent insolubility profiles between adult and old brains (Fig. S3 F through G), we identified 410 protein groups changing detergent insolubility with aging (Fig. S6D and Table S4). While many of these proteins exhibited increased insolubility to detergents in old brains, there were instances where aging was linked to decreased insolubility to detergents. This indicates that factors other than protein aggregation, such as protein interaction or localization alterations, could be responsible for the observed changes in detergent insolubility.

Next, we examined the effects of brain aging on multiple PTMs, using a sequential enrichment strategy followed by quantification of age-dependent changes in protein ubiquitylation, acetylation, and phosphorylation in the aging brain (Fig. S5A and Table S5). We quantified PTM-carrying peptides normalized for protein changes (see methods, Fig. S5) and identified age-related changes for 534 phosphorylated, 618 ubiquitylated, and 190 acetylated peptides ($P<0.05$, Fig. S6E). The general increase in the number of affected PTM peptides with aging emphasized its overall impact on the proteome beyond protein abundance (Fig. S6 E through F). Integration of phosphorylation data with experimentally derived kinase-substrate relationships (*93*) indicates a remodeling of kinase signaling in the aging brain. Besides an increased activity (i.e., increased phosphorylation of predicted targets) for kinases involved in the regulation of immune responses, we reported enhanced activity for kinases

of the protein kinase C family, e.g., PKN1, PKN2, PKCA, whose hyperactivation is linked to Alzheimer's disease (*121–123*). Our data also reveals the decreased activity of kinases responsible for the phosphorylation of splicing factors and other RNA processing proteins, e.g., CDC2-like kinases 2 and 4 (CLK2 and CLK4, Fig. S6 G through H). These data suggest a convergence between aging and neurodegeneration concerning altered signaling pathways in the brain and hints at dysfunctional RNA processing in the aging brain.

To more systematically investigate the convergence between brain aging and neurodegenerative diseases, we queried our datasets for killifish orthologs of proteins encoded by genes genetically linked to neurodegeneration in humans (Table S6). We found several of these proteins to be affected by aging in killifish in at least one of the proteomic datasets analyzed (Fig. S6I). These include changes in subcellular fractionation and detergent insolubility (Fig. S7 A and B) and 23 PTM sites conserved between killifish and humans (Fig. S7 C through E). The microtubule-associated protein Tau (MAPT) was notably affected by aging across multiple proteomic layers. MAPT showed a prominent increase in detergent insolubility in old brains (Fig. S6D), an alteration associated with human aging and neurodegenerative diseases (*124–126*). Additionally, we detected an age-dependent increase in phosphorylation and ubiquitylation of conserved residues in the microtubule-binding domain (MBD) of MAPT, a region sensitive to PTMs and associated with Tau pathological aggregation (Fig. S6J and S7D) (*126–128*). We validated the spontaneous increase of MAPT/Tau phosphorylation in old killifish brains using immunofluorescence staining for a conserved phosphorylated epitope of Tau (AT100) (Fig. S6K).

Together, our analyses comprehensively establish how aging affects the brain proteome along multiple axes beyond protein abundance. This thorough proteome characterization reveals several potential connections between aging, specific molecular events, and genetic factors associated with neurodegeneration, which are relevant to humans. To make this resource easily accessible to the scientific community, we have developed a web application at https://genome.leibniz-fli.de/shiny/orilab/notho-brain-atlas/

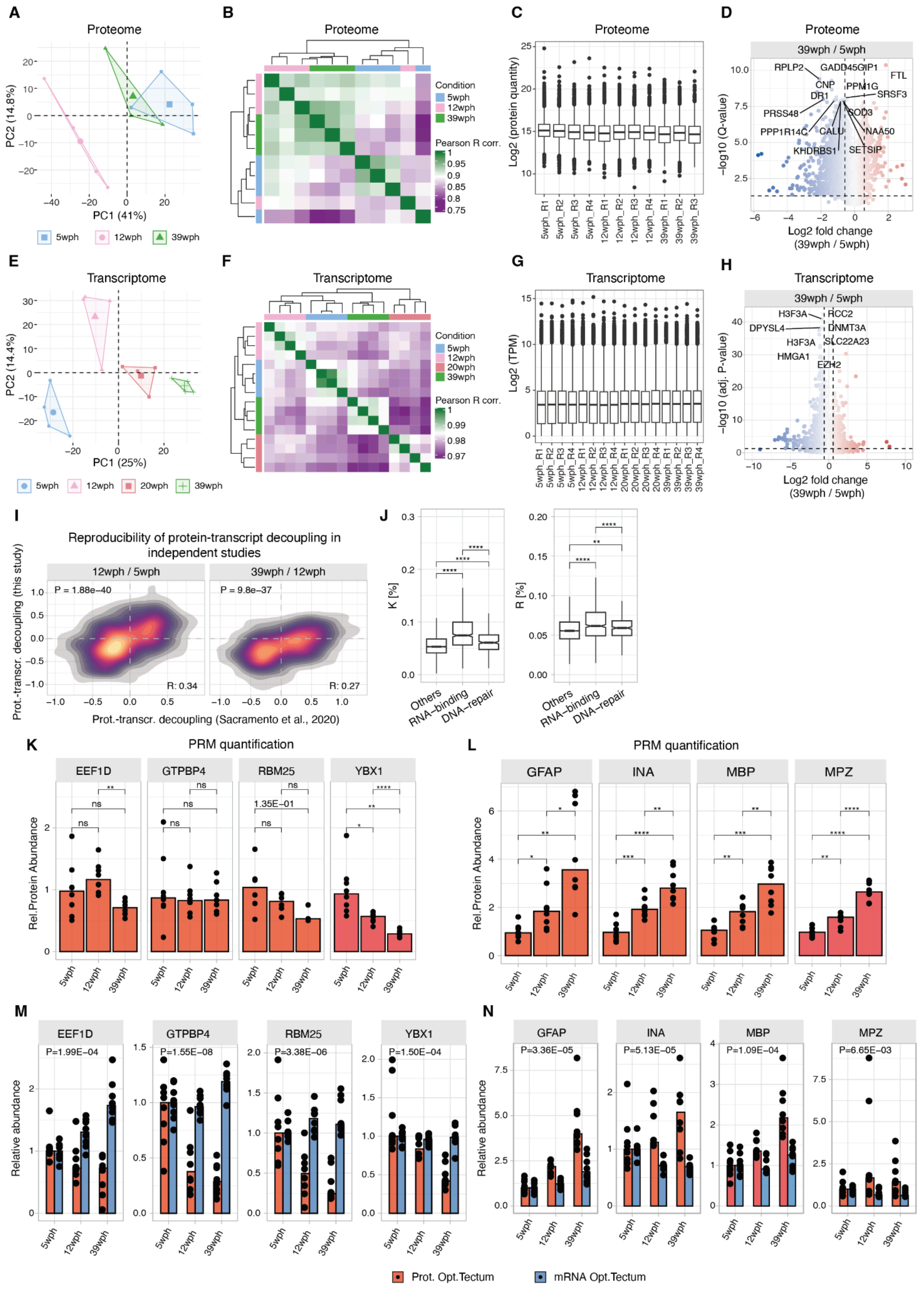

Figure S1: Proteome and transcriptome characterization of the killifish aging brain. A) Principal component analysis of proteomics data. B) Correlation heatmap between samples from the aging brain proteome data. Pairwise Pearson's R correlation coefficient was calculated using log2 transformed protein abundances. C) Boxplot displaying the distribution of

log2 transformed and normalized protein abundances. D) Volcano plot highlighting significant protein abundance changes in the aging brain (39wph vs. 5wph). Dashed lines indicate the threshold used to select differentially abundant proteins (absolute log2 fold change > 0.58 and Q < 0.05). E) Principal component analysis of transcriptomics data. F) Correlation heatmap between samples from the aging brain transcriptome data. Pairwise Pearson's R correlation coefficient was calculated using log2 transformed transcript per million reads (TPM). G) Boxplot displaying log2 transformed and normalized transcript counts (TPM) distribution. H) Volcano plot highlighting significant transcript abundance changes in the aging brain (39wph vs. 5wph). Dashed lines indicate the threshold for selecting differentially expressed genes (absolute log2 fold change > 0.58 and Adj. P < 0.05). For display purposes, the X-axis range was limited to a -10:10 range, leading to the exclusion of 1 gene. I) 2-D density plot showing the correlation between age-related protein-transcript decoupling based on the datasets generated in this study (y-axis) and a previous study (*6*) (x-axis). J) Boxplot showing the percentage of lysine (K) and arginine (R) residues in RNA- and DNA-binding proteins in killifish compared to the rest of the proteome ("Others", two-sample t-test with Welch's correction). K-L) Barplots showing selected proteins quantified by targeted proteomics based on parallel reaction monitoring (PRM) mass spectrometry (N=6-8 biological replicates, two sample t-test with Welch's correction). M and N) Examples of proteins with negative (M) and positive (N) decoupling in the optic tectum (N=8, 4 males and 4 females, MANOVA test). *P ≤ 0.05; **P ≤ 0.01, ***P ≤ 0.001, ****P ≤ 0.0001. Related to Fig. 1, and Tables S1 and S13.

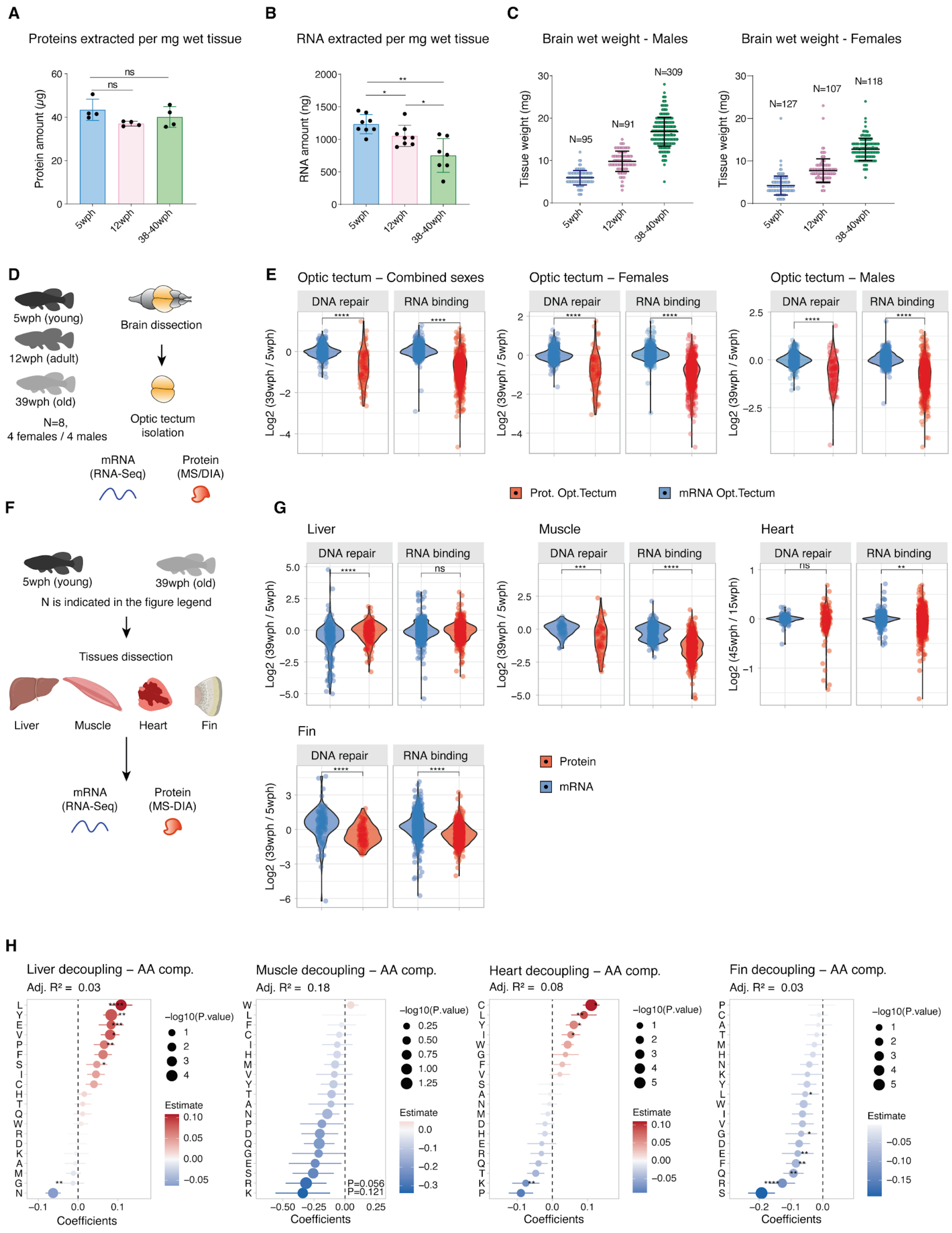

Figure S2: Age-related loss of proteins rich in basic amino acids independently of transcription in the optic tectum and across multiple tissues. A) Quantification of protein extracted per milligram of brain wet tissue using BCA assay. Each dot represents a pool 12-22 fish from both sexes (N=4 biological replicates, two sample t-test with Welch's correction). B) Amount of RNA extracted for milligrams of brain wet tissue measured by nanodrop spectrophotometer (N=7-8 biological replicates, two sample t-test with Welch's correction). C) Brain weight from young, adult, and old killifish, males (left) and females (right), the N number is stated on each graph. D) Workflow of the quantification of transcript and protein abundance in killifish's optic tectum. E) Transcript and protein fold changes for DNA repair, RNA-binding (N=8 biological replicates, two-sample Wilcoxon test). F) Workflow of the quantification of transcripts and protein abundance across different tissues

(N=8 biological replicates for liver, data from (*118*), and muscle, N=4 biological replicates for fin). For heart samples, the MZCS-08 killifish strain was used (N=3-5 biological replicates). Because of the strain difference, the age of young and old fish were 12-15wph and 40-46wph, respectively. G) Violin plot showing transcript and protein fold changes for RNA binding and DNA repair proteins across different tissues. Two-sample Wilcoxon test. H) Results of multiple linear regression analysis of protein-transcript decoupling scores across different tissues based on protein amino acid composition. F-test. *P ≤ 0.05; **P ≤ 0.01, ***P ≤ 0.001, ****P ≤ 0.0001. Related to Fig. 1, and Tables S2 and S3.

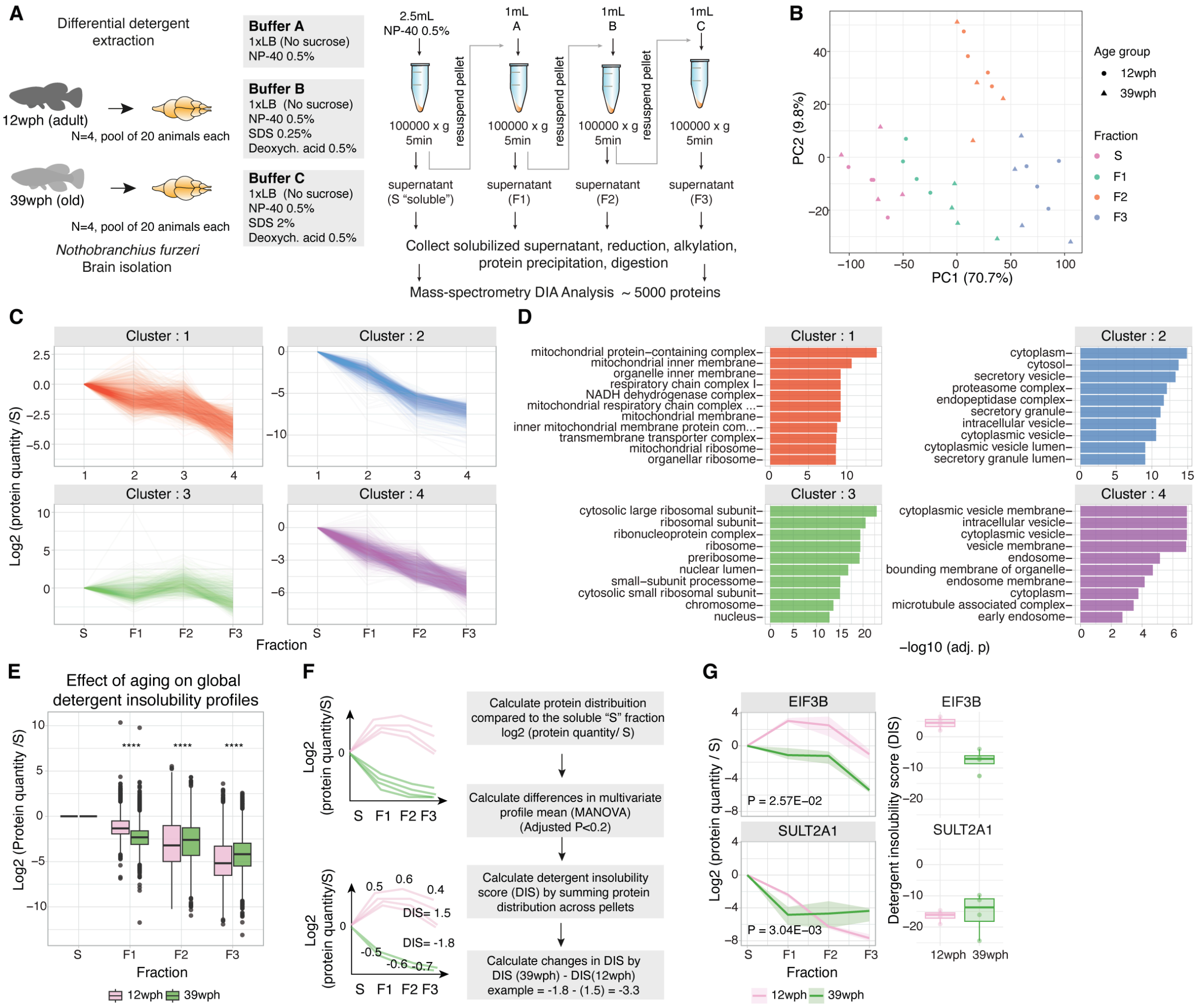

Figure S3: Protein detergent insolubility changes in the killifish aging brain. A) Scheme of the differential detergent extraction experiment. The protocol was adapted to brain tissue from (*22*). B) Principal component analysis based on proteomics data from fractions obtained by differential detergent extraction. C) K-means clustering of detergent insolubility profiles. On the y-axis, the log2 protein quantity is relative to the soluble "S" fraction, and each profile represents the median across both age groups and replicates (N=4 biological replicates). D) GO enrichment overrepresentation analysis (ORA) of proteins assigned to each cluster against the rest of the identified proteome. On the x-axis, the -log10 of the Adj. P is reported (Fisher's test with Holm correction). Colors refer to the different clusters displayed in panel C. E) Boxplot depicting detergent insolubility profiles for all the proteins quantified across age groups. The y-axis indicates the log2 transformed value of protein quantity in each fraction relative to the soluble (S) fraction. Two-sample Wilcoxon test. F) A computational strategy was used to calculate differences in detergent insolubility profiles across age groups. A MANOVA test was performed on each protein profile to detect significant changes in the multivariate mean between adult and old samples (N=4 biological replicates). The detergent insolubility score (DIS) was calculated by summing log2 protein quantities (relative to the soluble S fraction). Higher DIS indicates higher protein abundance in insoluble fractions (F1:F3) relatively to the soluble one (S). G) Example profiles of top hits proteins displaying changes in detergent insolubility with aging. EIF3B is an example of a protein that displays decreased detergent insolubility with age, while SULT2A1 displays increased detergent

insolubility with age. Left panel, the y-axis represents the log2 protein quantity in each fraction relative to the first soluble (S) fraction. Dark lines indicate the median between replicates, while shaded areas represent 50% of the replicate distribution (N=4 biological replicates). Right panel, boxplots showing the detergent insolubility score for the same proteins. *P ≤ 0.05; **P ≤ 0.01, ***P ≤ 0.001, ****P ≤ 0.0001. Related to Figs. 2 and S5, and Table S4.

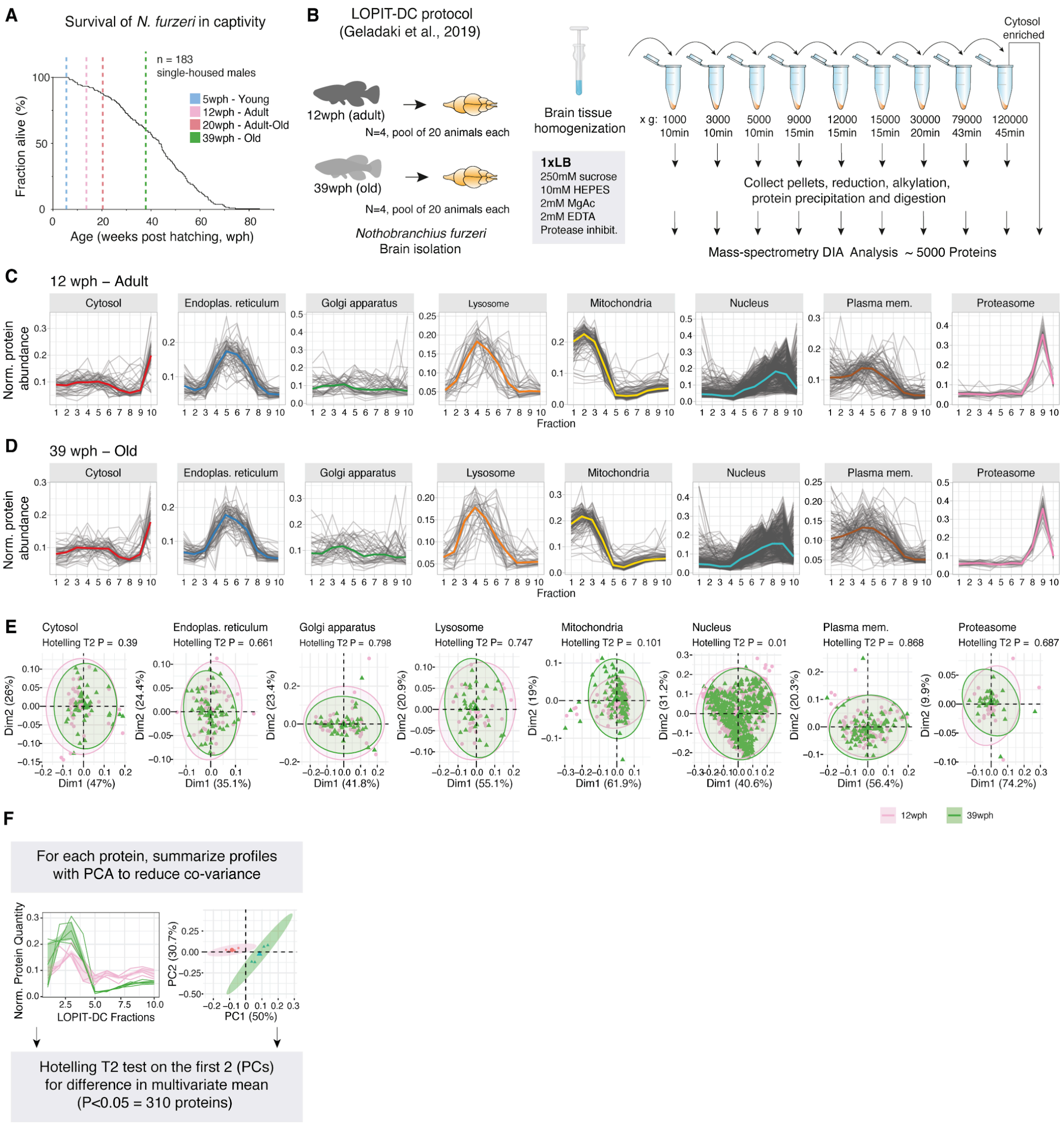

Figure S4: Subcellular fractionation of the killifish aging brain by LOPIT-DC. A) Survival curves of killifish MZM-0410 strain in captivity (data from (*118*)). Survival curves were generated by monitoring deaths starting at the age of 5wph, which corresponds to sexual maturity. This study includes data from four age groups highlighted by vertical dashed lines. The analyzed strain (MZM-0410) was kept in captivity and derived from a wild one with a median lifespan of 7-8 months. B) Scheme of the LOPIT-DC experiment (N=4 biological replicates) . The protocol was adapted to brain tissue from (*23*). C-D) Organelle markers protein profiles from LOPIT-DC. The y-axis indicates protein abundance estimates derived from mass spectrometry data. For each protein, the abundance in each fraction was normalized to the summed protein abundance across all fractions. Each profile represents the median protein abundance across replicates (N=4 biological replicates). A colored solid line highlights the median profiles of all the proteins assigned to a specific organelle. Profiles obtained from adult (C) and old (D) killifish are shown. E) Principal component analysis for different organelles markers in the LOPIT-DC fractions. Organelle markers from adult (12wph, pink) and old (39wph, green) are shown. Each dot

represents the median protein profile across (N=4 biological replicates) replicates for each condition. F) Computational strategy used to identify age-related changes in LOPIT-DC profiles. Related to Figs. 2 and S6, and Table S4.

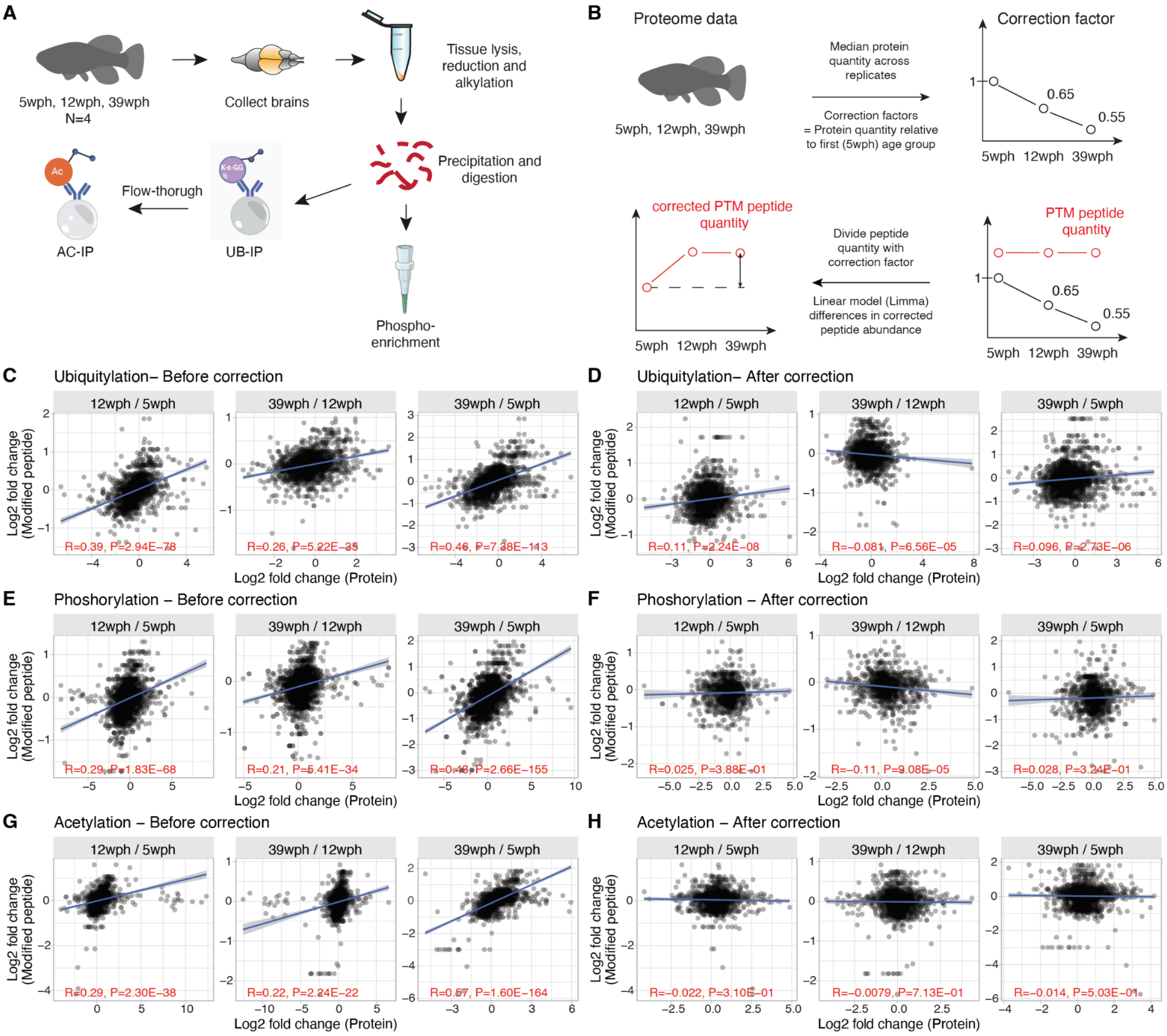

Figure S5: Analysis of protein post-translational modifications in the killifish brain aging. A) Workflow for the enrichment of post-translational modified peptides from killifish brain. B) Correction strategy for detecting stoichiometric changes in post-translationally modified peptides. Correction factors were computed for each protein and condition relative to the young (5wph) age group. Quantities of the modified peptides were divided by the corresponding protein correction factor, and age-related changes were tested using *limma* (*77*). C-H) Relationship between age-related abundance changes of modified peptides vs. corresponding protein before (left panels) and after (right panels) correction. The association between protein and modified peptides' changes was calculated using Pearson's product-moment. Related to Figs. 2 and S6, and Table S5.

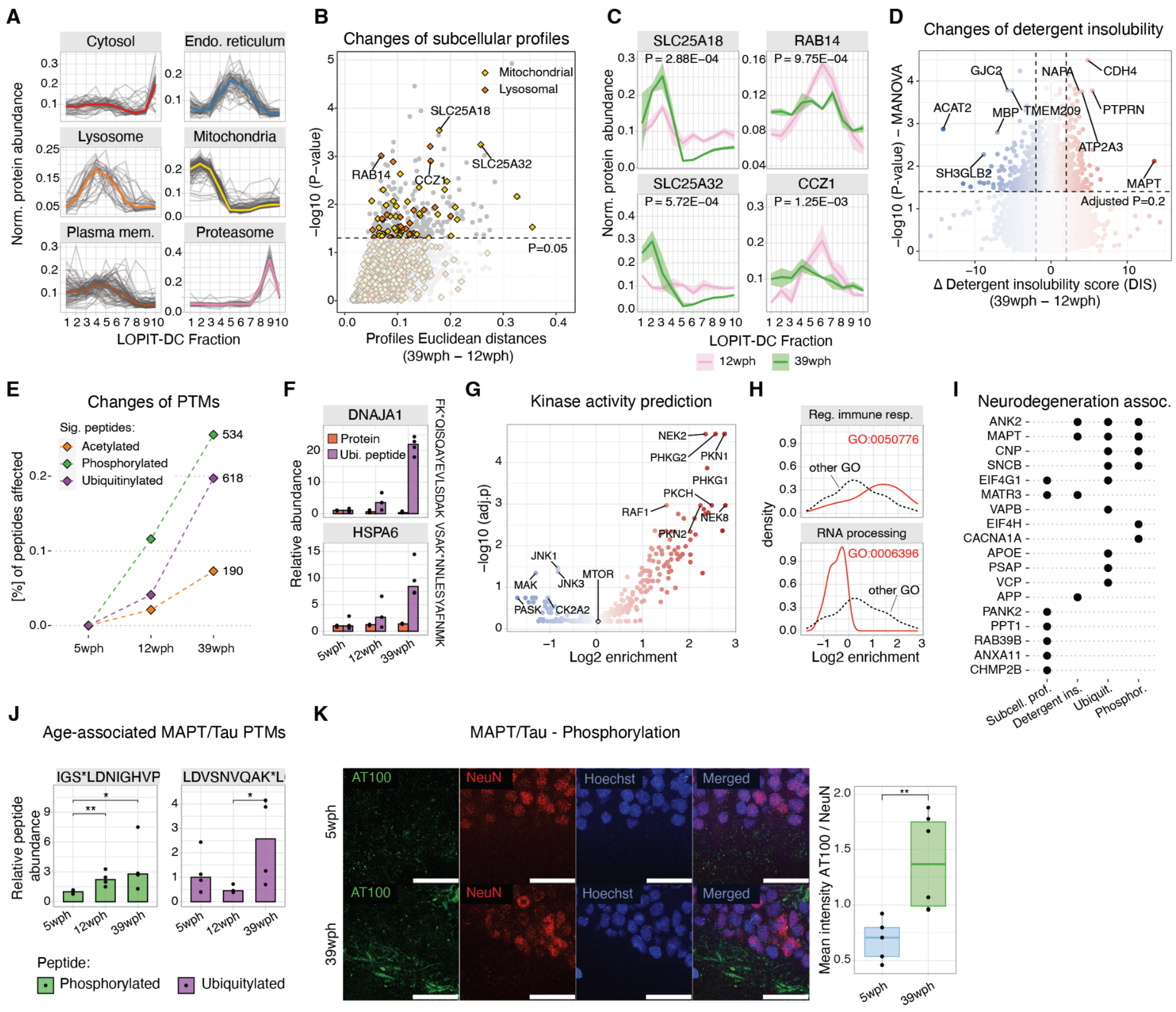

Figure S6: Aging affects protein subcellular localization, detergent insolubility, and post-translational modifications
A) Organelle markers protein profiles from LOPIT-DC (12wph). The x-axis indicates the different fractions of the LOPIT-DC experiment. The y-axis indicates protein distribution across fractions. A colored solid line highlights the median profiles of each organelle. B) Scatterplot showing age-related changes in LOPIT-DC profiles in the aging killifish brain. The x-axis displays the median replicate Euclidean distance of the profiles between the two age groups (N=4 biological replicates, Hotelling T-squared test). C) Examples of LOPIT-DC profiles for selected proteins with altered subcellular fractionation profiles. In each plot, the x-axis indicates the 10 fractions obtained from LOPIT-DC, and the y-axis indicates the total protein distribution along the 10 fractions for adult (pink) and old (green) killifish. Shaded areas indicate 50% of the replicate distribution (N=4 biological replicates, Hotelling T^2 test). D) Volcano plot showing protein detergent insolubility changes in the aging killifish brain. The x-axis indicates the differences in detergent insolubility score (DIS). Higher values indicate increased detergent insolubility in the old brain (N=4 biological replicates, MANOVA test). Significant changes were highlighted by dashed lines (Adj. MANOVA test $P < 0.2$ and absolute Δ DIS > 2). E) Post-translationally modified peptides affected by aging. The y-axis (left) indicates the percentage of significantly affected sites in each dataset when compared to the young samples ($P < 0.05$, N=3-4 biological replicates, moderated Bayes t-test). F) Barplots showing relative abundances of ubiquitylated peptides from DNAJA1 and HSPA6 across age groups (purple bars). The corresponding protein abundances were displayed as reference (red bars, N=3-4 biological replicates). G) Volcano plot showing changes in estimated kinase activity (using the algorithm from (*93*)) based on phosphoproteomics data from old vs. young killifish brains (N=4 biological replicates, Fisher's test with Holm's correction). H) Density distribution for kinases involved in the regulation of immune response (GO:0050776, upper panel) and RNA processing (GO:0006396, lower panel) against all other kinases from panel H. I) Heatmap summarizing alterations of proteins linked to neurodegenerative diseases across different datasets. Black dots mark significant alterations in each dataset ($P < 0.05$). J) Barplots displaying MAPT/Tau phosphorylated (green) and ubiquitylated (purple) peptides significantly affected by aging ($P < 0.05$). The values represent peptide abundances relative to the young age group after correction for protein changes (N=3-4 biological replicates, moderated Bayes t-test). K) Left panel, immunofluorescence stainings for phosphorylated (AT100) Tau in brain

cryo-sections of young and old killifish. The stainings were normalized to the intensity of NeuN to account for the different amounts of neuronal cells between young and old animals. Scale bars = 20µm. Right panel, quantification of phosphorylated Tau (N=5 biological replicates, two-sample Wilcoxon test). $^{*}P \leq 0.05$; $^{**}P \leq 0.01$, $^{***}P \leq 0.001$, $^{****}P \leq 0.0001$. Related to Fig.2, and Tables S4, S5 and S6.

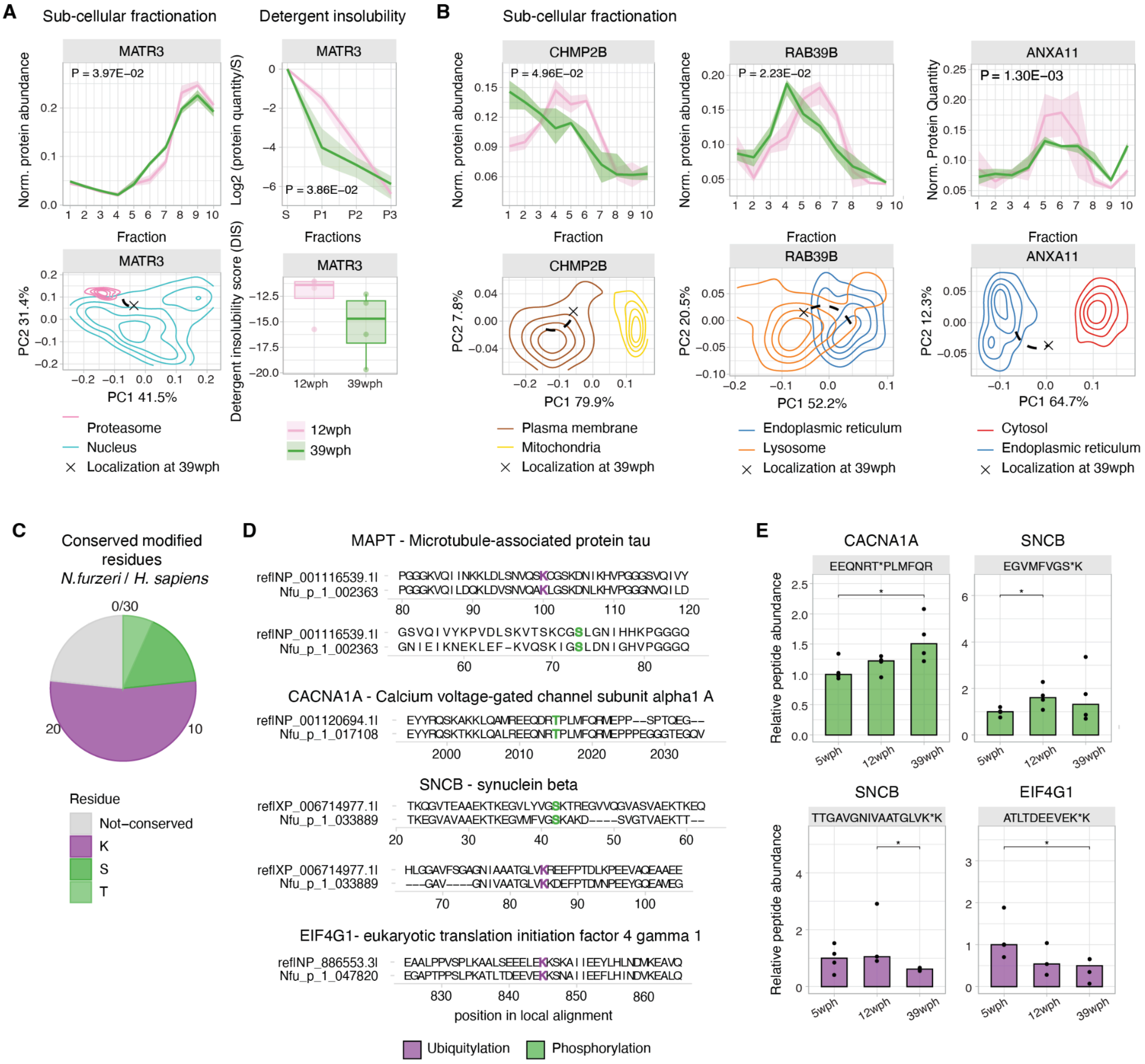

Figure S7: Age-associated alterations of proteins linked to human neurodegenerative disorders. A-B) Examples of proteins changing their subcellular localization profile or detergent insolubility. The top panels indicate subcellular fractionation profiles or detergent insolubility profiles. For subcellular fractionation, the x-axis indicates the 10 fractions obtained from LOPIT-DC, and the y-axis indicates the total protein distribution along the 10 fractions for adult (pink) and old (green) killifish. Shaded areas indicate 50% of the replicate distribution (N=4 biological replicates, Hotelling T^2 test). For detergent insolubility profiles, the x-axis indicates the different fractions: S=soluble, F1:F3=fractions after solubilization with buffers of increasing detergent strength (Fig. S3A). The y-axis indicates log2 protein quantities relative to the soluble (S) fraction. The shaded area indicates 50% of the distribution across (N=4 biological replicates, MANOVA test). In the bottom panels, the principal component analysis plots represent the relocalization for each protein based on LOPIT-DC profiles. The contour line represents the density distribution of the different organelles (calculated as the median between 12wph and 39wph), and the position of the protein at 39wph is highlighted with a cross. The organelles represented are the ones that possess the higher absolute changes in the log2 ratios between Euclidean distances from the protein in the two age groups. For panel A, the boxplot on the right side indicates the detergent insolubility score in the two age groups. C) Pieplot showing sites of peptide post-translational modification conserved between killifish and humans that display abundance changes with aging. Data refers to proteins involved in neurodegenerative diseases in humans. D) Local sequence alignments

between killifish proteins (bottom sequence) and best human BLAST hit (upper sequence) for different proteins involved in neurodegenerative diseases. Modified sites are highlighted in purple (ubiquitylation) and green (phosphorylation). E) Barplots displaying significant age-related changes in selected modified peptides ($P < 0.05$, N=3-4 biological replicates, moderated Bayes t-test). Peptide abundances are expressed relatively to the young (5wph) age group after correction for protein changes. Related to Figs. 2 and S6, and Tables S4, S5 and S6.

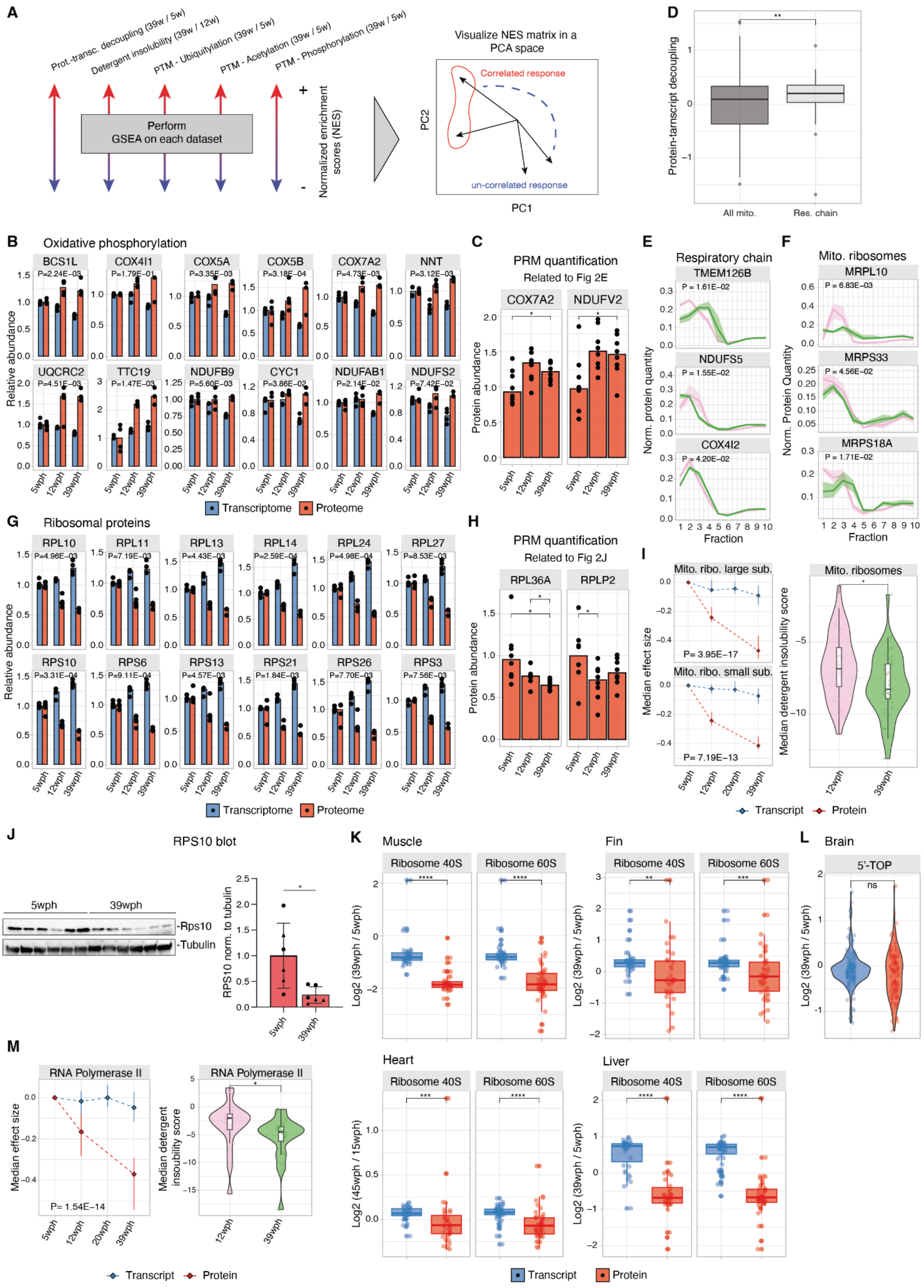

Figure S8: Age-related alterations of ribosomal and respiratory chain proteins. A) Scheme of data integration strategy. For each dataset, a gene set enrichment analysis (GSEA) was performed using GO terms for cellular components. The normalized enrichment scores (NES) from each dataset were combined into a matrix and used as input for principal component analysis. B) Barplot showing transcript and protein abundances for oxidative phosphorylation proteins. All the

values were normalized to the 5wph (young) age group (N=3-4 biological replicates, MANOVA test). C) Barplots showing selected mitochondrial proteins quantified by targeted proteomics based on parallel reaction monitoring (PRM) mass spectrometry (N=8 biological replicates, two sample t-test with Welch's correction). D) Boxplot depicting the distribution of protein-transcript decoupling values (as defined in Fig. 1A) for oxidative phosphorylation (light gray) proteins against the rest of the mitochondrial proteome (dark gray). Two-sample Wilcoxon test. E-F) Examples of mitochondrial proteins that display changes in subcellular fractionation with aging. In each plot, the x-axis indicates the 10 fractions obtained from LOPIT-DC, and the y-axis indicates the total protein distribution along the 10 fractions for adult (pink) and old (green) killifish. Shaded areas indicate 50% of the replicate distribution (N=4 pools of 20 animals each, Hotelling T^2 test). G) Barplot showing transcript and protein abundances for cytoplasmic ribosomal proteins. All the values were normalized to the 5wph (young) age group (N=3-4 biological replicates, MANOVA test). H) Barplots showing selected ribosomal proteins quantified by targeted proteomics based on PRM mass spectrometry (N=8 biological replicates, two sample t-test with Welch's correction). I) Left panel: line plots for mitochondrial ribosome members' transcript (blue) and protein (red) median abundance across age groups . Each point represents the median of the log2 ratio of the quantities relative to the young (5wph) age group. Vertical lines indicate 50% of the distributions (N=3-4 biological replicates, MANOVA test). Right panel: violin plot displaying detergent insolubility scores for mitochondrial ribosome proteins (GO:0005761). Each dot represents the median insolubility score of each protein (N=4 biological replicates, two-sample Wilcoxon test). J) Immunoblot for RPS10 protein level across age groups. RPS10 band intensities were normalized to tubulin as loading control (N=6 biological replicates, triangles indicate males, circles females, two sample t-test with Welch's correction). K) Boxplot showing the effect of aging on cytoplasmic ribosomal proteins across different tissues. The blue color indicates transcript changes, while the red color indicates protein changes (two-sample Wilcoxon test). L) Transcript and protein fold changes for 5'-TOP genes (*114*). Two-sample Wilcoxon test. M) Left panel: line plot for RNA polymerase II complex members' transcript (blue) and protein (red) median abundance across age groups. Each point represents the median of the log2 ratio of the quantities relative to the young (5wph) age group. Vertical lines indicate 50% of the distributions (N=3-4 biological replicates, MANOVA test). Right panel: violin plot displaying detergent insolubility scores for proteins of the RNA polymerase II complex (GO:0016591). Each dot represents the median insolubility score of each protein (N=4 biological replicates, two-sample Wilcoxon test). *$P \leq 0.05$; **$P \leq 0.01$, ***$P \leq 0.001$, ****$P \leq 0.0001$. Related to Fig. 2, and Tables S3, S4 and S13 .

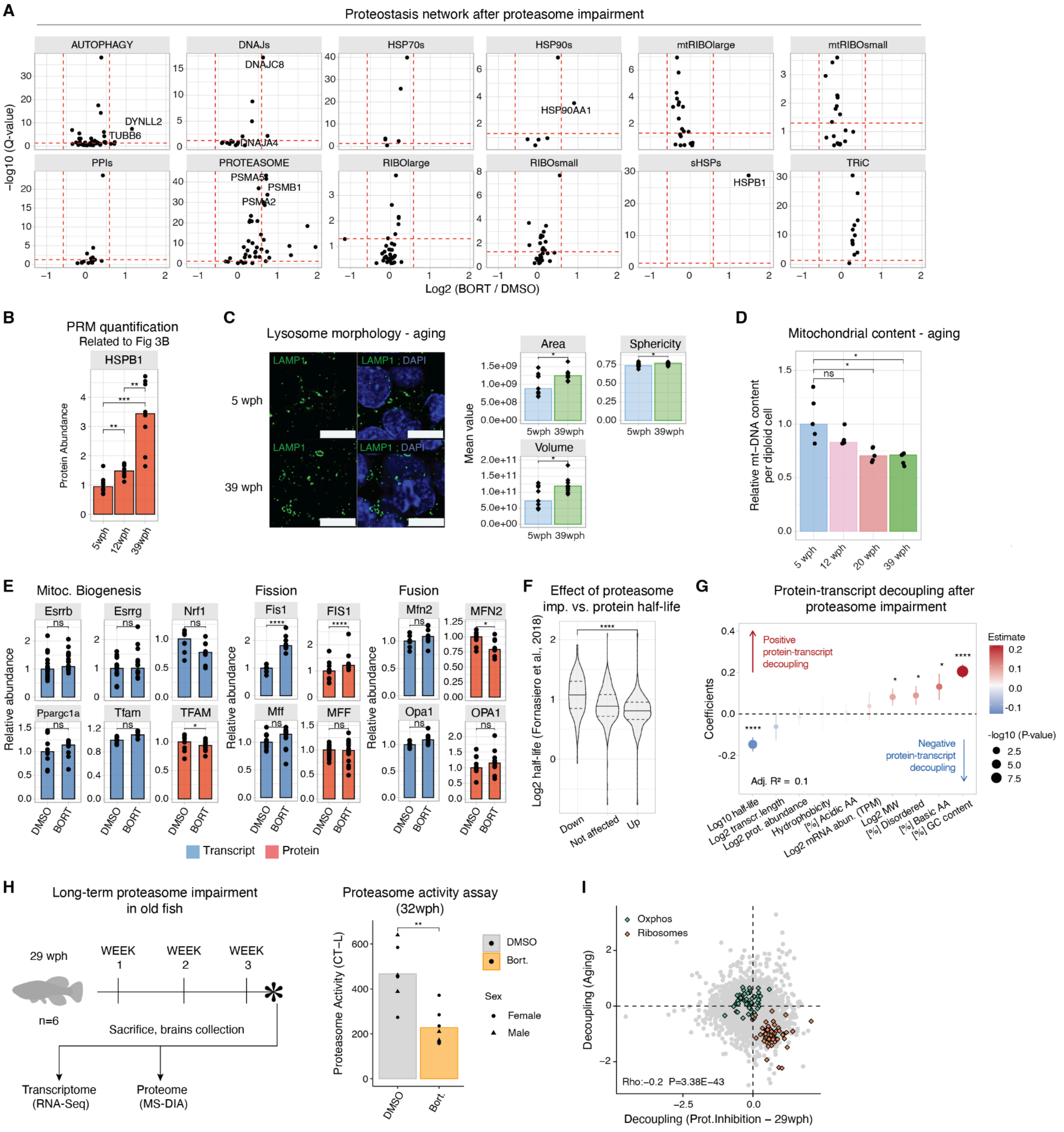

Figure S9: Effect of proteasome inhibition on the killifish brain. A) Protein abundance changes induced by proteasome inhibition for components of the proteostasis network. B) Barplots showing HSPB1 quantification by targeted proteomics based on parallel reaction monitoring (PRM) mass spectrometry (N=8 biological replicates, two sample t-test with Welch's correction). C) Left panel: immunofluorescence stainings for lysosome (LAMP1) in brain cryo-sections of young (light blue) and old (green) killifish. Scale bars = 5µm. Right panel: quantification of lysosome morphology features in young (light blue) and old (green) samples. The y-axis represents the mean value of the different morphology features in each of the replicates (N=9-12 biological replicates, two-sample Wilcoxon test). D) Quantification of mitochondrial DNA (mt-DNA) from brains of killifish of different ages. mtDNA copy number was calculated using real-time quantitative PCR with primers for 16S rRNA mitochondrial gene and cyclin-dependent kinase inhibitor 2a/b (Cdkn2a/b) nuclear gene for normalization (N=5 biological replicates, two-sample Wilcoxon test). E) Barplot showing protein (red) and transcript (blue) abundance changes of selected mitochondrial proteins following proteasome inhibition. Asterisks indicate Q values from Spectronaut for proteins and Adj. P values from DESeq2 for transcripts (N=10). F) Violin plot showing the distribution of up and down-regulated proteins in response to proteasome inhibition against their half-life as quantified in (*18*). Two-samples Wilcoxon test. G) Multiple linear regression analysis of decoupling scores in response to proteasome inhibition based on biophysical features of transcripts or proteins as predictors. F-test. H) Left panel: old killifish (29wph, N=6 biological replicates) received weekly intraperitoneal injections of proteasome inhibitor bortezomib or DMSO control. Right panel:

quantification of chymotrypsin-like proteasome activity (N=6 biological replicates, two-sample Wilcoxon test). I) Decoupling scores comparison between aging and proteasome inhibition performed at 29wph for respiratory chain (green) and ribosomal (orange) proteins.*$P \leq 0.05$; **$P \leq 0.01$, ***$P \leq 0.001$, ****$P \leq 0.0001$. Related to Fig. 3, and Tables S7, S8 and S13.

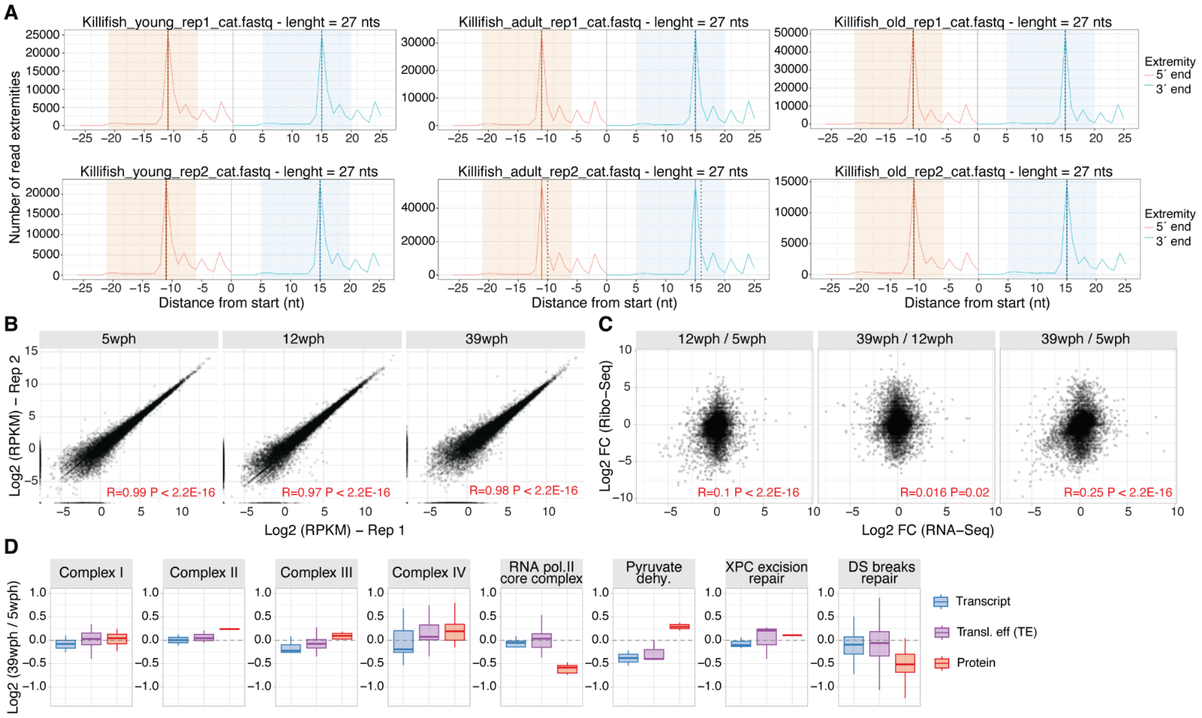

Figure S10: Ribosome profiling in the killifish aging brain. A) Tri-nucleotide plot showing characteristic triplet periodicity in Ribo-Seq data. The x-axis represents the distance from the starting codon (in nucleotides, nt), and the y-axis represents the number of extremities (5' or 3' ends of the reads) mapping to respective positions. B) Scatterplot showing the correlation between replicates for the Ribo-Seq experiment. RPKM: reads per kilobase of transcript per million mapped reads. R value indicates the Pearson's R correlation coefficient. C) Scatterplot showing the correlation between log2 fold changes for ribosome occupancy (y-axis) and changes in the transcriptome (x-axis) for different age comparisons. R value indicates the Pearson's R correlation coefficient. D) Boxplot displaying age-related changes of transcript level, translation efficiency and protein abundance for selected protein complexes. Related to Fig. 4 and Table S9.

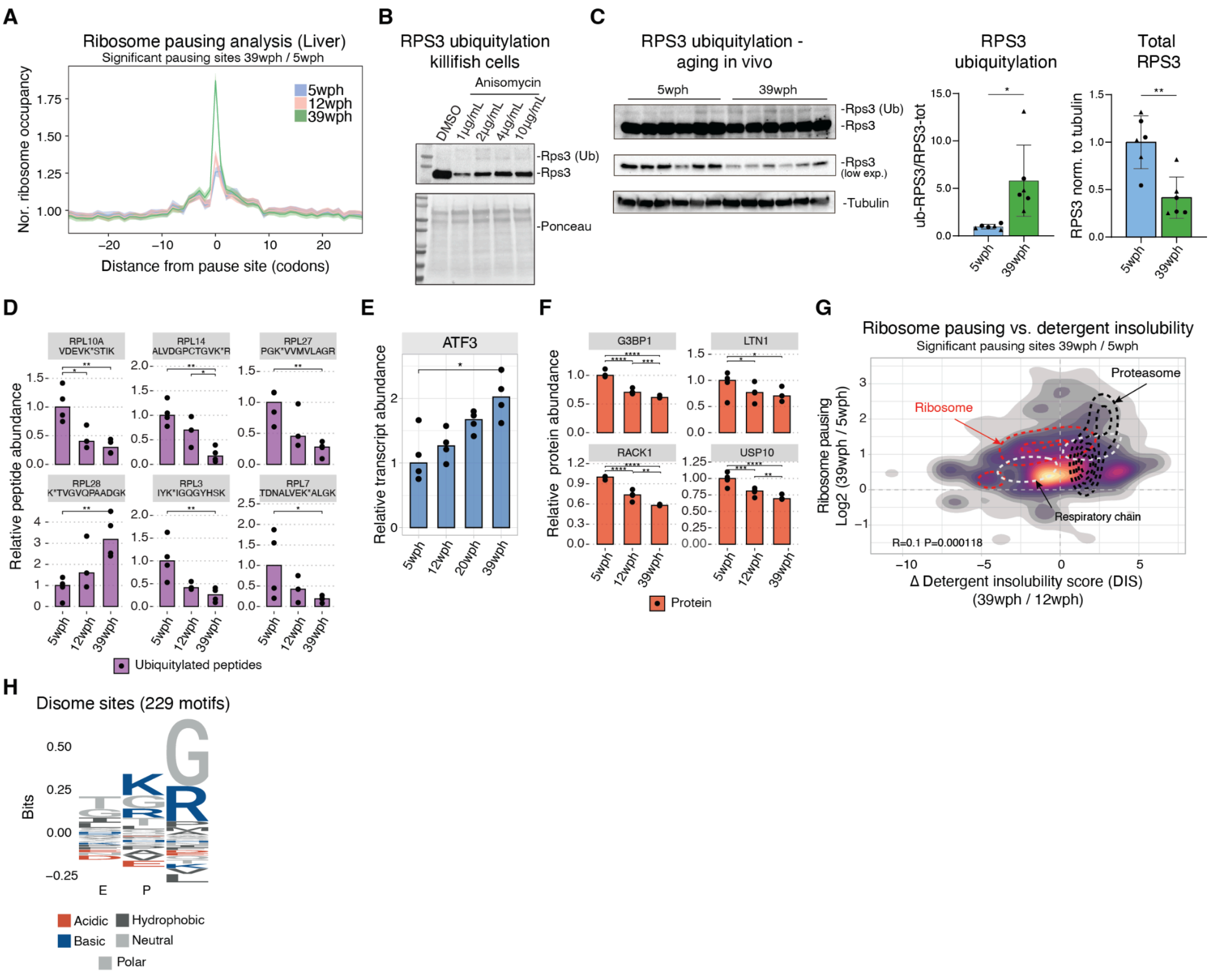

Figure S11: Ribosome pausing during killifish aging. A) Line plot showing the normalized ribosome distribution at pausing sites across different age groups in killifish liver. B) Immunoblot for RPS3 ubiquitylation in killifish cells treated for 24 hours with anisomycin, a compound that inhibits translation elongation and causes ribotoxic stress (*130*). C) Immunoblot for RPS3 protein level and ubiquitylation in brains from different age groups. Barplot on the right shows total RPS3 abundance normalized to tubulin as loading control. Barplot on the left shows the ratio between total and ubiquitylated RPS3 (N=6 biological replicates, triangles indicate males, circles females, two sample t-test with Welch's correction). D) Barplots displaying ubiquitylated peptides from ribosomal proteins affected by aging ($P < 0.05$). The values represent peptide abundances relative to the young age group after correction for protein changes (N=3-4 biological replicates, moderated Bayes t-test). E) Barplot showing normalized transcript abundance (relative to young, 5wph, age group) for the Activating Transcription Factor 3 (Atf3) (N=4 biological replicates, Wald test from the DESeq2 package (*85*)). F) Barplot showing normalized protein abundance (relative to young, 5wph, age group) for proteins involved in ribosome quality control (RQC) pathway. Asterisks indicate P-values from Spectronaut differential abundance analysis (N=3-4 biological replicates). G) 2-D density plot showing the relation between significant changes in pausing displayed on the y-axis and changes in detergent insolubility (x-axis). Density distributions are based on individual pausing sites that are significantly affected by aging (Adj. $P < 0.05$). Multiple significant pausing sites might map to the same transcript. Contours: cytoplasmic ribosomes (red), proteasome (black), and oxidative phosphorylation protein complexes (white). H) Logo plot of amino acids being decoded at strong pause sites (pause score > 6) that display disome peaks unique to 39wph as shown in Fig. 4F. E: tRNA exit site; P: peptidyl-tRNA binding site; A: aminoacyl-tRNA binding site. *$P \leq 0.05$; **$P \leq 0.01$, ***$P \leq 0.001$, ****$P \leq 0.0001$. Related to Fig. 4 and Table S9.

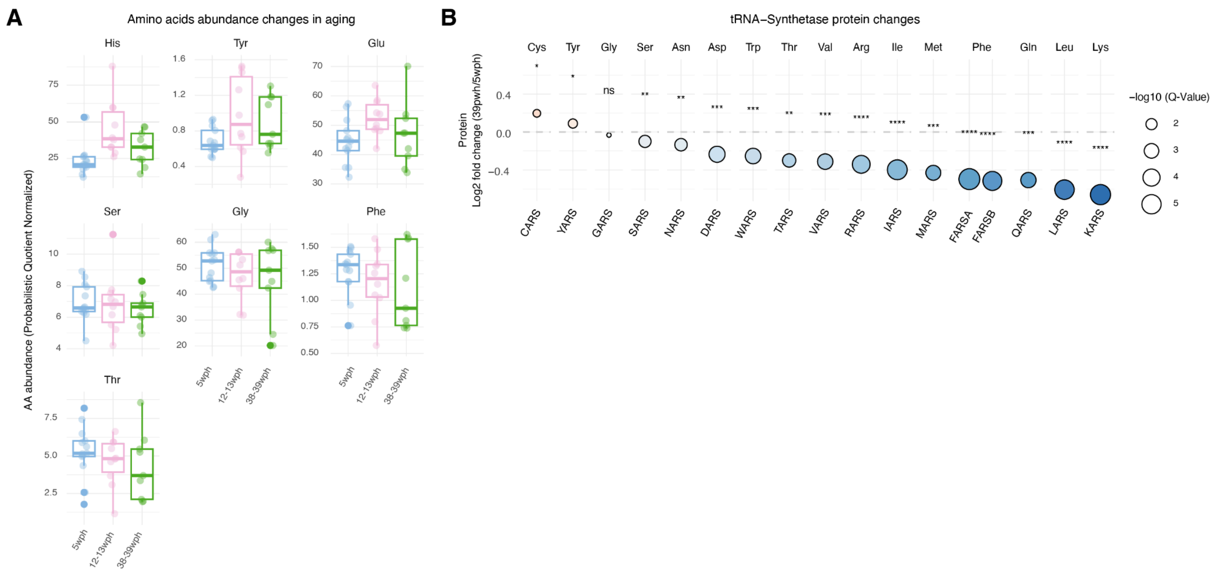

Figure S12: Amino acids and t-RNA synthetase in the aging killifish brain. A) Boxplot displaying quantification of amino acids that do not display age-related changes in killifish brains. Amino acid abundances were reported as PQN values. Two-sample t-test with Welch's correction (N=6 biological replicates). B) tRNA synthetase protein changes in the aging killifish brain. Cases when multiple killifish proteins mapped to one human ortholog were averaged for display purposes (N=3-4 biological replicates, Q values from Spectronaut differential abundance analysis). *P ≤ 0.05; **P ≤ 0.01, ***P ≤ 0.001, ****P ≤ 0.0001. Related to Fig. 5, and Tables S10 and S11.

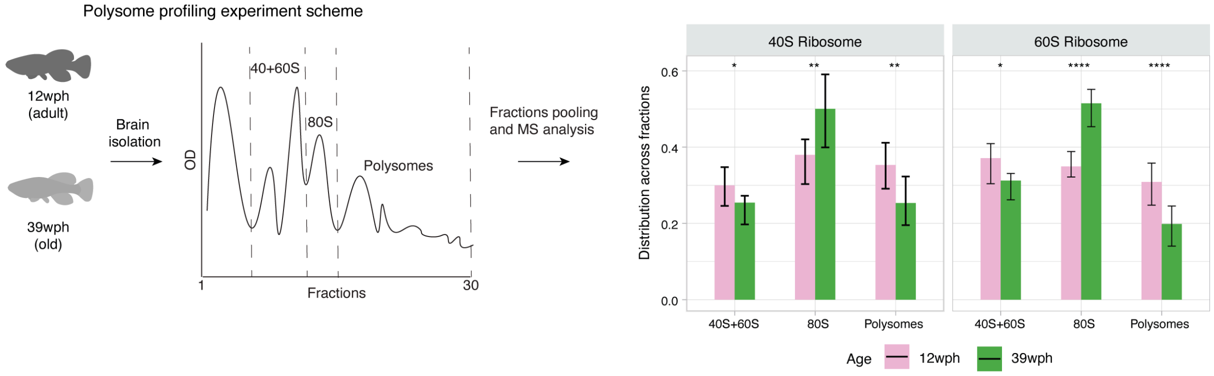

Figure S13: Polysome profiling on killifish aging brain. Left: schematic representation of polysome profiling experiment is killifish brain. Ribosome species were separated on a 10%-50% sucrose gradient. Fractions corresponding to the 40+60S, 80S, and polysomes were pooled and analyzed by quantitative mass spectrometry. Right: barplots showing the distribution of abundance of ribosomal proteins in different. Error bars represent 50% of the ribosomal proteins' distribution. Two sample Wilcoxon test. *P ≤ 0.05; **P ≤ 0.01, ***P ≤ 0.001, ****P ≤ 0.0001. Related to Fig. 6.
